# Structure, function and connectivity fingerprints of the frontal eye field versus the inferior frontal junction: A comprehensive comparison

**DOI:** 10.1111/ejn.15393

**Published:** 2021-08-04

**Authors:** Marco Bedini, Daniel Baldauf

**Affiliations:** ^1^ Center for Mind/Brain Sciences University of Trento Trento Italy

**Keywords:** brain connectivity, prefrontal cortex, spatial versus non‐spatial selection, visual attention, working memory

## Abstract

The human prefrontal cortex contains two prominent areas, the frontal eye field and the inferior frontal junction, that are crucially involved in the orchestrating functions of attention, working memory and cognitive control. Motivated by comparative evidence in non‐human primates, we review the human neuroimaging literature, suggesting that the functions of these regions can be clearly dissociated. We found remarkable differences in how these regions relate to sensory domains and visual topography, top‐down and bottom‐up spatial attention, spatial versus non‐spatial (i.e., feature‐ and object‐based) attention and working memory and, finally, the multiple‐demand system. Functional magnetic resonance imaging (fMRI) studies using multivariate pattern analysis reveal the selectivity of the frontal eye field and inferior frontal junction to spatial and non‐spatial information, respectively. The analysis of functional and effective connectivity provides evidence of the modulation of the activity in downstream visual areas from the frontal eye field and inferior frontal junction and sheds light on their reciprocal influences. We therefore suggest that future studies should aim at disentangling more explicitly the role of these regions in the control of spatial and non‐spatial selection. We propose that the analysis of the structural and functional connectivity (i.e., the connectivity fingerprints) of the frontal eye field and inferior frontal junction may be used to further characterize their involvement in a spatial (‘where’) and a non‐spatial (‘what’) network, respectively, highlighting segregated brain networks that allow biasing visual selection and working memory performance to support goal‐driven behaviour.

## Graphical abstract

Paradigms that involve spatial mechanisms (red outline) consistently engage the frontal eye field. In contrast, paradigms designed to disentangle non‐spatial (i.e., feature‐ and object‐based) mechanisms from spatial ones (blue outline) predominantly engage the inferior frontal junction. We examine how systematic differences in the structure, function and connectivity fingerprints of these regions underlie this crucial dissociation across attention, working memory and cognitive control functions.

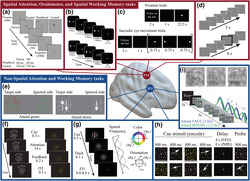

**Abbreviations**
ALE: activation likelihood estimation
BA: Brodmann area
BOLD: blood oxygen level dependent
cTBS: continuous theta burst stimulation
DAN: dorsal attention network
dMRI: diffusion magnetic resonance imaging
EEG: electroencephalography
FEF: frontal eye field
FFA: fusiform face area
fMRI: functional magnetic resonance imaging
iFEF: inferior frontal eye field
IFG: inferior frontal gyrus
IFJ: inferior frontal junction
IFJa: anterior inferior frontal junction
IFJp: posterior inferior frontal junction
IFS: inferior frontal sulcus
iPCS: inferior precentral sulcus
IPS: intraparietal sulcus
MACM: meta‐analytic connectivity modelling
MEG: magnetoencephalography
MFG: middle frontal gyrus
MMP1: multimodal parcellation 1.0
MVPA: multivariate pattern analysis
PCS: precentral sulcus
PET: positron emission tomography
PFC: prefrontal cortex
plPFC: posterior lateral prefrontal cortex
PPA: parahippocampal place area
ROI: region of interest
RSVP: Rapid Serial Visual Presentation
RT: reaction time
SFS: superior frontal sulcus
SLF: superior longitudinal fasciculus
sPCS: superior precentral sulcus
SPL: superior parietal lobule
TMS: transcranial magnetic stimulation
TPJ: temporoparietal junction
VAN: ventral attention network
vlPFC: ventrolateral prefrontal cortex

## 1 INTRODUCTION

It is remarkable to what extent primates have evolved the cognitive faculties that allow their behaviour to be guided not only by their immediate surroundings and environment but also by their future goals and action plans (Fuster, 2001). The successful orchestration of these goal‐driven behaviours requires the adaptive coding of contextual information (Duncan, 2001), and the integration of this information in order to bias processing in other brain areas (Barceló et al., 2000) and to finally map sensory input into coherent behavioural sequences (Miller & Cohen, 2001). Our ability to prioritize important sensory information (a function named selective attention; Carrasco, 2011; Posner, 1980) arguably plays a crucial role in all the former processes. Visual selection, for example, is thought to be guided top‐down by ‘attentional templates’ (Desimone & Duncan, 1995), which can assume highly divergent representational formats, for example, in the form of spatial as opposed to feature‐ and object‐based attention (hereafter referred to spatial and non‐spatial attention/selection; Carrasco, 2011; see also Moore & Zirnsak, 2015). In a typical everyday life situation, we may know how the object we are searching for looks like (e.g., our bicycle), but its current location (if we forgot where we parked it). Conversely, we often remember an objects' location, but due to the environmental clutter, we may not recognize it until we focus our spatial attention there. In both instances, it is the specific ‘attentional template’ that we adopt to guide our search that boosts the representation of the object's location, or its identity, allowing us to select it and move on to our next goal. A fundamental question in cognitive neuroscience is, therefore, where and how attentional priorities are computed in the primate brain (Desimone & Duncan, 1995; Itti & Koch, 2001), and how spatial and non‐spatial selective mechanisms interact to enable flexible and efficient goal‐driven behaviour (O'Reilly, 2010; Rao et al., 1997).

The prefrontal cortex (PFC) has long been recognized to be crucially involved in many aspects of such complex, organized behaviour in non‐human and human primates (Fuster & Alexander, 1971; Luria, 1966; Norman & Shallice, 1986; Rainer et al., 1998). In the past decades, several investigators, most notably Patricia S. Goldman‐Rakic, advanced the hypothesis that also the PFC can be segregated into functionally distinct domains (Goldman‐Rakic, 1996; Romanski, 2004; Wilson et al., 1993). In particular, what motivated Goldman‐Rakic to posit the domain‐specific organization of the PFC was the observation of substantial differences in the selectivity of neurons (Wilson et al., 1993), as well as their anatomical connectivity patterns (reviewed in Goldman‐Rakic, 1996). Together, these patterns of selectivity and connectivity suggested that the posterior lateral PFC (plPFC) contained two segregated regions that belonged to the global dorsal and the ventral visual streams (Goodale & Milner, 1992; Mishkin et al., 1983), and which predominantly encoded spatial and object information, respectively (Goldman‐Rakic, 1996; Scalaidhe et al., 1997, 1999; Wilson et al., 1993). More recently, this framework was further supported by mounting evidence in primates (Bichot et al., 2015, 2019; Constantinidis & Qi, 2018; Meyer et al., 2007, 2011; Riley et al., 2017; Schwedhelm et al., 2020) and was expanded thanks to neuroimaging methods in humans, in the context of studies focusing on understanding how the PFC maintains top‐down control over visual selection and encodes behaviourally relevant stimuli in various experimental tasks (Baldauf & Desimone, 2014; Chan, 2013; O'Reilly, 2010; Serences, 2016).

This review will focus on the organization of the plPFC in humans. In particular, we will compare in detail two regions that are implicated in visual attention, working memory and cognitive control, namely, the frontal eye field (FEF) and the inferior frontal junction (IFJ). We would therefore like to stress that the goal of this paper is not to provide a systematic review of FEF and IFJ per se, as these are already available elsewhere (the FEF has been reviewed extensively in Petit & Pouget, 2019; Vernet et al., 2014; the IFJ was reviewed in Brass et al., 2005). Rather, in the following, our goal will be to highlight the properties that reveal crucial differences in the structure, function and connectivity of these two areas and to systematically compare them to uncover their functional specialization. In the last decade, research has been able to successfully disentangle the specific contribution of both of these areas to visual attention and working memory through the careful combination of neuroimaging methods (functional magnetic resonance imaging [fMRI] and magnetoencephalography [MEG]) with more sophisticated data analysis tools (i.e., multivariate pattern analysis [MVPA], Haxby et al., 2001; and functional and effective connectivity metrics, e.g., Baldauf & Desimone, 2014; Nee & D'Esposito, 2016; Sneve et al., 2013; Vossel et al., 2012; Wen et al., 2012; Zhang et al., 2018). Therefore, we aim to understand how the structure, function and connectivity fingerprints of the FEF and the IFJ constrain and shape their role in these cognitive functions and the underlying brain networks (Table 1).

**TABLE 1:** List of the main abbreviations used in the text and figures

Abbreviation	Full name	Abbreviation	Full name
ALE	Activation likelihood estimation	MFG	Middle frontal gyrus
BA	Brodmann area	MMP1	Multimodal parcellation 1.0
DAN	Dorsal attention network	MVPA	Multivariate pattern analysis
dMRI	Diffusion magnetic resonance imaging	PCS	Precentral sulcus
FEF	Frontal eye field	PFC	Prefrontal cortex
FFA	Fusiform face area	PPA	Parahippocampal place area
IFG	Inferior frontal gyrus	SLF	Superior longitudinal fasciculus
IFJ	Inferior frontal junction	SFS	Superior frontal sulcus
IFS	Inferior frontal sulcus	SPL	Superior parietal lobule
IPS	Intraparietal sulcus	TPJ	Temporoparietal junction
MACM	Meta‐analytic connectivity modelling	VAN	Ventral attention network

*Note*: Brain topology abbreviations: a, anterior; p, posterior; r, rostral; c, caudal; i, inferior; s, superior; m, medial; l, lateral; d, dorsal; v, ventral. For example, the plPFC is the posterior lateral prefrontal cortex. These abbreviations are always lower case, whereas upper case characters will be used exclusively to denote brain regions, gyri and sulci throughout the review, if not indicated otherwise.

According to the classic definition, these three criteria (structure, function and connectivity) define the concept of a cortical region, along with topographic (i.e., somatotopic or retinotopic) organization (Eickhoff, Constable, & Yeo, 2018). However, the latter is generally deemed less important beyond unimodal sensory cortices (Eickhoff, Constable, & Yeo, 2018). In contrast to early visual areas, where all these cortical features are generally well aligned, and inter‐individual differences are small, leading to a reliable way to parcel the underlying brain structures (Abdollahi et al., 2014; Sereno et al., 1995), in the PFC studies show that the alignment between cortical features becomes less apparent, and even the relative spatial arrangement of brain areas can sometimes vary substantially between individuals (Eickhoff, Yeo, & Genon, 2018). Thus, to understand the modular organization of the PFC, a multimodal approach capable of combining information about all the most relevant cortical properties becomes crucial (Glasser et al., 2016; Van Essen et al., 2019). Another relevant concept in the present context is the idea that the function of a brain region is heavily constrained by its intrinsic and extrinsic connectivity fingerprints (Passingham et al., 2002). Indeed, only by describing the connectivity fingerprints of each region, we are able to fully understand the most relevant aspects of functional specialization of the regions in the PFC, and in particular, those that are fundamentally underlaid by the differential selectivity of their neural populations to specific sensory inputs. Together, these two principles will guide the organization of this review in three main sections: the first, in which we will compare the structure of FEF and IFJ, the second, in which we will compare the function of FEF and IFJ, and a final third section, in which we will describe and contrast their connectivity fingerprints.

### 1.1 History and definition

The discovery of a brain area involved in the oculomotor aspects of behaviour in the monkey dates back to the work of the Scottish neurophysiologist David E. Ferrier in the last half of the 19th century. He reported that: ‘In the superior frontal convolution, in advance of the centre, for certain forward movements of the arm, as well as in the corresponding part of the middle frontal convolution, are areas, stimulation of which causes lateral (crossed) movements of the head and eyes and dilatation of the pupils’ (Ferrier, 1875; cited in Vernet et al., 2014). Thus, the way the FEF has been labelled directly reflects its hypothesized oculomotor function, a fact that may have contributed to obscure the large discrepancy between the way this region has been traditionally mapped in primates (i.e., by microstimulation techniques; Bruce et al., 1985) and the way it was localized in early neuroimaging studies, and consequently ‘translated’ into the human brain's taxonomic language (Paus, 1996; Petit & Pouget, 2019). To date, the FEF has been the subject of hundreds of studies both in monkeys and in humans (reviewed in Petit & Pouget, 2019, Tehovnik et al., 2000, and Vernet et al., 2014). Despite the considerable efforts of the neuroscientific community to characterize the FEF, this region's structure, function and connectivity in humans are often debated and are not yet fully understood. The IFJ, in contrast, came under the spotlight of the neuroscientific community only much more recently. According to Sundermann and Pfleiderer (2012), although this region has been the object of many neuroimaging studies investigating various components of cognitive control, task‐switching and working memory, researchers have often missed reporting the IFJ as a segregated brain region. The IFJ was first described with the current label in a series of influential studies by Brass, Derrfuss, von Cramon and colleagues (Brass & von Cramon, 2002, 2004; Derrfuss et al., 2004, 2005; see also Bunge et al., 2003, and Sylvester et al., 2003, for a converging characterization of the function of the IFJ; reviewed in Brass et al., 2005) and implicated in a cognitive control network (Cole & Schneider, 2007). In recent years, the IFJ has generated increased interest due to its involvement in a surprising variety of high‐level cognitive functions, such as top‐down visual attention (Baldauf & Desimone, 2014), working memory (Zanto et al., 2010) and the implementation of novel task instructions (Muhle‐Karbe et al., 2017), thus firmly positioning it within the multiple‐demand system of the brain (Assem et al., 2020; Duncan, 2010; Fedorenko et al., 2013).

**FIGURE 1:**
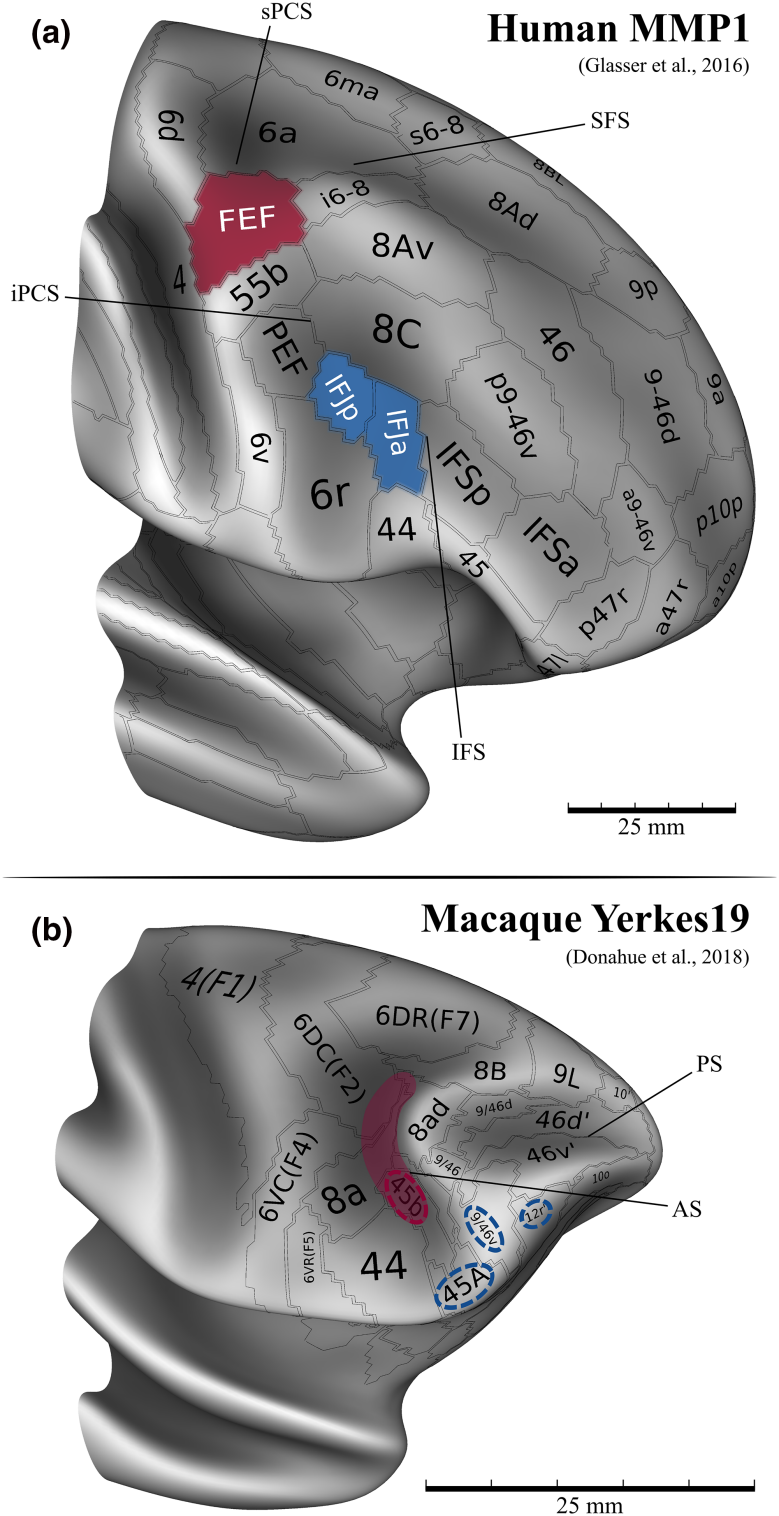
Putative homologies between the human and the macaque PFC displayed on the human MMP1 (a) and the macaque Yerkes19 atlases (b). The homologies between the human and the macaque PFC are, due to the lack of similar sulcal morphology (because the macaque brain has a single principal sulcus, in contrast to the human brain, which has two major sulci, the SFS and the IFS), best inferred based on structural and/or functional criteria, rather than morphological information. In the MMP1, the typical localization of the IFJ relative to the sulci likely corresponds to the IFJp (a) (see Section 2.2 for a detailed discussion). According to the study by Donahue et al. (2018), the human FEF would be the homologue of area 45b in the macaque (Schall et al., 1995), but it may also overlap with other brain regions (viz., 8a and 6DC(F2); see, e.g., fig. 1 in Schall, 2015). In the studies by Bichot et al. (2015, 2019), the authors identified a ventral prearcuate region (VPA) that was proposed as the human IFJ homologue and that overlapped with areas 46v, 45A and 12. Based on their injection sites, three regions that may correspond to the VPA were highlighted on the Yerkes19 atlas (b) (the regions 45A, 9/46v and 12r′ from the composite PFC parcellation by Donahue et al., 2018; PS, principal sulcus; AS, arcuate sulcus; both (a) and (b) were adapted from the datasets available in BALSA at https://balsa.wustl.edu/; Van Essen et al., 2017). At present, it is however unclear whether cytoarchitectonic maps in the macaque are appropriate for inferring homologies with the human PFC (see Section 2.1 for a discussion). In Section 5, we highlight some of the most intriguing proposals that aim at resolving these comparative neuroanatomical issues

Moreover, research has started to identify structural and functional correspondences between the ventrolateral PFC (vlPFC) in macaques and humans, enabling insightful cross‐species comparisons (Figure 1; Bichot et al., 2015, 2019; Donahue et al., 2018; Neubert et al., 2014; Schwedhelm et al., 2017, 2020). In contrast to the FEF, which as we noted earlier has inherently a functional label, the IFJ is instead by its very definition associated with specific sulcal landmarks—namely, the junction of the precentral sulcus (PCS) and the inferior frontal sulcus (IFS), which directly refer to its putative location. In the next section, we then turn to describe and compare the structural properties of the FEF and the IFJ, as well as their relationship to sulcal morphology.

## 2 STRUCTURE

### 2.1 Cytoarchitecture, chemoarchitecture and receptorarchitecture

Traditionally, cytoarchitecture has been the most prominent property used in defining cortical maps (Amunts & Zilles, 2015). However, with the advent of neuroimaging, the balance decisively shifted in favour of non‐invasive in vivo methodologies (Eickhoff, Yeo, & Genon, 2018). In humans, the FEF and the IFJ were indeed initially defined and localized in stereotaxic space thanks to positron emission tomography (PET) and fMRI studies (Derrfuss et al., 2005; Paus, 1996; but see Foerster, 1931, and Penfield & Rasmussen, 1950, for earlier investigations based on electrical stimulation), and only subsequently their specific architectural properties were examined. However, establishing correspondences between cortical borders derived from architectural and functional information has proven challenging. Even if functional borders agree well with architectural ones (e.g., Brodmann areas [BAs]; Brodmann, 1909), some of the latter encompass many functional regions (and vice versa), so any straightforward extrapolation from one map to the other is likely insufficient (Amunts et al., 1999). Moreover, the undue reliance on comparative criteria to examine the architecture of these regions can also be misleading. For example, it could be very well the case that, especially in areas that followed a significant expansion in size in humans compared with other primates (Donahue et al., 2018), areas that underwent evolutionary change also present a different architecture and thus lie within incongruent BA.

These interpretative issues will become immediately apparent when describing the architecture of the FEF. In fact, a substantial impediment in the comparative study of the FEF was caused by the recognition that there are remarkable inconsistencies between the two most widely used cytoarchitectonic maps of the human and of the macaque (Brodmann, 1909; Walker, 1940), particularly in the PFC, in which borders were drawn relying on different cytoarchitectonic criteria, and no explicit comparative considerations were put forward during that process (Petrides et al., 2012). To resolve some of these discrepancies, Petrides and Pandya (1999, 2002) investigated the comparative cytoarchitecture of the dorsolateral PFC and the vlPFC and re‐examined Brodmann's taxonomy in these brain districts. According to the neuroimaging evidence available at the time (in particular, the study by Courtney et al., 1998), the authors suggested that the human FEF is localized within BA8 and BA6, rather than in area 8Ad, in contrast to the macaque. Although the IFJ is not explicitly reported in their second study, we can speculate that this area corresponds to what the authors identify as BA6, BA44 and, to a lesser extent, area BA9/46v. Two subsequent studies have specifically investigated FEF as defined by fMRI evidence and then examined its cytoarchitecture and chemoarchitecture ex vivo. Rosano et al. (2003) investigated the cytoarchitecture and chemoarchitecture of FEF in six subjects. They reported that several chemoarchitectonic features could segregate FEF from rostral regions in the middle frontal gyrus (MFG) and superior frontal gyrus. Their findings indicated that FEF is at a point of transition between the granular and agranular cortices in the vicinity of the superior PCS (sPCS). In a single case ex vivo study, Schmitt et al. (2005) found that cytoarchitecture differentiates two aspects of the putative FEF localized in an area of 2 mm parallel to the lateral convexity and an area of 8 mm in the depth of the PCS. This study also reported that, according to cytoarchitectonic criteria, FEF is for the most part localized in BA6.

On the other hand, the first characterization of the structure of the IFJ derives from a re‐examination of a study by Amunts et al. (1999). In the resulting position paper, Amunts and von Cramon (2006) reported that a sharp change in cytoarchitecture and chemoarchitecture occurs in a region that corresponds to the functionally defined IFJ. The area analysed belongs to BA8, BA6 and BA44. In Amunts et al. (2010), the authors performed the post‐mortem analysis of eight brains, which revealed that the receptor fingerprint of the left IFJ segregates this area from the ventral area 44d based on higher concentrations of AMPA, GABA^A^ and M2 receptor densities (reviewed in Zilles & Amunts, 2018). Interestingly, the authors also reported the segregation of IFJ in two subregions along the banks of the PCS. This distinction parallels evidence from other methodologies (in particular, the studies by Glasser et al., 2016, and Zanto et al., 2010; the latter is discussed in Section 3.3), suggesting the presence of a robust subdivision within the IFJ. Therefore, it would be interesting to assess the agreement between architectural and functional criteria in segregating the IFJ (both from other brain regions and internally) and investigate whether neural populations with different selectivity and function could underlie this subdivision.

In conclusion, based on the available evidence, we can tentatively describe FEF as a region characterized by a dysgranular architecture (i.e., with a weakly developed fourth layer), part of BA6 (and perhaps to a lesser extent, of BA8), and IFJ as a dysgranular region lying in the posterior bank of the IFS, in BA6, BA8 and BA44. However, due to the very limited sample available in post‐mortem studies, the question that is crucially left open is whether these results can be generalized to the localization (and the borders) of FEF and IFJ as defined by non‐invasive methods (primarily fMRI) in larger cohorts. Conversely, it is left open whether evidence derived from probabilistic structural atlases (e.g., Amunts et al., 2020) can be used to independently validate the localization inferred from fMRI experiments.

### 2.2 Localization and relation to sulcal morphology

Traditionally, the FEF and the IFJ have been both localized along the banks of the sPCS and the inferior PCS (iPCS), the former in the vicinity of its intersection with the superior frontal sulcus (SFS), and the latter ventrally, in the vicinity of its intersection with the IFS (see Figure 1a). Due to the absence of compatible sulcal landmarks in monkeys (Donahue et al., 2018), the detailed comparative assessment of similarities in the organization of FEF and IFJ has proven very challenging (but see Neubert et al., 2014; Sallet et al., 2013). It also remains to be elucidated how inter‐individual differences in the sulcal morphology affect the exact localization of FEF and IFJ. Of primary interest in this section are therefore instances of spatial shifts and potential inversions between the respective locations of FEF and IFJ, which would lead to incomplete spatial segregation between these regions when their activity (as measured by fMRI) is pooled at the group level.

The correspondence between the macaque and the human FEF has been a puzzling aspect of FEF localization and function. In the study by Koyama et al. (2004), the authors compared the localization of the macaque and the human FEF to resolve discrepancies in FEF localization, which potentially stemmed from idiosyncratic methodological approaches in the two species. They measured fMRI blood oxygen level dependent (BOLD) activity in both species while performing visually guided saccades in blocked and event‐related designs. They found that the execution of saccadic movements consistently activated similar regions in frontal and parietal cortices. The macaque dorsal lateral intraparietal region corresponded to the human superior parietal lobule (SPL), and the macaque FEF corresponded to the putative human FEF, localized at the intersection of SFS and PCS (BA6). Because activation peaks were also found in premotor regions in macaques (BA6), the authors concluded that the FEF presents a similar anatomical organization across the two species. In the study by Amiez et al. (2006), the authors' goal was to dissociate activity related to manual and saccadic responses, which according to previous neuroimaging evidence often overlapped nearby the junction of the SFS and the PCS. They analysed conditional hand responses and saccadic movements in a blocked‐design fMRI experiment on a subject‐by‐subject basis to clarify the respective activation foci. Their study shows that three main variants can represent the morphology of the junction of the SFS and the PCS and that in all the subjects analysed, the activations associated with the execution of conditional hand movements and saccades tapped into a dorsal and a ventral portion of PCS junction with the SFS, respectively, agreeing with the available comparative evidence in non‐human primates.

Concerning the exact localization of IFJ and its relation to sulcal morphology, the study by Derrfuss et al. (2009) reported that in 13 out of 14 of their participants, the activations found in a task‐switching fMRI study were localized in the iPCS, nearby the junction with the IFS in the left hemisphere. Thus, similarly to the FEF, the localization of the IFJ appears to be tightly associated with the individual sulcal morphology and stable across subjects. Finally, in a study of outstanding interest to the scope of this work by Derrfuss et al. (2012), the authors reported an anatomical and functional double dissociation between the activations elicited by saccades versus button presses in the motor paradigm (in the inferior FEF [iFEF]) and incongruent versus congruent trials in the Stroop paradigm in the IFJ along the banks of the PCS. Of critical importance, the authors performed the analysis of activations at the subject level in the left hemisphere to localize both regions of interest (ROIs). In the case of the iFEF, the contrast between saccades and button presses was aimed at removing two major confounds: (1) this contrast allowed to keep sensory (i.e., auditory) stimulation balanced and to isolate activity specifically related to saccade execution; (2) saccades were executed in darkness to remove any change in visual stimulation during the task. The authors showed that for 16 of the 17 participants, these tasks (viz., the motor and the Stroop task) were successfully able to dissociate activations in the iFEF and the IFJ. Unfortunately, however, the lack of eye‐tracking data is a limitation of this study. For example, previous studies showed that imbalances in blinking behaviour in the absence of visual stimulation can contaminate BOLD signal and significantly alter activity in prefrontal oculomotor sites, such as FEF and the supplementary eye field (Bristow et al., 2005; Hupé et al., 2012). Thus, an alternative interpretation that eye blinks drove iFEF responses cannot be completely ruled out (Kato & Miyauchi, 2003; see also Amiez & Petrides, 2009, for a similar interpretation of iFEF activations).

In summary, sulcal morphology is a fundamental aspect of FEF and IFJ localization and underlies the spatial segregation between these regions. Future studies should investigate the development of the cortical folding in the plPFC and consolidate the brain mapping approaches that allow the dissociation of the activations found along the superior and inferior banks of the PCS.

## 3 FUNCTION

In this section, we turn to describe and compare FEF and IFJ involvement in orchestrating functions and top‐down control, such as visual attention, working memory and executive functions. There are undoubtedly many other aspects that differentiate the role of these areas in cognition. Still, we claim that it is precisely in their differential roles of top‐down guidance that we find the most systematic and prominent differences between these two areas. This work is strongly influenced and inspired by the legacy and pioneering work by Patricia S. Goldman‐Rakic, who carefully studied the organization and the functional specialization of the PFC (Goldman‐Rakic, 1996; Wilson et al., 1993). In the following, we will focus on reviewing more recent human neuroimaging studies of FEF and IFJ activity, mostly in the form of fMRI and MEG, or combinations of the two methods. We choose this focus because the excellent spatial and temporal resolution of these (combined) tools allows measuring and modelling task‐related activities in FEF and IFJ at an unprecedented level of detail, revealing important differences in their involvement in attentional and working memory tasks. Thus, all the remaining comparisons based on different cognitive subdomains and sources of evidence (e.g., lesion studies) are outside the scope of the present work.

To ease the exposition, this section will be organized around the discussion of six well‐known functional dichotomies: modal versus supra‐modal coding, presence versus lack of topographic organization, top‐down versus bottom‐up spatial attention, spatial versus non‐spatial attention, spatial versus object‐based working memory and position within versus outside the multiple‐demand system. Although we acknowledge that some of these dichotomies have been in part superseded (e.g., top‐down vs. bottom‐up attention; Awh et al., 2012; Macaluso & Doricchi, 2013), we will attempt to discuss the evidence on a continuum, thus avoiding unilateral considerations on the role of FEF and IFJ in these dichotomies, and refer to novel theoretical frameworks whenever appropriate. The decision to structure this section in this specific way is essentially motivated by the basic understanding of attention and working memory as fundamental orchestrating functions, which provide the building blocks of other high‐level cognitive functions, such as response inhibition, task‐switching and more generally the hierarchical control of behaviour (Desimone & Duncan, 1995; Fuster, 2001; Goldman‐Rakic, 1996). In accordance with this assumption, the dichotomies discussed also follow a gradual and ordered progression from sensory functions towards more abstract ones (i.e., detached from sensory input). Finally, we would also like to emphasize that the goal of the present review is primarily to facilitate the integration of the role of FEF and IFJ into already existing computational and neural frameworks of attention, working memory and cognitive control (Carrasco, 2011; Desimone & Duncan, 1995; Duncan, 2001; Itti & Koch, 2001; Miller & Cohen, 2001; Serences, 2016; Sreenivasan et al., 2014) by focusing on the implementation of the mechanisms posited by these models at the brain network level (Cole et al., 2013; Corbetta & Shulman, 2002; Fecteau & Munoz, 2006; Fiebelkorn & Kastner, 2020; Koechlin et al., 2003; Nee & D'Esposito, 2016; O'Reilly, 2010).

### 3.1 Sensory domains (modal vs. supra‐modal coding) and topographic organization

Prominent cognitive models posit that the brain areas involved in the selection of competing stimuli and responses need to be supra‐modal in nature (Dux et al., 2006; Norman & Shallice, 1986; Pashler, 1994). However, recent studies using MVPA show that in the plPFC, several areas involved in these processes exhibit selectivity for specific sensory modalities (Michalka et al., 2015; Schwedhelm et al., 2020; Tamber‐Rosenau et al., 2013). In Michalka et al. (2015), the authors used fMRI to investigate the hypothesis that the lateral frontal cortex is organized according to the selectivity to sensory modality by contrasting the activity elicited by a sustained covert visual attention task and an auditory task. In the majority of participants, the contrasts revealed activation foci localized in the sPCS and the iPCS, respectively. There was also a significant gap between the foci in the transverse gyrus of the PCS, where all subjects showed auditory selectivity. Interestingly, there was an additional auditory‐biased region on the caudal portion of the IFS. This study also analysed the intrinsic (i.e., resting‐state) functional connectivity of the previously defined regions with posterior visual and auditory areas. They showed that sPCS and iPCS activity positively correlated with visual attention areas and that activity in the transverse gyrus of the PCS and the caudal IFS correlated with auditory attention areas, whereas the reverse correlation was not found significant or was negative, thus showing the independence of these networks (this original finding was replicated in a larger sample in Tobyne et al., 2017). Finally, in a recent study employing a rule‐based attentional selection paradigm, Germann and Petrides (2020) reported that in the left hemisphere, the ventral part of area 8A is involved in the selection of visual stimuli, whereas in the right hemisphere, its dorsal part is involved in the selection of auditory stimuli, which further supports the idea of sensory selectivity in the plPFC.

In parallel to the previous research line on sensory selectivity in the PFC, studies on the retinotopic organization across the cortex revealed that several foci in the frontal, parietal and occipital cortices show responses to visual stimulation that are organized in a topographic fashion (Benson et al., 2018; Sereno et al., 1995; Wang et al., 2015). These responses are modulated by attention (Saygin & Sereno, 2008) and are hypothesized subserving spatial attention and spatial working memory (Hagler & Sereno, 2006; Jerde et al., 2012; Kastner et al., 2007). In their seminal fMRI study, Mackey et al. (2017) developed a method to reliably elicit activations in frontoparietal regions and computed their population receptive fields (Dumoulin & Wandell, 2008) using a model that accounts for non‐linear responses to stimuli of varying size. In each of the five subjects analysed, the authors identified two topographic maps in the sPCS and the iPCS. Both these cortical patches contained a representation of polar angle and eccentricity. In the sPCS, the foveal representation was localized at the junction of the PCS with the SFS, which allowed the separation of this region in two visual field maps (referred to as sPCS1 and sPCS2 below) containing a complete representation of contralateral space. In contrast, the organization of the iPCS was less clear, but this area seemed to be still capable of representing eccentricity and the contralateral space in a systematic way. The authors related these newly defined maps to the topographic atlas by Wang et al. (2015) and the multimodal parcellation 1.0 (MMP1) by Glasser et al. (2016). The sPCS2 corresponded to the FEF in both of those atlases, whereas the sPCS1 corresponded in large parts to the more dorsomedial areas 6a and 6d in the MMP1. The iPCS corresponded to the ventrolateral premotor eye field and the posterior IFJ (IFJp) regions, and area 6r in the MMP1, but this correspondence varied considerably across subjects and between hemispheres. Although the sample size of this study was quite small, and thus the topographic mapping technique that the authors developed requires to be validated in a larger sample, this study suggests that FEF can confidently be included in the set of topographic visual areas. The evidence for a topographic organization of IFJ is instead comparatively weaker. Indeed, if IFJ may to some extent represent eccentricity, its overlap with activations found in the iPCS was very loose. Furthermore, evidence in non‐human primates indicates that neurons in the vlPFC (specifically, in the ventral pre‐arcuate region, the putative homologue of the human IFJ; Bichot et al., 2015, 2019) have extensive receptive fields that can sometimes extend to the ipsilateral space—a fact that again suggests little evidence of topographic organization (Bichot et al., 2015, 2019).

In summary, although it has been recently suggested that FEF and IFJ are both primarily unimodal (visual) areas, we reviewed here novel evidence that favours a more general involvement of the FEF in spatial tasks, as only FEF contains a precise topographic representation of contralateral space, whereas the IFJ (and in particular, the IFJp as defined according to the MMP1) has only quite limited overlap with topographically organized areas (as the iPCS activations found by Michalka et al., 2015, and Tobyne et al., 2017; see also Mackey et al., 2017). Further work will be required to characterize the organization of topographic maps in the plPFC and their correspondence with the parcellation schemes derived from multimodal MRI data (Glasser et al., 2016; Neubert et al., 2014; Sallet et al., 2013).

### 3.2 Top‐down versus bottom‐up spatial attention

Much of the early neuroimaging studies (PET and fMRI) on the brain regions and networks subserving visual attention was dedicated to understanding how the brain achieves top‐down control over visual input or, conversely, how attention is automatically driven by bottom‐up factors (Corbetta & Shulman, 2002). These pioneering studies coalesced in the seminal proposal that a dorsal attention network (DAN; with FEF and the intraparietal sulcus [IPS] as primary nodes) controls top‐down visual selection. Although it is not explicitly mentioned, also parts of the IFJ seem to be localized within this network, with MFG as the most closely labelled brain structure. In contrast, a right‐lateralized ventral attention network (VAN), with inferior frontal gyrus (IFG) and the temporoparietal junction (TPJ) as primary nodes, was hypothesized to be involved in the automatic reorienting of attention to external (salient) stimulation (Corbetta & Shulman, 2002). Although this proposal was very elegant in the way it reconciled the neuroimaging evidence available at the time, and it was successfully able to isolate the main nodes of the attention networks that were involved in a variety of experimental tasks (reviewed in Fiebelkorn & Kastner, 2020), and also in spontaneous, resting‐state activity (Fox et al., 2006), it suffered from several limitations. First of all, earlier studies failed to appreciate the distinction between reorienting to predictable versus unpredictable stimuli (Shulman et al., 2009), and secondly, between reorienting of attention and the evaluation of the appropriate stimulus‐response mapping, mainly due to the lack of time resolution (Han & Marois, 2014). Finally, these initial studies were not designed to model directed interactions between DAN and VAN nodes. Vossel et al. (2012) addressed the latter question in an fMRI study by analysing the effective connectivity between FEF, IPS, and the right TPJ and IFG (likely also comprising the right IFJ) using dynamic causal modelling. In their spatial task, the participants were instructed to discriminate an orientation grating shown at the right or the left of the fixation, and the predictability of the spatial cue was manipulated between blocks (90% vs. 60%; the subjects were made aware of the manipulation by the colour of the cue). The author contrasted valid > baseline and invalid > valid trials to extract the DAN (FEF, IPS) and VAN (IFG, TPJ) nodes, respectively, at the single‐subject level in a whole‐brain analysis. They then separately evaluated a wide array of competing models of modulatory effects within the DAN and VAN nodes, and from/to the visual cortex, employing a random effect Bayesian model. This analysis revealed that, in the DAN, the model with the highest empirical support consisted of bilateral connections between FEF and IPS of both hemispheres, and between the right and left FEF. In the VAN, a model with bilateral connections from the visual cortex to the right TPJ and from there to the right IPS and IFG was best supported by the data. They also tested the effect of validity and cue predictivity in the modelled VAN connections and showed that in all areas, activity was increased in invalid, high cue predictivity trials. Moreover, the connection between right TPJ and right IPS was enhanced in this condition, indicating a potential communication mechanism between the VAN and DAN, suggesting that the VAN is right lateralized.

Within a similar theoretical framework, the fMRI study by Wen et al. (2012) tested the interactions between the DAN and VAN in a spatial attention task using Granger causality, and the functional role of these two networks for behaviour. Their task consisted of a spatial cue followed by a 2.5‐s delay, after which the subjects were required to respond to an unlikely target (20% of the trials) in the valid condition and to ignore the stimulus in the unattended hemifield. The authors first contrasted attention blocks with passive‐view blocks to isolate the main DAN and VAN nodes, which comprised the bilateral FEF and IPS, and the right anterior MFG, the right posterior MFG and the right TPJ (and also additionally, the bilateral dorsal anterior cingulate cortex and anterior insula), respectively. After computing Granger causality between all ROI pairs, they sorted their behavioural results (reaction time [RT] and accuracy) in bins and correlated the binned values with the Granger‐causal values. The results suggest that Granger‐causal influences from the rIPS to the rTPJ are associated with increased accuracy, whereas the reverse influences from rTPJ to rIPS are detrimental to behavioural performance (both in RT and in accuracy). Moreover, by aggregating data from all the DAN and VAN ROIs, the authors showed that Granger‐causal influences from the DAN to the VAN were again associated with increased accuracy, whereas influences from the VAN to the DAN led to worse RT, which further supported the results from the single ROI analyses. Interestingly, however, they also noted that the right posterior MFG (which seems to match quite well the location of the IFJ according to the sulcal landmarks) had a more ambiguous relationship with the DAN because it had directional influences on the rFEF, lIPS and rTPJ, all of which correlated positively with enhanced performance. Overall, the authors interpreted these findings as consistent with a model in which the DAN exerts top‐down control and suppresses VAN activity to achieve efficient filtering of distracting stimuli, whereas the VAN is mainly involved in breaking the attentional set.

As the role of the FEF in top‐down attention was already well established by both comparative and fMRI evidence (Buschman & Miller, 2007, 2009; Corbetta, 1998; Corbetta & Shulman, 2002; Gregoriou et al., 2009; Kastner & Ungerleider, 2001; Moore & Armstrong, 2003), one of the aims of the following research was to understand the contributions of the IFJ to the DAN and VAN. Asplund et al. (2010) investigated the effects of the interaction between goal‐driven and stimulus‐driven factors on fMRI activity during the performance of a non‐spatial (i.e., centrally presented) rapid serial visual presentation task (RSVP). Unbeknownst to the participants, they inserted a human face in a small percentage of trials that acted as an irrelevant salient stimulus and caused a marked decrease in target detection (they refer to this effect as surprise‐induced blindness). Their results show that, in the first two presentations of the distractor, there was an increase in activity in the IFJ and TPJ, after which neural responses adapted to the distractor, and that this modulation matched the behavioural performance in surprise trials. The authors also found evidence of increased activation in the FEF and IPS, although at longer delays (3 s), which therefore was not affecting behavioural performance. Thus, their study suggests the idea that the IFJ may act as an interface between the stimulus‐driven VAN and the goal‐driven DAN and that it could mediate the conscious perception of behaviourally relevant stimuli. Han and Marois (2014) attempted to dissociate the activity related to transient reorienting from sustained activity related to the evaluation of the stimulus in an Rapid Serial Visual Presentation (RSVP) task contrasting short versus long (1 and 10 s) ‘oddball’ trials. The results of their experiments (fMRI exp. 1–2) revealed that the TPJ and IFJ showed both transient and sustained responses to salient stimuli, whereas several other areas in the PFC (e.g., the insula and the cingulate cortex) had transient responses only, a result that implicates these regions in ongoing evaluative processes. Finally, in their fMRI study, Tamber‐Rosenau et al. (2018) investigated how the DAN and VAN contribute to transient and sustained attentional processes. They used an endogenous spatial cueing paradigm with variable delays in target presentation to specifically tease apart the role of the IFJ in the configuration and maintenance of attentional priority. They found that in the right hemisphere, the FEF and IPS showed evidence of both transient (i.e., at the end of the cue and the target presentation) and sustained activities (i.e., during the whole delay period). In contrast, the bilateral IFJ showed only evidence of transient activity, pointing to an initial involvement of this region in the configuration of the attentional state. We note that a potential reason for the discrepancy with the results reported by Han and Marois (2014) is that in the latter study, the authors focused on analysing only longer delay trials (9, 13 and 15 s) as their original goal was to dissociate the IFJ from DAN nodes (i.e., the FEF and IPS), and therefore, they defined sustained activity as one that is significantly elevated relative to baseline during the entire delay period. Accordingly, although the right IFJ showed evidence of sustained activity if delay periods were pooled together, this was no longer the case when the results were further analysed separately for each delay period. Concerning the DAN nodes, their results are consistent with models of a priority map within the FEF (similar to priority maps in the posterior parietal cortex; see Fecteau & Munoz, 2006; Itti & Koch, 2001; Thompson & Bichot, 2005; Zelinsky & Bisley, 2015). The function of the frontal priority map is to integrate bottom‐up and top‐down signals and to guide perceptual and action selection (Fecteau & Munoz, 2006).

However, there are still aspects of the role of the FEF and IFJ in top‐down selection that remain quite elusive. In macaques, the latency of FEF responses has been estimated to be approximately 40 ms, with some neurons exhibiting latencies comparable with V1 and V2 neurons (Bullier, 2001). In a recent electrocorticographic study, Martin et al. (2019) found comparable latencies in the human FEF in response to a spatial cue (62 ± 5 ms), which were again in the order of early visual cortex latencies. However, surprisingly, they reported that the effects of attentional modulation were more pronounced, and their latencies were shorter, in extrastriate areas and the parietal cortex, compared with frontal and visual cortices. These results highlight the need of pursuing similar research efforts to successfully bridge electrophysiological research in non‐human primates and humans. To summarize, whereas the FEF is consistently involved in top‐down spatial selection and may provide the instantiation of a priority map where top‐down signals are integrated with bottom‐up activity and maintained, the IFJ acts as a flexible hub that toggles between the DAN and VAN activities and modulates activity in the DAN according to the current behavioural demands and context.

Finally, although the in‐depth discussion of this subject is partly outside the scope of the present work and would certainly deserve to be reviewed separately, here, we would like to describe and point the reader to outstanding transcranial magnetic stimulation (TMS) evidence that helps to reveal the contribution of the FEF and IFJ to attentional control and working memory processes, thus complementing the correlational studies described earlier with causal evidence. In Ruff et al. (2006), the authors combined fMRI with concurrent TMS and revealed that repetitive TMS over the right FEF increases the BOLD signal in peripheral locations and reduces it in a central location in retinotopic regions (V1–V4) depending on the TMS intensity in an additive way, both in the presence and absence of visual stimulation. This effect was shown to be behaviourally relevant by a follow‐up psychophysical experiment, in which TMS over the same site (rFEF) enhanced the perceived contrast of a peripheral Gabor patch in both visual hemifields. Subsequent studies were able to replicate and extend this set of findings to a variety of visual tasks (see, e.g., Bardi et al., 2012; Neggers et al., 2007; Ronconi et al., 2014; Taylor et al., 2007; Vernet et al., 2014, for a review). In their innovative multimodal study, Marshall et al. (2015) further uncovered the neural mechanisms that underlie FEF influences over occipital sites. They first localized FEF sites at the individual subject level in a preliminary fMRI session. They subsequently administered continuous theta burst stimulation (cTBS) with TMS to the left and right FEF, and a third control site (vertex), and recorded the neural activity in the 30 min afterward using MEG while the participants were performing a spatial attention task. Their results show that cTBS over the rFEF (co‐registered to the T1‐w image) impaired performance for contralateral stimuli, decreased anticipatory alpha power modulation in the contralateral hemisphere and increased gamma power in the left hemisphere after rFEF stimulation. Together, these findings highlight the oscillatory signatures of the top‐down control exerted by the FEF over the occipitoparietal cortex and potentially suggest a role for interhemispheric interactions. The role of the FEF in top‐down control was further supported by a recently published study by Veniero et al. (2021), which combined online TMS with electroencephalography (EEG). In their first experiment, the authors applied single‐pulse TMS to the rFEF and showed that this pulse led to an oscillatory alignment in the beta frequency band as measured from occipital channels. In a second experiment, they applied single‐pulse TMS to the rFEF while the participants were performing a motion discrimination task. They showed once again that this pulse led to an oscillatory alignment and, more importantly, that this modulation caused an analogous cyclic effect on visual performance in the same beta band. In two follow‐up experiments, these effects on visual perception were confirmed via dual‐site TMS on the rFEF, on the one hand, and on rV5/rV1, on the other, by examining phosphene perception. This analysis revealed a similar rhythmic modulation as in the previous experiments of rV5 excitability, but not rV1, thus providing anatomical specificity to the results of their study.

The TMS evidence on the IFJ is much scanter, but nevertheless essential to understand its role in top‐down control. The influential study by Zanto et al. (2011) used a non‐spatial, feature‐based task where subjects were asked to either remember colour information and ignore motion direction or vice versa, and respond to a probe item (see Zanto et al., 2010, for more details on the methodology; described in the following section). They applied repetitive 1‐Hz TMS on the right IFJ (defined in a separate fMRI experiment) for 10 min and measured EEG while the participants were performing their behavioural task. They showed that in the first half of the experiment, repetitive TMS decreased behavioural accuracy and the amplitude of the P1 component in the ‘attend colour’ condition. These results were further confirmed by analysing the phase coherence between frontal and posterior electrodes in the alpha band, which was previously identified as a signature of neural modulation, and that decreased following repetitive TMS. In the study by Muhle‐Karbe et al. (2014), the authors investigated the role of the left IFJ and left IPS in task preparation. These nodes were defined by performing an fMRI meta‐analysis of task‐switching studies (45 studies) using the activation likelihood estimation (ALE) technique (Eickhoff et al., 2012) and targeted them by repetitive TMS during the execution of a cued task‐switching paradigm. This paradigm contained blocks in which the task goal was repeated, but subjects had to change their response set, and blocks in which the response set was constant, but they had to reconfigure their task goal. Crucially, by delivering repetitive TMS on these sites before the target onset, the authors revealed a double dissociation between the lIFJ and lIPS in task preparation; namely, that the stimulation of the former impaired the reconfiguration of task goals, but not of the response set, and conversely, that the stimulation of the latter impaired the updating of the response set, but not of the task goal. Finally, in a third experiment, the authors showed that the lIPS was also implicated in reconfiguring the task goal, but at a later stage in the information processing cascade. These results therefore highlight the lIFJ as one of the sources of the top‐down signals to posterior parietal areas that it is crucial to cognitive control functions (for converging evidence, see Hippmann et al., 2019; Muhle‐Karbe et al., 2018; Verbruggen et al., 2010). In addition, as in both the study by Zanto et al. (2011) and Muhle‐Karbe et al. (2014), the task‐relevant dimension was feature based (colour/motion and shape/colour, respectively), this suggests the intriguing possibility that the IFJ may be specifically involved in encoding and manipulating information in a non‐spatial (feature‐ and object‐based) representational format. We will elaborate more in detail on this suggestion in the general discussion, where we will also provide a concrete example of how TMS could be used to directly address this open question. Thus, now we will move on to discuss more broadly the relationship of the FEF and IFJ to spatial and non‐spatial selective mechanisms.

### 3.3 Spatial versus non‐spatial attention and spatial versus object working memory

**FIGURE 2:**
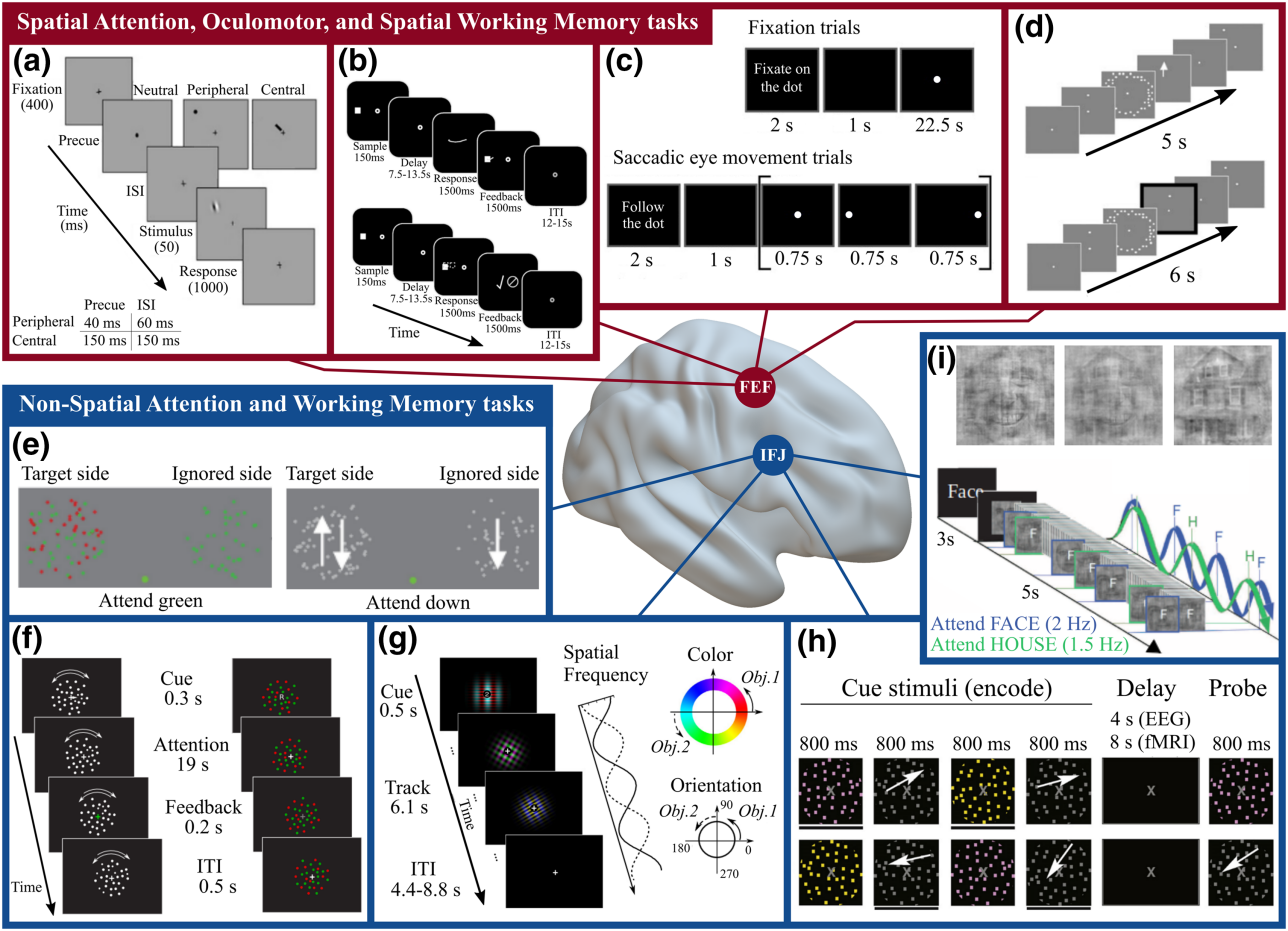
Paradigms that allow disentangling spatial and non‐spatial attentional and working memory mechanisms. Red outline: Paradigms that involve a strong spatial component. The fundamental basis of all the tasks shown in this group is represented by the spatial cueing paradigm (a) (adapted from Carrasco, 2011). (b) The tasks from the study by Srimal and Curtis (2008), consisting of a memory‐guided saccade (top) and a spatial item recognition task (bottom). (c) The saccadic eye‐movement paradigm from the study by Amiez et al. (2006; adapted from Amiez & Petrides, 2009). Top row: Control task, in which subjects keep fixation. Bottom row: Experimental task, in which subjects need to perform a sequence of visually guided saccades. This paradigm represents the classical FEF fMRI localizer. (d) The memory‐guided saccade task from the study by Kastner et al. (2007). This study provided compelling evidence of the presence of topographic maps in the PFC (and crucially, in the sPCS/SFS, encompassing the FEF). Blue outline: Paradigms that involve purely non‐spatial mechanisms. (e and f) The feature‐based attention tasks from the studies by Zhang et al. (2018) and Liu et al. (2011). In these tasks, the subjects are asked to hold fixation and are instructed by an endogenous cue to pay attention to the features of a stimulus (either colour or motion) and to detect a sudden change in luminance of a dot or an increase in its speed of motion. Because this imperative event can happen randomly in each portion of the cloud‐like stimuli, spatial information is rendered ineffective for solving the task at hand (in the study by Zhang and colleagues, half of the dots were replaced and reappeared at new locations each 100 ms to discourage even more the use of spatial strategies; the attended and ignored sides were blocked). (g) The purely object‐based attention task devised by Liu (2016). The colour, orientation and spatial frequency of the two superimposed Gabor patches changed simultaneously over the trial, so the subjects needed to pay attention to their identity to effectively perform the task. (h and i) The working memory task used by Zanto et al. (2010) and the object‐based attention task from the study by Baldauf and Desimone (2014). All these feature‐ and object‐based attention and WM tasks engage the IFJ, which is responsible for modulating activity in downstream visual areas (e.g., MT+, V4, PPA and FFA) to enhance behavioural performance (Baldauf & Desimone, 2014; Zhang et al., 2018)

Functionally, it is very well possible to dissociate the behavioural effects and the neural mechanisms of spatial versus feature‐ and object‐based (i.e., non‐spatial) attention (Carrasco, 2011; Desimone & Duncan, 1995; examples of the classic spatial and non‐spatial cueing paradigms are shown in Figure 2). Indeed, whereas spatial attention enhances the neural activity that codes the location of the attended stimulus (Kastner & Ungerleider, 2001), non‐spatial attention acts globally on the entire visual scene (Baldauf & Desimone, 2014; Serences & Boynton, 2007; Störmer & Alvarez, 2014) and boosts the signals that code for the attended feature or object (i.e., through the ‘attentional template’; Desimone & Duncan, 1995), while also suppressing irrelevant, distracting information (Gaspelin & Luck, 2018). As we have seen, in the plPFC, some areas adjacent to and including the FEF possess a topographic organization (reviewed in Section 3.1), thus being well adept to provide top‐down location‐specific modulation of neural activity. In contrast, another set of areas have extensive receptive fields (Bichot et al., 2015, 2019), which often encompass also the ipsilateral hemifield, which may account for the global nature of their modulation on visual processing. Because the attentional template needs to be held active during the task, it has been proposed that sustained, top‐down attention (both spatial and non‐spatial) relies on the same neural machinery as working memory processes, both in terms of their cognitive routines and the brain networks involved (Chun et al., 2011; Gazzaley & Nobre, 2012; Zelinsky & Bisley, 2015). It is widely agreed that perceptual attention and working memory are intimately linked (Awh & Jonides, 2001; Baddeley, 1993; Olivers, 2008). Not only is it a crucial component of top‐down perceptual attention to keep templates for targets of the perceptual selection internally sustained over time (e.g., Awh et al., 1998; Chun et al., 2011; LaRocque et al., 2013; Oberauer, 2019). In this sense, Kiyonaga and Egner (2013) described the two components in an integrative account as internal versus external selection processes, respectively. Experimentally, also many forms of interactions have been described between working memory and attention (Olivers, 2008). For example, Kim et al. (2005) showed that increased ongoing working memory load reduces perceptual distraction. Similarly, eye movements systematically interfere not only with attention but also with working memory (Lawrence et al., 2004). Further, Lepsien et al. (2011) showed that voluntary attention shifts modulate the maintenance of working memory. Finally, Mendoza‐Halliday and Martinez‐Trujillo (2017) demonstrated that in the lateral PFC of the macaque, identical neuronal populations encode both perceived and memorized visual features. Generally, when discussing attention control, it is worth emphasizing that an attentional cue can be given with or without stimuli present in the environment (see, e.g., Figure 2a–c,e–h): if there are stimuli in the environment directly following the cue (e.g., moving dots), it is more complicated to determine experimentally to which extent the neural activity in the cue‐target interval reflects top‐down control signals as opposed to being evoked by sensory input itself. In this section, we therefore build upon the hypothesis that both attention and working memory functions are underlaid by overlapping prefrontal activity. We will thus review in a unitary framework the evidence on how spatial and non‐spatial processes are mediated by the FEF and the IFJ, respectively.

Inspired by studies on non‐human primates and the pioneering work of Goldman‐Rakic and colleagues (Goldman‐Rakic, 1996; Scalaidhe et al., 1997, 1999; Wilson et al., 1993), researchers originally investigated the segregation of spatial and object working memory circuits in the human plPFC, but the evidence in support of this claim was mixed (Rao et al., 1997; Wager & Smith, 2003, for a landmark meta‐analysis of early neuroimaging studies). Courtney et al. (1998) showed that a distinct cluster in the SFS (localized anteriorly to the FEF) was predominantly involved in spatial working memory, rather than working memory for object classes, such as faces. However, their interpretation was challenged by Owen et al. (1998). They hypothesized that the difference in the involvement of dorsal and ventral prefrontal regions was not due to the information content being encoded by these regions (i.e., location vs. object information) but rather the result of dissociation in other underlying cognitive processes. In particular, in their study, they showed that, when monitoring requirements were precisely matched between the spatial working memory and the non‐spatial working memory task, both tasks elicited overlapping activations in the mid‐dorsolateral PFC (BA46 and BA9/46). Despite these contrasting results, the dispute was far from settled and was from then on centred around how the plPFC acts as a gateway to establish priority on sensory inputs to implement goal‐driven behaviour, and the nature of its selectivity to sensory information as opposed to the types of cognitive processes, the task's executive demands or practice with it (Constantinidis & Qi, 2018; O'Reilly, 2010; Wager & Smith, 2003). More recently, thanks to the improvements in fMRI data acquisition, and the availability of more sophisticated analytic tools (MVPA, and meta‐analytic modelling techniques), the hypothesis of dorsoventral segregation based on the selectivity of plPFC neurons for spatial versus non‐spatial informational content regained considerable interest in the field (see, e.g., the meta‐analysis by Rottschy et al., 2012, for the dissociation of working memory activation foci related to object identity and location in the plPFC).

The initial neuroimaging evidence on the spatial selectivity in the FEF was strongly tied to studies investigating the overlapping mechanisms of covert and overt (i.e., via eye movements) visual selection (Deubel & Schneider, 1996; Hoffman & Subramaniam, 1995; Kowler et al., 1995; Moore & Armstrong, 2003; Posner, 1980). Influenced by the premotor theory of attention (Rizzolatti et al., 1987), these studies predicted that the same brain regions involved in the planning and the execution of saccadic eye movements should also mediate covert shifts of attention (Corbetta, 1998). Several independent groups found that covert and overt spatial attention tasks consistently activated overlapping networks that included the FEF. This lent indirect support to the hypothesis of a premotor origin of covert spatial attention signals (Corbetta et al., 1998; Fiebelkorn & Kastner, 2020; Moore & Fallah, 2001; but see Thompson et al., 1997; for evidence of a link between microsaccades and covert attention, see Lowet et al., 2018). Although following research has to some extent challenged this view, for example, highlighting that in the FEF not all neurons have visuomotor and motor functions (Schall, 2015), implying that covert and overt signals might originate from different neural populations, these studies were pivotal in establishing the FEF as a core region for spatial attention. The idea of spatial selectivity in FEF neural populations was further strengthened by the observation of topographically organized maps in this region (reviewed in Section 3.1), which encoded the attended location in a priority map via sustained activity during the delay period of attention and working memory paradigms (Awh & Jonides, 2001). Consistent with this view, a groundbreaking study by Jerde et al. (2012) showed that in the sPCS (likely, within the FEF) and IPS2, decoding techniques could cross‐predict BOLD activity in the spatial tasks the subjects were performing (i.e., covert attention, working memory or overt saccadic motor task; Figure 2c). Their MVPA classifier was separately trained on each task's data and predicted activation patterns in the two left‐out tasks, again showing that the networks involved in these functions share a significant degree of overlap in the close vicinity of the FEF. Even more strikingly, this activity was shown to be tied to the stimulus location (because the ROIs were extracted using retinotopic mapping), thus providing a very elegant demonstration that these areas are primarily involved in visuospatial selection, and agnostic to the behavioural response required (as one would assume if the FEF and IPS worked as spatial priority maps). One critical aspect in understanding the organization of the FEF is whether the tightly coupled functions of spatial attention, oculomotor control and spatial working memory are all implemented strictly inside the FEF, or involve also other adjacent brain areas, and whether they can be assigned to one or multiple subdivisions of the FEF. Previous studies were not able to exactly clarify the relationship between spatial attention and spatial working memory within the FEF, mostly owing to the fact that topographic mapping techniques have not been yet standardized for prefrontal regions and that previous studies often used hybrid (i.e., spatial and non‐spatial) visual stimulation paradigms to elicit activations in the FEF (Hagler & Sereno, 2006; Mackey et al., 2017; Saygin & Sereno, 2008). In the rhesus macaque, the area involved in spatial working memory lies immediately anterior to the FEF (BA46; Funahashi et al., 1989; Fuster & Alexander, 1971), but the often incompatible arrangements of prefrontal areas and inconsistencies in BA labelling prevent a straightforward translation of this anatomical evidence from this species to humans (as previously discussed in Section 2.1). Kastner et al. (2007) employed four purely spatial tasks to examine the relationship between spatial working memory, spatial attention and oculomotor control in the FEF and to enable a more direct comparison with the typical working memory paradigms used in the non‐human primate literature (Figure 2d). Their study showed considerable overlap between the activations in the memory‐guided saccade task and the spatial working memory tasks in the sPCS/SFS (i.e., the FEF) and in the iPCS/IFS. The authors interpreted this finding proposing that the iPCS/IFS region should be considered the human homologue of the region specialized for spatial working memory in monkeys, which is not however fully consistent with other comparative evidence (Constantinidis & Qi, 2018). Nevertheless, this study was crucial in establishing the correspondence of spatial attention and working memory maps in the plPFC (see also Srimal & Curtis, 2008, for correspondences within the FEF; the tasks employed in the latter study are reported in Figure 2b).

Regarding the hypothesis of feature and object selectivity in the vlPFC, early neuroimaging studies on working memory (Owen et al., 1998; Wager & Smith, 2003) may have failed to observe evidence in favour of it for a simple and yet crucial experimental factor. When a stimulus is presented in the periphery, its features and identity likely need to be bound to its actual location in the visual field, but this process is a by‐product of the spatial arrangement of the stimuli, and not universally valid for visual features that are not bound to a specific location in space (e.g., if they are presented holistically over the complete visual field). Although in everyday life visual features are often bound to specific locations in the periphery, a much more stringent way to measure pure feature‐ and object‐based mechanisms is only achieved in experimental settings where no spatial information can be used to solve the task at hand. Several paradigms meet this fundamental requirement, the most common ones involving tasks in which the subjects are instructed to maintain fixation and to perform the visual detection/discrimination of a target appearing in several superimposed, spatially overlapping stimuli (Baldauf & Desimone, 2014; Carrasco, 2011; Serences et al., 2004).

Another potential methodological issue of previous studies was that spatial and non‐spatial cues were often presented within the same experimental block (see, e.g., Giesbrecht et al., 2003), and this could have potentially biased the subjects' strategies (Slagter et al., 2007). In the study by Greenberg et al. (2010), the authors investigated whether voluntary shifts of attention towards a location or a feature (i.e., colour) engaged a common source in the attention networks. The participants were instructed to covertly attend one of four clouds of moving dots, one red and one green dot cloud overlapping in the left and right hemifield, respectively, and to either hold attention at the selected location and switch colour (when the dots in the clouds moved downwards) or hold colour and switch location (when the dots moved upwards). The results of their univariate analysis show that the bilateral mSPL, FEF/supplementary eye field and left MFG/IFG all showed transient responses to shifts of attention to both location and colour time locked to the cue. Interestingly, MVPA revealed above‐chance accuracy in the mSPL for location and colour shifts, indicating that this region may host neural populations that are tuned to spatial and feature information. Although these results importantly suggest that several regions in the PFC may act in coordination to perform shifts of spatial and feature‐based attention, they do not reveal the temporal structure and the interplay between those processes, as the experimental set‐up used visual features in the periphery, mixing spatial and non‐spatial selection processes. Liu et al. (2011), on the contrary, targeted purely feature‐based attentional mechanisms in an fMRI study. The authors conducted two experiments in which the subjects were asked to attend to motion and colour information to detect random increases in dot motion velocity and luminance, respectively, on stimuli superimposed at the central fixation (Figure 2f). In their deconvolution univariate analysis, they identified four regions in which activity was sustained above baseline during both tasks: the aIPS, FEF, vPCS and the medial superior frontal gyrus. MVPA revealed that signals related to colour and motion could be classified above chance in all these ROIs, including the FEF. Thus, the authors interpreted these findings by proposing that PFC may represent attentional priority, regardless of the type of attended feature. Unfortunately, however, overt eye movement behaviour was not controlled inside the scanner leaving the possibility that systematic eye movement patterns contributed to the signal decoded from the FEF.

Using similar stimuli with centrally presented dot clouds, Zanto et al. (2010) combined fMRI and EEG to investigate the top‐down modulation of feature processing during working memory encoding. In their task, participants viewed four sequential frames of overlapping dots and had to remember either their colour or motion direction and ignore the opposite dimension to respond to a probe stimulus (Figure 2h). In the fMRI experiments, areas V4 and V5/hMT+ (selective for colour and motion information, respectively) were localized at the subject level using a one‐back working memory task. Seed‐based functional connectivity of these regions was then used to identify the regions that exhibited increased connectivity in the attend colour versus ignore motion (and vice versa) condition of the experiment. This analysis revealed that only the IFJ was involved in the modulation of both V4 and V5/hMT+ activities and that the putative sources of this modulation formed part of separate subregions in the former. EEG source localization was used to analyse the temporal profile of activity corresponding to the right IFJ and three posterior sites because V4 activity modulation correlated with increased V4‐to‐right IFJ functional connectivity. Phase‐locking values between the rIFJ and central electrodes in the 70‐ to 200‐ms window of interest in the alpha frequency band were significantly greater when recall performance was above the median in the colour task. This analysis of coherence suggests that the alpha band might reflect top‐down modulation of colour processing. The authors also highlighted that the rIFJ subregion involved in motion processing is more dorsal, whereas the colour subregion is more ventral, thus providing evidence that the segregation of the visual streams extends up to the plPFC within the IFJ.

In the study by Sneve et al. (2013), evidence of the transient role of the IFJ was found during the performance of a working memory task requiring delayed orientation discrimination. In particular, this study found increased bilateral activity in the IFJ during the encoding process compared with a control task where no working memory encoding was required and instead the orientation discrimination was performed immediately. This result was complemented by significantly elevated activity relative to baseline during the delay period in the FEF and aIPS, but no evidence of this pattern in the IFJ, implicating the former regions in working memory maintenance. Finally, the authors also used Granger causality to model the effective connectivity between the IFJ, FEF and aIPS. They showed that IFJ activity during working memory encoding predicts activity in the FEF and aIPS during the maintenance phase and therefore suggested that the IFJ is involved in sending top‐down signals to these regions to initiate working memory maintenance processes. Despite being limited by some of the intrinsic weaknesses of the Granger causality analysis (Seth et al., 2015), this study was to the best of our knowledge the first that using this technique reported directed interactions between the IFJ and FEF during the performance of a feature‐based working memory task. Finally, Zhang et al. (2018) performed two fMRI experiments using feature‐based attention tasks on colour and motion stimuli to investigate the source(s) of the spatially global effect of feature‐based attention on visual activity (shown in Figure 2e). In their paradigm, subjects were cued to attend either the colour (red/green) or the motion (upward/downward) of a group of dots displayed in the left or the right visual hemifield, while ignoring the dots on the other (which could share the attended feature or not). The authors also ran two localizer tasks at the beginning of each experiment and used a general linear model to identify the ROIs activated by the stimulus type (V4 for colour features and MT+ for motion features) and by the blocked experimental task (feature‐based task vs. control task, activating the IPS, FEF, IFG and the medial frontal gyrus). They showed that V4 and MT+ activity was significantly elevated when they presented a matching feature to the one attended in the to‐be‐ignored visual hemifield compared with a mismatching one. This result thus replicated the classic global effect of feature‐based attention (Serences & Boynton, 2007). Then, the authors used dynamic causal modelling to evaluate 15 different models that could potentially account for feature‐based modulatory effects on neural activity in the ‘same’ condition in each participant. By comparing the exceedance probabilities (computed using a Bayesian hierarchical approach) of each model, they revealed that a model with feedback modulation from IFJ to V4 and MT+ (which interestingly was also enhanced in the contralateral compared with the ipsilateral hemisphere) was better able to account for the results of both Experiments 1 and 2, respectively. A converging interpretation was also suggested by the fact that the attention modulation index correlated significantly with the effective connectivity strengths from the IFJ. In an additional control analysis that also used dynamic causal modelling, by evaluating several families of models, the authors showed that signals recorded in the IPS, FEF, IFG and the medial frontal gyrus were also driven by the modulation from the IFJ, thus excluding the possibility that the effects on V4 and MT+ could be due to a third node of the network. They complemented this analysis by Granger causality measures, which in contrast to dynamic causal modelling (that requires the specification of a priori ROIs), can be used as a fully data‐driven technique. This showed that the IFJ was the node with the greatest outflow and netflow degree, whereas V4 and MT+ were the nodes with the greatest inflow. In summary, by combining the accurate localization of the ROIs with the analysis of their effective connectivity, this study presents compelling evidence that the IFJ can be considered as the source of the global effect of feature‐based attention.

Taken together, the studies reviewed thus far point to an involvement of the IFJ in both feature‐based attention and working memory, but can the IFJ be considered part of a purely non‐spatial system? For this to be the case, evidence that attentional selection in the IFJ operates at the level of whole objects (i.e., as object‐based attention) is crucial. As we have seen, disentangling non‐spatial processes at the behavioural level requires sophisticated experimental techniques and designs that render spatial information irrelevant during the execution of the task. Furthermore, studying object‐based control processes requires also ruling out the possibility that the participants could rely on low‐level features to perform the task. In this sense, the study by Liu (2016) was designed to explicitly test and isolate neural processes related to purely object‐based attentional selection. In the experimental paradigm of this study, participants were presented with superimposed visual stimuli (Gabor patches) that simultaneously changed in spatial frequency, orientation and colour, thus making both spatial‐ and feature‐based selection ineffective for task completion (see Figure 2g). At the beginning of each trial, one of the patches was cued, and the subjects had to track it to perform a change detection task. By using MVPA on the trials in which the subjects did not make a response (to exclude motor and decision‐making components), the study showed that the attended object could be decoded above chance in the bilateral IFJ, FEF and aIPS, providing evidence of purely object‐based priority signals in these regions.

Critically, even though all the regions reported in most of the previous studies (i.e., the IFJ, FEF and IPS) are implicated in top‐down modulation, measuring the temporal profile of their involvement in spatial and non‐spatial processes is crucial to firmly establish which are the sources of the modulation within the attention networks. In contrast to fMRI, MEG is ideally suited to measure neural activity with a high temporal resolution and to reveal the statistical interdependence of signals originating from different cortical sources (see Gross, 2019, for a recent discussion of MEG unique strengths). Furthermore, combining the spatial accuracy afforded by fMRI localization techniques with MEG by reconstructing a realistic head model of the subjects, and mapping the magnetic signals to the source space by computing the inverse solution, arguably represents the most detailed non‐invasive method to measure ongoing prefrontal activity in human subjects. Baldauf and Desimone (2014) used precisely the combination of these two methodologies (i.e., fMRI and MEG) to uncover the role of the IFJ in object‐based attention. In their paradigm, the participants were instructed to detect the repetition of a target presented among superimposed pictures of faces and houses (Figure 2i). The task was a one‐back repetition, and the participants were cued about which type of stimuli they had to attend (face vs. house) at the beginning of each trial. The stimuli were presented rhythmically at 2 and 1.5 Hz, respectively, by manipulating their phase using a phase‐scrambling technique, thus generating two overlapping streams of frequency‐tagged objects. The authors capitalized on this feature of their experimental design to investigate the coherence between the source and the target nodes of top‐down object‐based attentional modulation. Firstly, they identified the ROIs that were involved in face and house processing (the fusiform face area [FFA] and the parahippocampal place area [PPA], respectively) and spatial, feature‐ and object‐based attention (the FEF and IFJ, respectively) by running three different blocked‐design fMRI localizer tasks. The ROIs obtained by this procedure were then projected on the native cortical surface to analyse MEG signals in source space. Their results showed that, when attending to the preferred stimulus, FFA and PPA increased their coherence with the IFJ in the tagging stimulus frequency and also in the gamma frequency band (60 to 90 Hz), although the specific peak varied considerably between subjects. Furthermore, in nine out of the 12 subjects, the phase lags between the IFJ and FFA and PPA increased linearly depending on the frequencies, suggesting that IFJ cyclic responses were the driver of FFA and PPA responses to the stimuli with an approximate lag of 20 ms (which is presumably due to signal's transmission time). This interpretation is consistent with the communication through coherence framework (Fries, 2005), which postulates that transmitted spikes need to arrive at the right time in order to render the communication between areas effective (within windows of opportunity; that is, periods of maximal depolarization). According to the authors, the IFJ would be indeed best suited to bias neural activity in object identification modules in the temporal lobe by coupling its activity with these sites depending on the task requirements, in a remarkably similar manner to the FEF shifting spatial attention towards different spatial locations.

In the study by Nee and D'Esposito (2016), which innovatively related the IFJ to general principles of functional organization in the PFC (e.g., Koechlin et al., 2003), the authors orthogonally manipulated task demands related to stimulus domain, contextual and temporal control, to engage three main sections of the lPFC (defined rostral, mid and caudal). Their univariate fMRI results revealed that the contextual and stimulus domain manipulation activated the left IFJ but that rostral areas were exclusively employed by the temporal manipulation, reflecting an increased abstraction level in the control of behaviour by these three subregions. Subsequently, they analysed whether, and at which level of the rostro‐caudal axis, stimulus sensitivity emerged. In the caudal PFC, particularly, the dorsal parts (SFS and cMFG) were sensitive to spatial information, whereas the ventral parts were more sensitive to verbal information (IFJ and IFS), but the rostral regions did not show this pattern. Finally, dynamic causal modelling revealed that the mid regions of the IFJ had a stronger efferent connection to both rostral and caudal ones, rather than vice versa, thus positioning them at the apex of the lPFC hierarchy. These results are particularly relevant because they again demonstrate a robust stimulus domain sensitivity in the plPFC and also clarify the directed influence of more rostral sites (i.e., the cMFG and the IFS) to the SFS and IFJ, which may be involved in more integrative cognitive processes, such as spatial‐ and object‐based working memory, and the executive control of action.

We would like to conclude this section by discussing two recent studies on the role of the IFJ in non‐spatial attention. Gong and Liu (2020) investigated whether the IFJ encodes information about the attended feature itself (e.g., which colour is currently attended) using MVPA. In a large sample of 48 subjects, they found that the bilateral IFJ contains a neural representation of the currently attended visual feature. Employing compound stimuli, which consisted of two orthogonal features (i.e., red vs. green colour and clockwise vs. counterclockwise motion), they were able to show that the IFJ had a bias to consistently encode the attended feature better than the unattended. Similarly, Meyyappan et al. (2020) studied the role of the IFJ in feature‐based attention but, most importantly, directly contrasted its involvement in non‐spatial attention to visual features with its involvement in spatial attention by including both forms of top‐down control within a single paradigm. By analysing fMRI activity in individual trials with univariate general linear models and MVPA, they found that the univariate BOLD activity showed no difference in plPFC between feature‐based and spatial‐based attention. However, the MVPA classifier revealed significant decoding accuracy of feature attention in the bilateral IFJ, while the same structure did not encode sufficient information about spatial attention in order to allow above‐chance level classification of the attended location. To explain these results, the authors propose a model in which (the right) IFJ guides feature‐based attention but not (covert) spatial attention during the cue‐target interval.

In summary, although the recent neuroimaging studies that were in part influenced by the hypothesis of spatial versus feature and object selectivity in the plPFC present a more nuanced view of this segregation in the FEF and IFJ, they nevertheless confirm the overarching idea (mainly, thanks to the improvements in data analysis techniques, such as MVPA) that the former is predominantly involved in processing spatial information, whereas the latter is predominantly involved in processing non‐spatial information. Moreover, functional and effective connectivity metrics revealed that the coordination of spatial and non‐spatial selection requires the communication between the FEF and IFJ and posterior areas, in which the FEF assumes the role of a spatial ‘priority map’, whereas the IFJ biases non‐spatial visual processing and acts more as a control structure that allocates attentional resources depending on the task at hand in a flexible way. An interesting question to address seems therefore whether the role of the IFJ in cognition is more multifaceted than the role of the FEF, which could be thought of as more specialized. This would seem reasonable from a comparative perspective because the PFC and the information processing stream in which the IFJ is hypothesized to be embedded (O'Reilly, 2010) is one of the brain districts where humans diverge the most from other primates in terms of its size and relative organization (Donahue et al., 2018; Eichert et al., 2020; Kravitz et al., 2013; Mars, Sotiropoulos, et al., 2018). In the next section, we will attempt to expand on this view by discussing it in the light of the multiple‐demand system hypothesis (Duncan, 2010).

### 3.4 Multiple‐demand system (inclusion in the multiple‐demand system)

In its essence, the multiple‐demand hypothesis is built around the idea that a limited set of brain regions are all involved in many aspects of high‐level, goal‐driven cognitive processes that collectively give rise to intelligent behaviour. The primary sources of empirical support for this hypothesis (together with single‐cell recordings in primates) are fMRI studies that identified overlap in frontal and parietal regions between the activity elicited by a wide range of tasks and resting‐state functional connectivity patterns (Duncan, 2010). Traditionally, the prefrontal areas that are thought to be part of the multiple‐demand system are localized within and posterior to the IFS, thus likely encompassing the IFJ (Duncan, 2010; Fedorenko et al., 2012). Fedorenko et al. (2013) tested the hallmark of the multiple‐demand hypothesis by measuring brain activity using fMRI in several diverse experimental paradigms, which included reading sentences and non‐sentences, performing a memory‐probe task (used as localizer in all the subjects) and optionally performing one or more of the following tasks: a spatial and verbal working memory task, a Stroop task, two versions of the multisource interference task and an arithmetic task. Each task was administered in an easy versus hard version in a blocked experimental design. Crucially, all the analyses were performed in the native subject space to directly probe the degree of overlap in the activations elicited by these paradigms. The results clearly showed that a set of frontal and parietal regions is consistently activated by the global contrast of the respective hard versus easy task versions. Of interest to the scope of the present review, the activations were found all along the premotor regions of the precentral gyrus, in the MFG and in the posterior IFG. It is important to note that although this pattern of activation is consistent with the multiple‐demand system hypothesis, the authors left open the possibility that other dissociations exist among the regions that form part of this system. In support of this possibility, the study by Noyce et al. (2017) attempted to reconcile the multiple‐demand account with the results by Michalka et al. (2015); reviewed in Section 3.1) that reported the existence of sensory selectivity in the lPFC. In their fMRI study, they used two visual and auditory two‐back tasks to probe lPFC responses in a new paradigm modified and adapted from the study by Michalka et al. (2015). With this, they indeed replicated and extended their original finding of four interleaved visual and auditory sensory regions in the lPFC. By leveraging the fact that seven subjects participated in the previous study, they also computed a Dice coefficient on the activations found in the present study, which all had significant spatial overlap (ranging between 0.57 and 0.7). Finally, by averaging visual and auditory activation and contrasting them with the averaged sensorimotor control activation patterns, the authors introduced the concept of a degree of multiple‐demand responsiveness. This degree spanned from areas that did not show any sensory bias, but only working memory activation, in the anterior insula cortex and in the dorsal anterior cingulate cortex/pre‐supplementary motor area (thus labelled ‘pure’ multiple‐demand areas), to areas showing a bias towards visual modality, but also increased activation in the working memory task in their non‐preferred modality (i.e., with auditory stimuli) in the iPCS and the right sPCS (therefore considered ‘partial’ areas). These results are particularly interesting in that they show that the iPCS and sPCS (which are the closest anatomical landmarks to the IFJ and FEF, respectively; see Section 2.2) encode sensory‐specific visual information but that they may as well process general domain signals that globally support attention and working memory functions (Noyce et al., 2017).

In the study by Muhle‐Karbe et al. (2017), the authors investigated the encoding of novel task instructions (i.e., the task set) and its relationship to the multiple‐demand system. In particular, their fMRI study aimed to measure activations during the delay period to disentangle activity related to the preparation of novel stimulus‐response mappings from their simple memorization. The participants were instructed to respond to face and house stimuli by pressing a button with their index or middle finger, or to simply report whether a probe contained matching or mismatching instructions compared with the initial display shown before the delay period. The decoding accuracy of brain activity using MVPA was higher than chance during the delay period in the blocks requiring the implementation of novel stimulus‐response mappings and significantly increased compared with the memorization blocks in the IFJ, the FFA and the PPA. Thus, these results argue in favour of a role of the IFJ in encoding abstract task rules (in this case, stimulus‐response mappings) and in the maintenance of working memory representations, which were less accurately decoded in this study, although they were also likely contributing to the overall activation patterns.

Finally, in the most comprehensive analysis of the multiple‐demand system available to date, Assem et al. (2020) combined the accurate alignment of cortical areas across subjects using improved registration methods (Robinson et al., 2014) with the parcellation of the individual cortex using the MMP1 by Glasser et al. (2016) in a very large sample of subjects (*n* = 449 subjects from the Human Connectome Project; Van Essen et al., 2013) to answer five core questions of the multiple‐demand hypothesis (we will focus here only on the delineation of the multiple‐demand system; the reader is referred to the original publication for further details). By performing a conjunction analysis on three fMRI tasks that should activate the putative multiple‐demand regions (viz., a working memory *n*‐back task, a relational reasoning task and a math/story task), the authors identified areas that formed part of the ‘penumbra’ and the ‘core’ multiple‐demand system by computing the parcels that had stronger mean activations compared with the initial 27 parcels of the ‘extended’ multiple‐demand system. Interestingly, both the area IFJp (activated by the relational reasoning and the math task contrasts) and the area i6‐8 (activated by all three task contrasts and preferentially by the working memory task) that lies immediately anterior to the FEF were included in the core multiple‐demand system. An additional analysis using contrasts with weaker cognitive demands in the working memory and relational reasoning tasks identified additional foci in the FEF and anterior IFJ (IFJa), among others. This suggests that these regions may display partial multiple‐demand characteristics and that this possibility may need further scrutiny. However, as shown by the analysis of resting‐state functional connectivity, the core regions were more strongly interconnected, and all belonged to the frontoparietal network (Ji et al., 2019), confirming the reliability of the multiple‐demand system delineation proposed by the authors.

In conclusion, several papers have now reported the involvement of the IFJ in the multiple‐demand system, thus reinforcing the hypothesis that this area may have a broader role in cognition compared with the FEF. This idea is consistent with the view that the visuospatial attention system is evolutionarily older and its organization is well preserved in primates (Caminiti et al., 2015), whereas the vlPFC, and its projections to the temporo‐occipital cortex, might underlie the emergence of more complex human behaviours (Eichert et al., 2020; Mars, Sotiropoulos, et al., 2018; Neubert et al., 2014).

## 4 CONNECTIVITY FINGERPRINTS

We began the present review by introducing the hypothesis that the function of a brain region is ultimately determined by its intrinsic and extrinsic connectivity fingerprints and its structural properties (Passingham et al., 2002). At a time in which network neuroscience has become so prominent, we would almost take this hypothesis to be self‐evident. However, it wasn't until recently, with the advent of big data approaches (thanks to the availability of extensive neuroimaging public datasets and repositories; e.g., Van Essen et al., 2013), that this hypothesis could be experimentally tested (Mars, Passingham, & Jbabdi, 2018). This section will thus focus, wherever feasible, on such approaches aggregating large amounts of multimodal neuroimaging data to model the structural and functional connectivity of the plPFC. Furthermore, an appropriate emphasis will be put on comparative evidence, particularly in the case of non‐invasive methodologies that allow inferring brain connectivity but have not been thoroughly validated yet (i.e., diffusion magnetic resonance imaging [dMRI]; Donahue et al., 2016; Maier‐Hein et al., 2017; van den Heuvel et al., 2015). In the following sections, we will discuss studies that adopted a seed‐based approach to analyse specific ROIs connectivity to answer well‐defined research questions about the structural and functional connectivity of the plPFC.

### 4.1 Structural connectivity

In contrast to non‐human primates, and in particular, the macaque, in which the tracer studies performed in the last decades have enabled the detailed mapping of the brain's architecture and structural connectivity of this species (Barbas & Pandya, 1989; Felleman & Van Essen, 1991; Kötter, 2004; Markov et al., 2014), in humans, this knowledge is predominantly based on dMRI (Jeurissen et al., 2019). Although this methodology has held the promise of revealing non‐invasively the structural connectivity and white‐matter structures of the human brain at a level of detail comparable with invasive and ex vivo studies, it is still affected by biases, false positives and common methodological misconceptions in the research community itself (Jeurissen et al., 2019; Maier‐Hein et al., 2017). These limitations notwithstanding, it has also allowed researchers to probe the structural connectivity of large populations with a variety of algorithms to investigate a growing set of measures related to major white‐matter bundles, local tissue properties and further down to tissues microstructure. In addition, the combination of dMRI with fMRI allows researchers to investigate the structural connectivity of functionally defined seeds and its relationship to functional connectivity, based on the idea that the underpinning of functional connectivity lies in structural connectivity. Although this relationship is difficult to establish due to inherent methodological limitations and it is certainly still controversial, some studies that combined these methods have shown remarkable results in how structural connectivity can be used to predict task‐fMRI activations in several specialized brain modules (Osher et al., 2016; Saygin et al., 2012).

**FIGURE 3:**
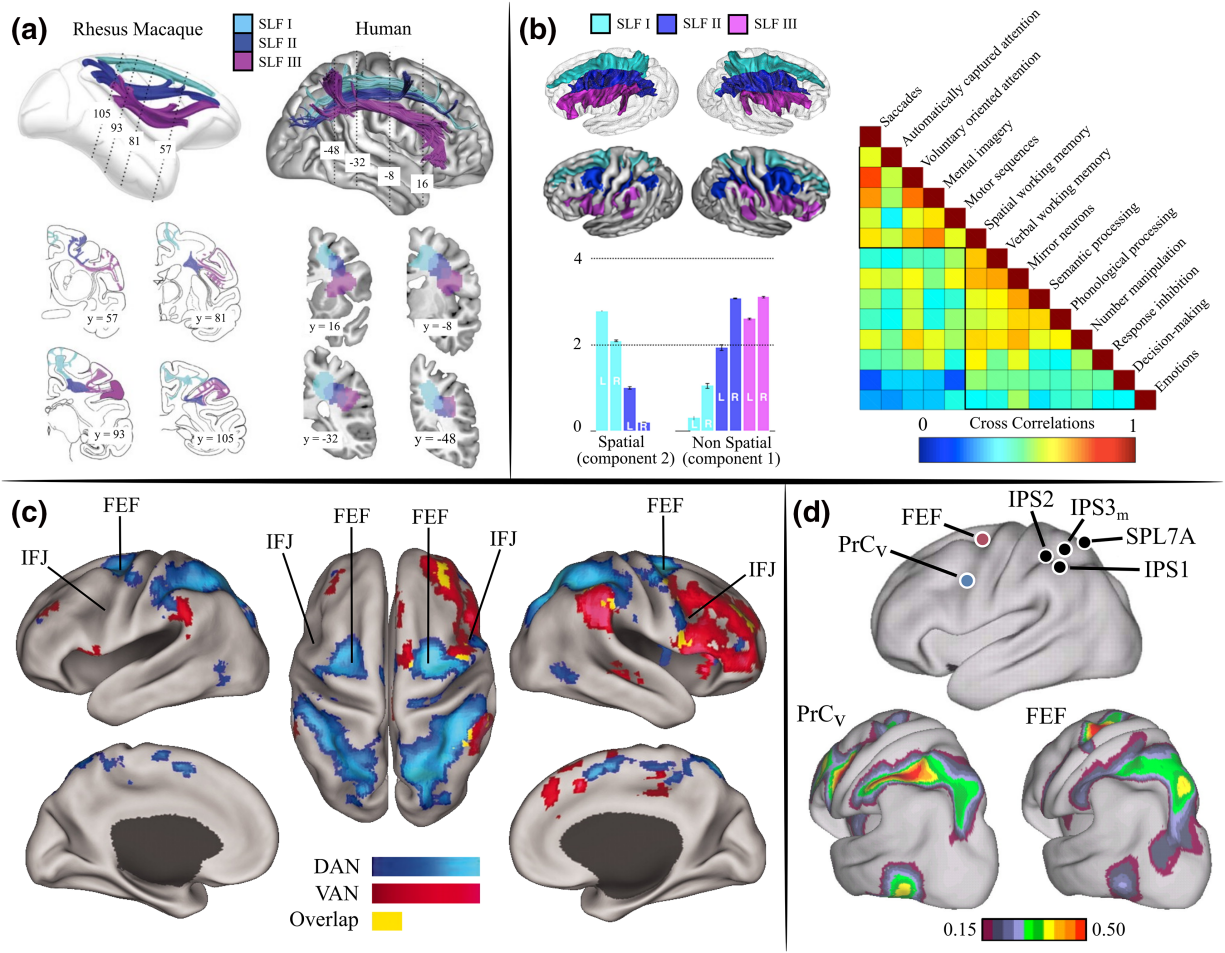
Structural connectivity and resting‐state functional connectivity of the posterior lateral prefrontal cortex. (a) depicts the comparison between the organization of the three branches of the superior longitudinal fasciculus (SLF) in macaques and humans. This frontoparietal bundle was delineated using a virtual dissection protocol in 20 adult participants (adapted from de Schotten et al., 2011). (b) presents a summary of the findings from the study by Parlatini et al. (2017). The activation maps resulting from an fMRI meta‐analysis of 14 distinct cognitive functions were clustered using a principal component analysis in a ‘spatial/motor’ and a ‘non‐spatial/motor’ component (b, right side). These maps' *z* scores were related to the cortical terminations of the three branches of the SLF obtained from a large sample of participants (*n* = 129), suggesting that the SLF1 could preferentially mediate ‘spatial’ processes, whereas the SLF3 could preferentially mediate ‘non‐spatial’ processes. The SLF2 overlapped with both activation components, therefore possibly underlying their interactions (b, left side). (c) The dorsal and the ventral attention networks identified for the first time during resting‐state fMRI in the study by Fox et al. (2006). These spontaneous activity patterns show high resemblance to the two attentional systems proposed by Corbetta and Shulman (2002) and highlight the potential of resting‐state data to reveal stable modes of organization of the attention networks in the absence of task demands (see also De Pasquale et al., 2010, for converging evidence on the organization of the DAN using MEG). (d) Seed‐based analysis of resting‐state fMRI functional connectivity of the FEF and the ventro‐caudal prefrontal region (PrC_v_) from the study by Yeo et al. (2011). The authors defined the former nodes by performing an fMRI meta‐analysis and using a separate discovery sample, respectively, and employed a data‐driven approach to probe their connectivity patterns. In a subsequent stage, they tested them in an independent replication sample in a confirmatory fashion. They found that within the posterior parietal cortex, the medio‐caudal SPL showed increased functional coupling with FEF, whereas the lateral IPS complex showed increased functional coupling with PrC_v_ in their replication sample. These results suggest segregated parallel pathways from the plPFC to the SPL and the IPS complex

In terms of our knowledge of the structural connectivity of the plPFC, as mentioned earlier, comparative evidence provides a solid foundation against which dMRI results in humans can be usefully related (Schall et al., 1995; Stanton et al., 1995; Webster et al., 1994; Yeterian et al., 2012). In one of the earliest comparative dMRI studies, Croxson et al. (2005) described the structural connectivity of the human PFC using probabilistic tractography (Behrens et al., 2003) and compared it with the macaque's, revealing striking similarities in the structural connectivity patterns in the two species. The following studies have primarily focused on establishing a relationship between the PFC association tracts (Croxson et al., 2005) and subregions involved in specific functions, among which visuospatial attention. In an outstanding contribution to the understanding of the structural underpinnings of visuospatial selection, de Schotten et al. (2011) initially compared the organization of the three branches of the superior longitudinal fasciculus (SLF) in the rhesus macaque (atlas‐based) and humans (*n* = 20; for which they had also available ex vivo data from a single subject as ground truth) by performing the virtual dissection of this bundle (Figure 3a). The authors moreover identified the cortical terminations of the SLF and their relationship with the functional activation profiles of the DAN and VAN originally proposed by Corbetta and Shulman (2002). From their study, it emerges that the FEF is reached by the cortical terminations of the SLF1 and SLF2. In contrast, the IFJ is mostly reached by the terminations of the SLF2 and SLF3 (even though it is important to note that in this study these ROIs were only defined at the group level), potentially highlighting a dissociation in the macroscale anatomical connectivity of these two areas. In Umarova et al. (2010), the authors combined fMRI—employed to define the nodes involved in visuospatial processing using an endogenous cueing task—with probabilistic tractography, to analyse their reciprocal structural connectivity in 26 participants. This study showed that, in the right hemisphere, the FEF is wired through the SLF2 to the temporoparietal cortex, revealing a dorsal white‐matter pathway from the FEF passing within the inferior parietal lobule to the IPS, the supramarginal gyrus, the caudal superior temporal gyrus and V5/MT+. The study by Anderson et al. (2012) employed a similar logic to measure the structural connectivity between the nodes involved in oculomotor control, including the FEF, the supplementary eye field and the premotor eye field. These nodes were defined using an fMRI localizer task that consisted of alternating blocks of voluntary horizontal saccades performed in darkness and fixation blocks. The reconstructed streamlines from the FEF showed terminations in the inferior parietal cortex (the anatomically defined IPS1, IPS2 and IPS3), with a higher streamline count in the right hemisphere, potentially suggesting right lateralization of this pathway. Although one limitation of this study is that the tractography model adopted was a deterministic one, these results fit well with the study by Umarova et al. (2010). Moreover, they are consistent with the available evidence in macaques (in particular, of a pathway connecting the FEF with the lateral intraparietal area, the putative homologue of the IPS in humans; Stanton et al., 1995). Finally, Szczepanski et al. (2013) aimed at distinguishing the contribution of DAN nodes to viewer‐ and object‐centred processes (these nodes were defined using a memory‐guided saccade topographic localizer fMRI task). By analysing dMRI data using probabilistic tractography, they showed that the FEF was more likely connected with IPS2 than with SPL1. Vice versa, the supplementary eye field was more likely connected with SPL1 than with IPS2. Their study provides evidence for a model in which FEF‐IPS2 structural and functional connectivity support viewer‐centred processing, whereas the supplementary eye field‐SPL1 structural and functional connectivity support object‐centred processing, therefore effectively dissociating gaze‐ and object‐centred representations. In summary, the reviewed evidence on the FEF suggests that this region exhibits reproducible patterns of structural connectivity with the parietal lobe and particularly with the IPS complex. As we have already reviewed in Sections 3.1 and 3.2, the IPS belongs to the DAN, and it is also topographically organized, while being often proposed as the locus of a priority map of space, similarly to the FEF (Fecteau & Munoz, 2006).

Unfortunately, the seed‐based structural connectivity analysis of the IFJ suffers from a remarkable lack of data. Moreover, to the best of our knowledge, no publication reported evidence on the structural connectivity of the functionally defined IFJ. Baldauf and Desimone (2014) analysed the structural connectivity of the FFA and PPA (localized using fMRI) using probabilistic tractography. They then used the outputs to classify subregions in the PFC based on their probability values. The authors were able to show that the regions that display a higher likelihood of being structurally connected with the FFA are localized in the vicinity of the IFJ, in the *pars triangularis*, the *pars opercularis* and the cMFG. However, the extrapolation from these results of structural connectivity patterns in the opposite direction (viz., from the IFJ to FFA and PPA) is prevented by the fact that probabilistic tractography results are strongly affected by the location where the streamlines are initialized to propagate. Thus, evidence from the seed‐based analysis of the structural connectivity of the IFJ is needed to complement these findings. Finally, the authors also used a deterministic tractography algorithm to perform a virtual dissection of the white‐matter bundles that connect the PPA and the FFA to the *pars opercularis* (which includes part of the IFJ) and suggested that the uncinate fasciculus underlays these structural connectivity pathways.

Finally, one of the most significant contributions to the characterization of the relationship between the structural connectivity and the function of the plPFC can be found in the study by Parlatini et al. (2017). The authors combined the fMRI meta‐analysis of 14 different cognitive domains with the virtual dissection of the SLF in a large sample of participants (*n* = 129; Figure 3b). The study aimed to reveal how the three branches of the SLF relate to the functional specializations of the areas that belong to the frontoparietal network. Importantly, the virtual dissection of the SLF was performed using a modified version of spherical deconvolution (Dell'Acqua et al., 2010), which allowed it to preserve its organization in three distinct branches, despite the presence of crossing fibres near the corona radiata. In their meta‐analysis, the authors clustered frontoparietal regions' co‐activations into two components using a principal component analysis. This analysis revealed that two components, referred to as a ‘spatial/motor’ component and a ‘non‐spatial/motor’ component, were together able to account for 70% of the co‐activation data variance. Crucially, the average *z* scores (thresholded at 50%) of these components differentially loaded on the SLF1 and SLF3 cortical terminations, respectively. The SLF2 terminations instead overlapped with both components (for which the shared activations were mainly localized within BA6 in the PFC), and this bundle was therefore suggested to underlie the interactions between these two functional components. Based on the figures from this study (in particular, fig. 6; shown in the left middle row of Figure 3b), we speculate that the IFJ may be localized near SLF2 and SLF3 terminations, thus suggesting anatomical pathways through which the IFJ could mediate ‘non‐spatial/motor’ functions. We therefore propose that future studies should specifically address the question of how the cortical terminations of the three branches of the SLF relate to functional subdivisions of the PFC.

### 4.2 Resting‐state functional connectivity

One of the major breakthroughs in the study of the brain networks involved in visual attention has been the observation that the coupling between BOLD activity in different brain regions measured during the performance of attentional tasks is preserved in resting‐state conditions (Fox et al., 2006; Figure 3c). These spontaneous activity patterns again confirmed the existence of a bilateral DAN (with the FEF and IPS as main nodes) and a right‐lateralized VAN (with the ventral frontal cortex and TPJ as main nodes). Due to the overlap of activations in the right MFG and IFG, Fox et al. (2006) suggested that these nodes could mediate the interaction between the DAN and VAN. Importantly, in a subsequent study by De Pasquale et al. (2010), the authors used fMRI coordinates to define the DAN nodes in MEG source space and validated the segregation of this network and its correspondence with the network isolated by resting‐state fMRI. Such multimodal combinations highlight the potentiality of MEG to characterize the spectral characteristics of resting‐state networks and their temporal dynamics (De Pasquale et al., 2010). Therefore, one of the most intriguing questions is how spontaneous neural activity (as measured with fMRI or MEG) could provide a window into the reciprocal interactions and the functional differentiation of the FEF and IFJ. Furthermore, it would be fascinating if such resting‐state activations can be used to subdivide the attention networks at an even finer grain, for instance, by stimulus domain (i.e., spatial vs. non‐spatial), rather than the more prevalent top‐down versus bottom‐up functional dichotomy (Corbetta & Shulman, 2002).

An alternative classification of the resting‐state network organization of the plPFC was presented in the fMRI study by Cole and Schneider (2007). To define their ROIs, the authors used a modified search task that entailed working memory encoding and maintenance while allowing the separation of these processes from stimulus‐response mapping. They subsequently analysed the functional connectivity between these previously defined six bilateral ROIs, including the IFJ and dorsal premotor cortex, in resting‐state conditions. They showed that these nodes had higher functional connectivity than six different nodes engaged in domain‐specific processes, highlighting a cognitive control network (Cole & Schneider, 2007). In a similar vein, Vincent et al. (2008) analysed the seed‐based functional connectivity of the anterior PFC to probe whether this region belongs to an anatomically segregated control network that could mediate the communication between the anti‐correlated DAN and the hippocampal‐cortical memory system (a component of the default mode network). A remarkable methodological strength of this study is that the bilateral aPFC network was delineated in a first dataset (*n* = 10), but the remaining seeds (MT+ and the hippocampal formation) were defined through a correlation analysis in a second dataset, and finally that the segregation of the networks was tested in a yet third, independent dataset (*n* = 50 and *n* = 45). The authors were able to show that a frontoparietal network is interposed between the DAN and the hippocampal‐cortical memory system with minimal overlap. An important refinement of the proposed role of the frontoparietal network was formalized by Cole et al. (2013). The authors of this study tested participants in a variety of fMRI tasks involving the implementation of novel instructions and showed that the frontoparietal network exhibited two general properties that are consistent with its role as a flexible hub network: (1) greater global variable connectivity compared with the other resting‐state networks and (2) compositional coding of information as reflected in its activity patterns during task performance. Following this evidence, and the studies reviewed in Section 3.4, it would seem reasonable to implicate the IFJ in the frontoparietal network, in accordance with its role as an executive control structure that belongs to the multiple‐demand system (Assem et al., 2020).

In one of the most influential resting‐state fMRI network parcellation (Yeo et al., 2011; *n* = 1000), the FEF (as defined through an fMRI meta‐analysis) was reported to be part of the DAN and showed increased functional connectivity with the caudal IPS and SPL, whereas the ventro‐caudal frontal region (termed the PrC_v_, and defined in an independent fMRI ‘discovery sample’; Yeo et al., 2011; likely encompassing the IFJ), which was also part of the DAN, showed increased functional connectivity with ventral parts of the rostral IPS and SPL (Figure 3d). In their replication sample, the authors again found evidence of increased functional coupling between FEF and the medio‐caudal SPL and between PrC_v_ and the rostro‐lateral IPS complex. This combination of a data‐driven and a confirmatory approach represents a powerful methodology to uncover novel organizational principles of functional interactions between the PFC and posterior areas. Finally, according to the recent resting‐state fMRI network partition by Ji et al. (2019; whose nodes are in turn based on the MMP1 parcellation), the FEF is part of the cingulo‐opercular network, and the IFJp is part of the frontoparietal network, whereas the IFJa is part of a newly defined language network.

### 4.3 Meta‐analytic connectivity modelling

The plPFC engages with multiple regions dispersed all over the brain, making the process of reporting its co‐activation patterns in all possible experimental scenarios a daunting task. However, thanks to the technique known as meta‐analytic connectivity modelling (MACM; Robinson et al., 2010), the results from multiple fMRI experiments involving a common seed region can be efficiently summarized, used to probe specific hypotheses about regional segregation and made accessible to the neuroscientific community. In the last decade, several studies have used this meta‐analytic technique to model plPFC co‐activations, including the FEF and IFJ. Sundermann and Pfleiderer (2012) published one of the most detailed analyses of the functional connectivity of the IFJ, combining an ALE, MACM and an independent resting‐state fMRI analysis. The authors used the coordinates provided in Derrfuss et al. (2005) to retrieve from the BrainMap database (http://brainmap.org/) all the foci that were co‐activated with the putative IFJ seed, in a way that effectively makes their ROI definition agnostic for anatomical descriptors and functional specialization of this region. Their MACM results (which were drawn from 180 experiments for the left IFJ and 131 for the right IFJ) reveal that the IFJ is robustly co‐activated with the dlPFC and the vlPFC, the MFG/pre‐supplementary motor area, the anterior insula, the posterior parietal cortex and the occipitotemporal junction, among others. The authors interpret these findings as largely consistent with the proposal advanced by Cole and Schneider (2007) that identifies the IFJ as one of the crucial nodes of the cognitive control network, an interpretation that was further supported by the resting‐state fMRI analysis (Sundermann & Pfleiderer, 2012). In Muhle‐Karbe et al. (2016), a data‐driven seed‐based parcellation was performed on the lPFC using k‐means clustering. The activation patterns of the six ensuing clusters were characterized using MACM. Cluster 1, localized at the intersection of the iPCS and the IFS, matched the IFJ coordinates reported in previous studies (Derrfuss et al., 2004, 2009) and co‐activated with the rIFJ, lIPS, right anterior insula and bilateral pre‐supplementary motor area. In a follow‐up study by Ngo et al. (2019), the authors applied the author‐topic model (https://github.com/ThomasYeoLab/Ngo2019_AuthorTopic) to find subcomponents in the co‐activation patterns of the left IFJ cluster reported in the previous study by Muhle‐Karbe et al. (2016). They showed three main components that could describe these patterns: a first component related to language processing, a second component involved in attentional control and a third one, which they speculated may reflect inhibition and response conflict resolution. Of interest to our present purpose, the second component engaged the bilateral IPS and SPL, which seems to fit the functional role attributed to this co‐activation pattern. Thus, in addition to the analytic solution afforded by MACM, the author‐topic models represent a viable and promising approach to fractionate the functional domains in which the IFJ is involved and could, for example, be used to contrast the function of the IFJ in the two hemispheres.

Perhaps because of its more consensually established functional role, the only study that we are aware of providing evidence on the FEF co‐activation patterns is the one by Cieslik et al. (2016). The authors combined a coordinate‐based meta‐analysis and MACM to dissociate the oculomotor regions involved in anti‐saccade and pro‐saccade task performance (Cieslik et al., 2016). The lateral FEF was identified through an ALE meta‐analysis focusing on pro‐saccade versus baseline/fixation fMRI contrasts, whereas the medial FEF was identified by anti‐saccade versus pro‐saccade contrasts. Interestingly, these contrasts identified some regions of overlap but also showed that these two foci of activation were partially dissociable. In turn, MACM revealed that the lateral FEF showed increased functional connectivity with motor regions, whereas the medial FEF showed distinct functional connectivity patterns that were related by the authors to the multiple‐demand system (Fedorenko et al., 2013). While these results are certainly intriguing, one limitation of the study is that the coordinate‐based meta‐analysis was underpowered; hence, the hypothesis of segregation of the FEF based on the involvement in anti‐saccade and pro‐saccade performance needs to be further explored in future studies.

To conclude, in this section, we reviewed several metrics of brain connectivity, including structural connectivity as inferred from dMRI, functional connectivity during resting state measured using fMRI and MEG, and MACM. These metrics suggest that the FEF displays robust structural connectivity patterns with the IPS complex, consistent with comparative evidence, whereas the structural connectivity of the IFJ remains largely unexplored. During resting state, FEF activity forms the backbone of the DAN (along with IPS and MT+) in both fMRI (Fox et al., 2006; Yeo et al., 2011) and MEG (De Pasquale et al., 2010), whereas IFJ activity patterns seem to be better characterized as belonging to a frontoparietal (alternatively, a cognitive control) network (Cole & Schneider, 2007; Ji et al., 2019). Resting‐state MEG could be therefore used to measure IFJ dynamic functional connectivity to assess the robustness of its assignment to this network over time. The IFJ similarly co‐activates with overlapping regions of the frontoparietal network in a variety of tasks as revealed by MACM, but the author‐topic model (Ngo et al., 2019) suggests that these co‐activation patterns underlie at least three different cognitive subcomponents. We propose that the convergent application of these techniques to the study of the FEF could be used to differentiate its cognitive subcomponents and to potentially compare the topography of its co‐activation patterns in attentional control tasks with the ones previously reported in the IFJ (Ngo et al., 2019).

## 5 GENERAL DISCUSSION AND FUTURE DIRECTIONS

In the present review, we discussed recent neuroimaging evidence suggesting that although the FEF and IFJ are implicated in similar and overlapping cognitive functions, they have remarkably distinct roles in shaping goal‐driven behaviour (Baldauf & Desimone, 2014; Tamber‐Rosenau et al., 2018; Zanto et al., 2011; Zhang et al., 2018). These differences are not apparent when considering their structure, localization, selectivity to sensory modalities or their functional specialization alone, but only when the broader spectrum of their activation in response to attentional, working memory and executive tasks, and crucially, their connectivity fingerprints (Mars, Passingham, & Jbabdi, 2018; Passingham et al., 2002) are considered. This likely reflects the intrinsic difficulty of identifying functional specializations in regions that in primates are by nature flexibly engaged in a variety of high‐level cognitive mechanisms (Duncan, 2010; Fuster, 2001; Goldman‐Rakic, 1996; Miller & Cohen, 2001). Taken together, the studies reviewed indicate that when we compare the FEF and IFJ on these broader aspects, these regions' functional roles and connectivity differ considerably. Our main results are summarized in Table 2 for each of the aspects that were contrasted in this review, along with the main references consulted.

**TABLE 2:** Summary of the main findings reviewed

Domain	Section title	Summary of the findings	Main references
Structure	Cytoarchitecture, chemoarchitecture and receptorarchitecture	The cytoarchitecture of the FEF is dysgranular. This region is part of BA6 and, to a lesser extent, of BA8. Its chemoarchitecture segregates it from the superior and the middle frontal gyrus. The cytoarchitecture of the IFJ is also dysgranular. This region lies in BA6, BA8 and BA44. Its receptor fingerprint segregates it from the ventral 44d. Receptorarchitecture also allows to segregate the IFJ in two distinct subregions.	Amunts et al. (2010); Amunts and von Cramon (2006); Petrides and Pandya (1999, 2002); Rosano et al. (2003); Zilles and Amunts (2018).
Structure	Localization and relation to sulcal morphology	The FEF is localized ventral to the junction of the sPCS and the SFS. The IFJ is localized dorsal to the junction of the iPCS with the IFS.	Amiez et al. (2006); Derrfuss et al. (2009, 2012); Koyama et al. (2004).
Function	Sensory domains (sensory vs. supra‐modal coding) and topographic organization	The FEF and IFJ are both primarily selective for visual information, but only the FEF contains a full topographic map of contralateral space.	Hagler and Sereno (2006); Kastner et al. (2007); Mackey et al. (2017); Michalka et al. (2015); Wang et al. (2015).
Function	Top‐down versus bottom‐up spatial attention	The FEF and IFJ are both involved in top‐down attention and show evidence of sustained activity in response to a cue. However, IFJ activity profile is more context dependent and influenced by bottom‐up factors as well. The role of the IFJ could be of modulating DAN activity according to the current task demands and of toggling between DAN and VAN activities.	Asplund et al. (2010); Corbetta and Shulman (2002); Marshall et al. (2015); Ruff et al. (2006); Tamber‐Rosenau et al. (2018); Vossel et al. (2012); Wen et al. (2012); Zanto et al. (2011).
Function	Spatial versus non‐spatial attention and spatial versus object working memory	The FEF is predominantly involved in processing spatial information, mediating the set of overlapping functions of covert spatial attention, oculomotor control and spatial working memory. In contrast, recent studies using MVPA revealed that the IFJ is involved in processing non‐spatial information (i.e., in feature‐ and object‐based attention and working memory tasks). The analysis of effective connectivity has also allowed to identify the IFJ as the source of modulation of feature‐based attention and working memory encoding signals.	Baldauf and Desimone (2014); Gong and Liu (2020); Jerde et al. (2012); Liu (2016); Liu et al. (2011); Nee and D'Esposito (2016); Sneve et al. (2013); Zanto et al. (2010); Zhang et al. (2018).
Function	Multiple‐demand system (inclusion in the multiple‐demand system)	Several studies have now reported that the IFJ (and in particular, the IFJp) belongs to the core multiple‐demand system. There are also indications that the IFJa and FEF may display partial multiple‐demand characteristics, although this possibility needs to be further researched.	Assem et al. (2020); Duncan (2010); Fedorenko et al. (2013); Muhle‐Karbe et al. (2017); Noyce et al. (2017).
Connectivity	Connectivity fingerprints (structural connectivity, resting‐state functional connectivity, meta‐analytic connectivity modelling)	The FEF is reached by the terminations of the SLF1 and SLF2. In contrast, we speculate that the IFJ may be reached by the terminations of the SLF2 and SLF3, thus suggesting partially segregated anatomical pathways from the plPFC to posterior parietal and temporoparietal cortices. The FEF is one of the core regions of the DAN, whereas the IFJ is part of the frontoparietal network. Meta‐analytic connectivity modelling reveals three main co‐activation patterns in the left IFJ, including a pattern related to attentional control.	Cieslik et al. (2016); Cole and Schneider (2007); De Pasquale et al. (2010); de Schotten et al. (2011); Fox et al. (2006); Ji et al. (2019); Ngo et al. (2019); Parlatini et al. (2017); Sundermann and Pfleiderer (2012); Vincent et al. (2008); Yeo et al. (2011).

Abbreviations: BA, Brodmann area; DAN, dorsal attention network; FEF, frontal eye field; IFJ, inferior frontal junction; IFJa, anterior inferior frontal junction; IFJp, posterior inferior frontal junction; iPCS, inferior precentral sulcus; MVPA, multivariate pattern analysis; SFS, superior frontal sulcus; SLF, superior longitudinal fasciculus; sPCS, superior precentral sulcus; VAN, ventral attention network.

In this work, we have purposefully emphasized a perspective on functional specialization in the PFC that focuses on the representational format (spatial vs. non‐spatial) and that was pioneered thanks to the comparative studies by Goldman‐Rakic and colleagues (Goldman‐Rakic, 1996; Romanski, 2004; Scalaidhe et al., 1997, 1999; Wilson et al., 1993). Although we think that this is one of the most valuable frameworks to understand the organization of the plPFC, there are several other principles of organization of the PFC that are worth taking into account. Among these, the level of abstraction and the difficulty level of the task performed are the two most important factors that are usually invoked to explain gradients in its organization, typically along the rostro‐caudal axis (Koechlin et al., 2003; Nee & D'Esposito, 2016; O'Reilly, 2010). We would like therefore to conclude this review by presenting several open questions that are in our opinion the most urgent and intriguing to further disentangle which are the peculiar contributions of the FEF and IFJ to goal‐driven behaviour and particularly to top‐down attention and working memory (Table 3).

**TABLE 3:** Open questions and future directions

1	Based on connectivity fingerprint matching, which region is more likely to be the homologue of the human IFJ in the macaque?
2	Are the FEF and IFJ reliably tied to predefined sulcal landmarks at the individual subject level? What is the role of inter‐individual differences in sulcal organization in localizing these regions?
3	Does the IFJ encode information at a more abstract level compared with the FEF? Within the IFJ, are there different levels of abstraction encoded in distinct neural populations, or is there a gradient of increasing abstraction from the posterior to the anterior IFJ?
4	Are the structural connectivity fingerprints of the FEF and IFJ indicative of a segregation in their afferent and efferent connections with the dorsal and the ventral visual streams?
5	Similarly, are these connectivity fingerprints recapitulated in resting‐state fMRI and MEG activity? Can these be used to fractionate the attention (i.e., the DAN and VAN) and frontoparietal networks according to the representational content encoded (spatial vs. non‐spatial)?
6	Can we causally demonstrate a dissociation between the role of the FEF and IFJ in the control of spatial versus non‐spatial attention using TMS?

Abbreviations: DAN, dorsal attention network; FEF, frontal eye field; fMRI, functional magnetic resonance imaging; IFJ, inferior frontal junction; MEG, magnetoencephalography; TMS, transcranial magnetic stimulation; VAN, ventral attention network.

Given that many sources of evidence of a dissociation between the selectivity to spatial, as opposed to feature and object information, in the plPFC are comparative (Bichot et al., 2015, 2019; Constantinidis & Qi, 2018; Goldman‐Rakic, 1996; Riley et al., 2017; Wilson et al., 1993), we would like to discuss some of the most noteworthy proposals of quantitative approaches establishing homologies in the plPFC in humans and macaques. In this regard, several challenges ought to be addressed. First, the lack of similar sulcal organization in macaques and the relative expansion in the size of the human PFC (Donahue et al., 2018) complicate any solution that relies on the purely geometrical inter‐species registration of these areas. Second, even though cytoarchitecture may serve as a guiding principle in identifying homologies, in the PFC, it is usually at best a coarse approximation, for example, as illustrated by the fact that FEF lies in different BAs in the macaque and the human (viz., BA8 and BA6, and BA6, respectively; Paus, 1996; Petit & Pouget, 2019; Tehovnik et al., 2000). This implies that the cytoarchitecture of the PFC in the macaque isn't necessarily a good predictor of the location where fMRI activity is usually measured using conventional FEF localizer tasks in humans (for instance, see fig. 2C from Amiez et al., 2006). The third challenge is methodological: in humans, the cortical organization is usually inferred non‐invasively with MRI, whereas invasive methods are still the most widely adopted to study non‐human primates. As a particularly glaring example, consider the definition of structural connectivity in macaques and humans: the first inferred from tracing studies on small populations of animals, but with a negligible number of false positives, whereas the latter is inferred non‐invasively from large populations, but where current tractography algorithms are only partially able to reproduce the former results (Donahue et al., 2016; van den Heuvel et al., 2015). Also, the latter do not yet offer a convenient solution to the filtering of large amounts of false positives (Maier‐Hein et al., 2017).

We review below some recent proposals that may allow overcoming these limitations. The problem of registering cortical maps between species (i.e., from the macaque to the human, and vice versa) has been recently addressed by employing advanced registration algorithms (i.e., multimodal surface matching; Robinson et al., 2014) that can simultaneously rely on multiple cortical features. Although the specific approach adopted in comparative studies may differ in relation to the research question addressed, and in particular in the way structural (e.g., myelin content; Eichert et al., 2020) as opposed to functional information (e.g., gradients; Xu et al., 2020) is exploited to drive the registration, this framework represents a promising solution to cross‐species mapping. In parallel to these advancements, the field of primate neuroimaging is witnessing a rapid acceleration in the development of MRI data acquisition protocols, analysis pipelines and data sharing (Autio et al., 2020; Hayashi et al., 2020; Milham et al., 2018). These developments will likely lead to the creation of a macaque multimodal parcellation from a large cohort of animals, comparable with last decade's research trends in humans (e.g., Glasser et al., 2016; Yeo et al., 2011), derived from the combination of invasive and non‐invasive methods. This will allow researchers to propose an updated cortical taxonomy in this species, and this may in turn allow the precise mapping of homotopic brain regions between macaques and humans (Van Essen et al., 2019).

In two highly influential implementations of comparative neuroimaging, Sallet et al. (2013) and Neubert et al. (2014) used dMRI data to perform a k‐means clustering based on structural connectivity (as inferred from probabilistic tractography) to parcel the dorsal and the ventral human PFC, respectively. They then computed the functional connectivity from resting‐state fMRI data in humans (from the previous parcellation) and macaques (based on an existing cytoarchitectonic atlas) to compare the connectivity fingerprints of each area between the species. They showed that the similarity of these connectivity fingerprints, as measured by the functional coupling scores, allowed them to quantitatively identify homologies in the PFC across these species. In their studies, the human ‘FEF‐like’ cluster corresponded to area 8A in the macaque (denoted cluster 8/8A; Sallet et al., 2013), and the IFJ to area 44 in the macaque (Neubert et al., 2014). In an additional analysis using an ROI that merged the 8A cluster from the study by Sallet et al. (2013) with the IFJ, Neubert et al. (2014) performed an additional connectivity‐based parcellation and confirmed that their IFJ parcel was distinct from the former in all the subjects analysed (*n* = 25). However, a limitation of these studies is that they used already established homologies between the macaque and the human brain to identify the target regions and compute the connectivity fingerprints of their seeds in PFC. Furthermore, in the macaque, all these regions (both seeds and targets) were based on cytoarchitectonic definitions (Neubert et al., 2014; Sallet et al., 2013). It is unclear at present how well these criteria represent functionally meaningful subdivisions of the PFC and if these are adequate to establish such homologies.

An extremely interesting development of this connectionist framework posits that the most appropriate method for establishing homologies should be grounded on the cross‐species alignment of white‐matter bundles (Mars, Sotiropoulos, et al., 2018). If we indeed assume that certain white‐matter bundles are preserved across species, the connectivity blueprints of the vertices localized near their terminations (which essentially are matrices that describe the connectivity of these vertices on the grey‐matter surface with each bundle) can allow us to disentangle between the alternative interpretations of areal expansion in size (if they relate to these bundles in a similar fashion), their relocation across the cortical sheet or their connectional reorganization relative to the last common ancestor (if they are highly dissimilar). In addition to its intuitive appeal, because most of these bundles have been identified (Mars, Sotiropoulos, et al., 2018, refer to 39 bundles as homologues), and their segmentation using dMRI has been already validated for most of them using ex vivo data in both species, this proposal is undoubtedly one of the most promising avenues for macro‐anatomical alignment of functional areas. Using these connectivity blueprints, Mars, Sotiropoulos, et al. (2018) were able to predict the localization of MT+ in the macaque from human hMT+ and, conversely, to predict the localization of the pre‐supplementary motor area in humans from macaque F6, using dMRI data acquired in both species. Concerning the specific problem of identifying FEF and IFJ homologies across primates, it would be therefore interesting to address the following questions (see the first question in Table 3): Do the human connectivity fingerprints and connectivity blueprints of these areas match well with those found in the macaque PFC? If so, which region is the homologue of the human IFJ in the macaque (see Bichot et al., 2015, 2019, and Figure 1 for potential correspondences)? Are any differences explained by the connectivity reorganization in the plPFC, the relocation of its areas, or are they consistent with their expansion in size? Finally, is there any evidence that the SLF, one of the major frontoparietal bundles, has similar cortical terminations in the human and other primates (de Schotten et al., 2011; Hecht et al., 2015)? Answering these questions will enable us to better position the divergence of FEF and IFJ functions along primates' evolutionary path, as well as to perform inferences motivated by comparative evidence on humans in a more reliable way.

A question that is strongly intertwined with the former is whether at present our knowledge of the localization of the FEF and IFJ in standard space (i.e., Talairach and MNI152 space) can be considered reasonably accurate and robust in terms of inter‐individual differences in the sulcal organization (see question two in Table 3). As we have argued in Section 2.2, the FEF and IFJ seem to be tied in a very reliable way to specific sulcal landmarks of the plPFC, although evidence from fMRI analyses performed at the single‐subject level is to date quite limited (Amiez et al., 2006; Derrfuss et al., 2009, 2012; Kastner et al., 2007). In Glasser et al. (2016; see figs 7 and 8 in the ‘Supplementary Results and Discussion’), the authors reported that in 24 subjects out of 210, area 55b (ventrally adjacent to the FEF) had an atypical organization. In nine of these subjects, its localization was shifted dorsally, and in 12 other subjects, this area was split so that the FEF joined its border with the ventral premotor eye field, hence also possibly affecting the average localization of these neighbouring regions. A worrying consequence of this potential spatial uncertainty is that it would severely limit the conclusions reached by any study of brain connectivity (whether based on structural, functional or both information) that did not identify the FEF and IFJ using separate fMRI localizers. In Tables 4 and 5, we have listed the standard coordinates of the FEF and IFJ from the studies discussed in this review. As shown in these tables, the variability in the average localization of the FEF and IFJ seems quite remarkable, particularly along the sagittal plane in the case of the FEF (see Table 4). This may not only be due to genuine inter‐individual variability in the sulcal organization (in addition to the variability in the acquisition sequence, MRI scanner and the often‐large smoothing factor adopted in most fMRI analysis pipelines) but also to the idiosyncratic experimental tasks, contrasts and analytic procedures that were adopted in these fMRI studies to localize the FEF and IFJ. An intermediate solution, in the cases in which running an fMRI localizer is not a viable option, would be to rely on the results of meta‐analytic methods, such as the ALE technique (Eickhoff et al., 2012). Even though this technique should be able to approximate well the typical localization of the FEF and IFJ, we suggest that to infer the most accurate localization of the functionally defined FEF and IFJ in standard space, one would need to additionally define a gold standard for FEF and IFJ fMRI localizer tasks to better refine the study inclusion criteria. In the case of the FEF, we would imagine that reaching a consensus would not be too difficult, as the involvement of this area in oculomotor control has been studied extensively (Paus, 1996; Petit & Pouget, 2019; Vernet et al., 2014). In contrast, due to the involvement of the IFJ in several and potentially non‐overlapping cognitive functions (Ngo et al., 2019; Sundermann & Pfleiderer, 2012), an effort of specifying a set of core processes to consistently localize this region in both hemispheres would be needed, which at present seems challenging (but see Derrfuss et al., 2005, for a relevant suggestion).

**TABLE 4:** List of the group‐level coordinates of the frontal eye field (FEF) in standard space from the studies discussed in this review

Study	*N*	Age	Method/paradigm	Studies/contrast	Space	Hemisphere	Coordinates		
							*x*	*y*	*z*
Amiez et al. (2006)*	8	28; 22–42	Functional localizer	Prosaccade > Fixation	MNI	L	−38	−9.8	54.4
						R	34.5	−10.8	52.5
Anderson et al. (2012)*	10	29 ± 3	Functional localizer	Voluntary saccades in darkness > Fixation	MNI (SPM2)	L	−60	4	38
						R	52	−6	46
Cieslik et al. (2016)	12 experiments	Healthy adults	ALE fMRI meta‐analysis	Prosaccade > Rest and Prosaccade > Fixation (conjunction analysis)	MNI	L	−40	−2	50
						R	44	4	50
De Pasquale et al. (2010)	10	29 ± 6	Literature review	Sources: He et al. (2007); Shulman et al. (1997)	MNI	L	−26.3	−11.8	52.7
						R	30.3	−12.8	52.6
Kastner et al. (2007)*	4	20–36	Functional localizer	Prosaccade > Fixation	Talairach	L	−35	−12	49
						R	36	−9	50
Koyama et al. (2004)*	20	N.A.	Functional localizer	Prosaccade > Fixation	MNI (SPM99)	L	−26	−12	54
						R	18	−6	56
Meyyappan et al. (2020)	20	N.A.	Endogenous cueing paradigm	Spatial and feature‐based task cue‐evoked activity	MNI	L	−27	−1	52
						R	36	2	49
Michalka et al. (2015)	10	27.1; 22–31	Cued RSVP	Attend visual > Attend auditory	MNI (Freesurfer)	L	−33	−7	47
						R	34	−6	48
Nee and D'Esposito (2016)	23	19.9; 18–28	Executive control paradigm	Main effects of temporal and contextual control and their interaction with feature control	MNI	L	−24	4	54
Paus (1996)	60	N.A.	PET meta‐analysis	Oculomotor tasks	Talairach	L	−32	−2	46
						R	31	−2	47
Ruff et al. (2006)*	4	29–35	Functional localizer	Voluntary saccades in darkness > Rest	MNI (SPM2)	R	33	1	62
Tamber‐Rosenau et al. (2018)*	10 (8 L, 10 R)	28.5 ± 3.3	Functional localizer	Prosaccade > Fixation	Talairach	L	−23.5	−9.88	45.75
						R	25.1	−9.8	45.2
Umarova et al. (2010)*	26	30 ± 7.3	Functional localizer	Attend blocks > Fixation	MNI (SPM5)	R	51	3	48
Vossel et al. (2012)*	24	26.83; 20–37	Functional localizer	Valid trials > Baseline	MNI single subject (SPM8)	L	−28.7	−6.3	50.8
						R	34.6	−1.9	50.8
Wen et al. (2012)*	12	20–28	Functional localizer	Attend > Passive view	MNI (SPM2)	L	−30	−3	54
						R	30	0	57
Yeo et al. (2011)	7 studies	N.A.	Meta‐analysis	Saccadic eye movements	MNI	L	−26	−6	48
Zhang et al. (2018)	19	19–26	Feature‐based attention paradigm	All voxels activated by the stimulus block	Talairach	L	−42	−5	35
						R	40	−5	39
Average coordinates*	7 studies (*n* = 86)		All functional localizers	Covert attention/Prosaccades > Fixation	MNI	L	−34.6 ± 12.31	−5.42 ± 6.28	50.24 ± 6.99
	9 studies (*n* = 118)					R	35.71 ± 10.77	−2.96 ± 4.85	53.19 ± 5.55

*Note*: We computed the average between all the coordinates reported from conventional FEF localizers. The coordinates that were originally reported in Talairach space were converted to MNI space using the GingerALE utility convert foci (Eickhoff et al., 2012; Lancaster et al., 2007). As shown, the variability in the average localization of the FEF is quite remarkable, particularly along the sagittal plane (standard deviation > 1 cm).

Abbreviations: ALE, activation likelihood estimation; fMRI, functional magnetic resonance imaging; N.A., not available; PET, positron emission tomography.

**TABLE 5:** List of the group‐level coordinates of the inferior frontal junction (IFJ) in standard space from the studies discussed in this review

Study	*N*	Age	Method/paradigm	Studies/contrast	Space	Hemisphere	Coordinates		
							*x*	*y*	*z*
Asplund et al. (2010)*	30	N.A.	RSVP paradigm	Surprise trials > Search trials	Talairach	L	−40	8	25
						R	37	5	29
Baldauf and Desimone (2014)*	12	23–37	Object‐based attention paradigm	Attend face and attend house blocks > Passive view	MNI (SPM8)	L	−38	11	30
						R	47	9	31
Cole and Schneider (2007)*	9	19–42	Working memory paradigm	Target switching trials target non‐occluded > Non‐switching trials target non‐occluded	Talairach	L	−46	2	36
						R	42	8	31
Derrfuss et al. (2005)	14 Switching and 11 Stroop studies	N.A.	ALE fMRI meta‐analysis	Task‐switching, set‐shifting and S–R reversal studies Colour–word Stroop studies	Talairach	L	−40	4	30
						L	−40	4	32
Derrfuss et al. (2009)*	14 (13 R)	24 ± 1.9	Task‐switching paradigm	Meaning‐switch trials > Cue‐switch trials	Talairach	L	−39	2	32
						R	42	2	31
Derrfuss et al. (2012)*	12	25.3 ± 2.4; 22–31	Stroop paradigm	Incongruent trials > Congruent trials	MNI	L	−42	6	33
Han and Marois (2014)*	14	20–32	RSVP/oddball paradigm	Target trials > Distractor trials	Talairach	L	−45	3	22
						R	44	5	26
Meyyappan et al. (2020)*	20	Undergraduates	Endogenous cueing paradigm	Spatial and feature‐based task cue‐evoked activity	MNI	L	−42	11	25
						R	42	11	25
Muhle‐Karbe et al. (2014)	45 Task‐switching studies (*n* = 817)	N.A.	ALE fMRI meta‐analysis	Switch trials > Repetition trials	MNI	L	−40	4	32
Muhle‐Karbe et al. (2017)	23	24.09 ± 5.06	ALE fMRI meta‐analysis	Based on Derrfuss et al. (2005)	MNI (SPM8)	L	−40	4	30
						R	44	10	34
Nee and D'Esposito (2016)*	23	19.9; 18–28	Executive control paradigm	Main effects of temporal and contextual control and their interaction with feature control	MNI	L	−40	10	20
			
Sneve et al. (2013)*	6	21–28	Working memory paradigm	Anatomical definition and main effect of all visual stimuli	MNI	L	−44	3	33
						R	45	1	35
Sundermann and Pfleiderer (2012)	lIFJ: 180 experiments (*n* = 2274) rIFJ: 131 experiments (*n* = 1767)	N.A.	MACM fMRI meta‐analysis	Cuboid seeds adapted from the coordinates reported in Brass et al. (2005)	Talairach	L	−47	5	34
						R	47	5	34
Tamber‐Rosenau et al. (2018)*	10 (8 L, 9 R)	28.5 ± 3.3	Functional localizer	Prosaccades > Fixation	Talairach	L	−37.75	2.88	24.75
						R	40.67	1.22	25
Zanto et al. (2010)*	13	25; 20–31	Working memory paradigm (*n*‐back)	Functional connectivity attend colour/motion versus ignore colour/motion	MNI	L	−48	4	26
						R	42	0	26
Zanto et al. (2011)*	20	24.25; 18–31	Working memory paradigm (*n*‐back)	Functional connectivity attend colour/motion versus ignore colour/motion	MNI	L	−56	8	36
						R	44	20	24
Zhang et al. (2018)*	21	19–26	Feature‐based attention paradigm	Stimulus block effects	Talairach	L	−42	9	31
						R	44	11	29
Average coordinates*	13 studies (*n* = 202)		All paradigms	Covert and overt attention, working memory and cognitive control studies	MNI	L	−43.78 ± 4.88	7.80 ± 3.18	28.30 ± 5.59
	11 studies (*n* = 167)					R	44.96 ± 2.55	8.94 ± 5.92	27.21 ± 3.63

*Note*: We computed the average between all the IFJ coordinates reported from covert and overt attention, working memory and cognitive control tasks. The coordinates that were originally reported in Talairach space were converted to MNI space using the GingerALE utility convert foci (Eickhoff et al., 2012; Lancaster et al., 2007). In contrast to the frontal eye field (FEF), the variability in the average localization of the IFJ seems less pronounced, but it needs to be emphasized that most of the studies reviewed either implicitly or explicitly relied on an a priori anatomical description of this region or reported only the coordinates derived from second‐level analyses (see Sneve et al., 2013, Meyyappan et al., 2020, and Zanto et al., 2010, 2011, for examples, respectively). Thus, these factors make it difficult to provide an unbiased quantification of the genuine variability of the localization of the IFJ, which may be much higher and comparable with FEF variability.

Abbreviations: ALE, activation likelihood estimation; fMRI, functional magnetic resonance imaging; MACM, meta‐analytic connectivity modelling; N.A., not applicable.

Turning to the functional roles of the human FEF and IFJ, we have reported converging evidence from fMRI and M/EEG studies that the first is predominantly involved in coding spatial information, whereas the second is predominantly involved in coding non‐spatial information (feature and object information). While MVPA approaches have generally reported evidence supporting this claim (Jerde et al., 2012; Liu, 2016; Liu et al., 2011; Meyyappan et al., 2020; Muhle‐Karbe et al., 2017), we suggest that experimental manipulations that allow keeping sensory stimulation identical during the delay period but employ different cues (spatial vs. non‐spatial) can be effectively used to evaluate our current proposal within a single experimental design and in a variety of behavioural paradigms (e.g., see Meyyappan et al., 2020). We would like to stress that one could even push this logic further and suggest that if decoding analyses were to show an above‐chance performance when a classifier is trained on FEF activity when non‐spatial cues are presented in a purely feature‐ and object‐based paradigm (but not when trained on IFJ activity), and vice versa, an above‐chance performance when a classifier is trained on IFJ activity when spatial cues are presented (but not when trained on FEF activity), this would severely challenge our hypothesis regarding the functional specialization of the FEF and IFJ.

Another critical issue for evaluating the current proposal is to determine how exactly this information is coded by these regions at the computational level. In the working memory literature, an influential family of models emphasizes a distinction between regions that encode working memory information from other regions that dynamically exert control over these stored representations, possibly localized in the parietal and frontal cortices (reviewed in Serences, 2016; Sreenivasan et al., 2014). In the instance of the FEF and IFJ, are these regions only involved in the executive components of working memory, with the information being encoded at the distributed network level? If this isn't the case, at which level of abstraction are the sensory representations situated so they can guide attentional selection and influence working memory performance? We have reviewed studies that, thanks to the use of MVPA, have been able to decode attended and remembered information from both the FEF (Jerde et al., 2012) and the IFJ (Liu, 2016; Liu et al., 2011). This suggests that activity in these regions during the delay period doesn't merely reflect a control signal over other brain regions but actually contains some ongoing information processing related to the cue/memoranda itself. However, at present, it is unclear whether the FEF and IFJ encode this information at the same level of abstraction. Although both areas are positioned near the output layer of the cognitive system, based on the involvement of the IFJ in encoding stimulus‐response mappings according to novel task rules (Muhle‐Karbe et al., 2017), and in a variety of other high‐level cognitive operations (Assem et al., 2020; Brass et al., 2005), it could be argued that the IFJ may encode information at a higher level of abstraction compared with the FEF (reviewed in Section 3.3). This would be consistent with the relatively recent emergence of the IFJ along the evolutionary path of the primate species compared with the more ancient dorsal pathway's regions (Caminiti et al., 2015; Mars, Sotiropoulos, et al., 2018). We therefore suggest that this hypothesis should be investigated more systematically in future studies (see question three in Table 3). For example, one could design an *n*‐back fMRI experiment requiring either the encoding of the abstract identity of an object or its viewpoint and apply MVPA to the FEF and IFJ to see whether the decoding accuracy differs between the two in these tasks. We suggest that because the ‘identity’ task is more independent of low‐level visual features compared with the ‘viewpoint’ task, we should expect a better decoding accuracy within the IFJ compared with the FEF (see the study by Henderson & Serences, 2019, for interesting results that are in line with these predictions).

The PFC is generally characterized by a high degree of interconnectedness between its areas and with the rest of the brain—a property that is thought to underlie its unique contribution to complex behaviour (Fuster, 2001; Yeterian et al., 2012). This information aids to delineate the organization of the PFC along two principal axes: a rostro‐caudal (Koechlin et al., 2003) and a dorsoventral axis (Goldman‐Rakic, 1996). According to the results of the study by Nee and D'Esposito (2016), it is the mid‐lateral PFC that is positioned at the apex of the PFC hierarchy, based on the asymmetries in its effective connectivity patterns. In their model, the FEF and IFJ show sensitivity to the stimulus context and sensory domain (spatial vs. verbal) and are hence hypothesized to be under the control of the cMFG and IFS, respectively (Nee & D'Esposito, 2016). In an updated version of this hierarchical model of cognitive control, Badre and Nee (2018) place the FEF and IFJ in the sensory‐motor control areas that allow the maintenance of goal‐related information to control movement in a domain‐specific way; thus, although they are embedded in different information processing streams, they seem to be localized within the same gradient of the rostro‐caudal axis. The authors also hypothesize that lateral areas could be similarly influenced by motivational factors that are propagated from the dorsomedial PFC at a comparable gradient in the PFC hierarchy (Badre & Nee, 2018). This suggestion parallels developments from the last decade in cognitive models of attention, which began to incorporate distinct forms of attentional biases stemming from learning processes and motivational factors (Anderson, 2019; Awh et al., 2012; Macaluso & Doricchi, 2013). It is however still unknown how these signals shape activity in the PFC (a possibility that we suggest should be investigated in future studies), and how the FEF and IFJ dynamically interact with subcortical and thalamic structures to achieve efficient control of behaviour (Halassa & Kastner, 2017; White et al., 2017).

Finally, we would like to highlight some of the most promising future research directions that would allow dissociating the function of the FEF and IFJ. As we have argued throughout this review, the concept of connectivity fingerprints (Mars, Passingham, & Jbabdi, 2018; Passingham et al., 2002) provides a useful framework to tackle this problem. If we assume that the FEF and IFJ occupy a dissociable functional role in the brain networks underlying visual attention, visual working memory and cognitive control, this leads to the prediction that this differentiation could be reflected in their connectivity fingerprints. If we build upon the difference of the representational format they encode, this contrast might allow for the individuation of a spatial and a non‐spatial network based on their structural and functional connectivity with parietal, temporal and early visual cortices (see questions four and five in Table 3). More specifically, this hypothesis would entail predominant structural and functional connectivity with topographic visual areas (Wang et al., 2015) from the FEF, and vice versa, predominant structural and functional connectivity of the IFJ with areas that are involved in central vision, coding for visual feature and object representations (Kravitz et al., 2013). Another interesting hypothesis would be to investigate whether the parietal cortex contains regions to which the associative fibres from the plPFC send segregated projections, as well as regions in which they converge, therefore possibly enabling the communication between these two processing streams, as it would be predicted, for example, by neural models of priority maps (Fecteau & Munoz, 2006; Zelinsky & Bisley, 2015). As we mentioned in Section 4.1, the limited evidence on the structural connectivity patterns of the FEF, which is even more pronounced in the case of the IFJ, is quite astonishing given the increasing number of publications that investigated these regions. Nevertheless, the existent data make both these hypotheses plausible, and their likelihood is further strengthened in the light of comparative evidence (Caminiti et al., 2015; Croxson et al., 2005; de Schotten et al., 2011; Felleman & Van Essen, 1991; Kravitz et al., 2013; Yeterian et al., 2012).

In contrast, studies on resting‐state fMRI allowed gathering an impressive amount of information about the involvement of the FEF and IFJ in specific brain networks (Ji et al., 2019; Yeo et al., 2011). Despite their power to encapsulate functional activity at the whole‐brain level, it is still unknown how well these networks offer a good model for the co‐activation patterns that are measured using experimental manipulations in task fMRI (however, see Tavor et al., 2016). In particular, in the case of the FEF and IFJ, their assignment to these network parcellations likely represents a convenient way to operate a dimensionality reduction on their activity patterns (and their hypothesized functions), but at the cost of losing track of their multiple and varied co‐activation patterns. Therefore, we suggest that techniques such as MACM (Robinson et al., 2010; reviewed in Section 4.3) should be regarded as the most relevant source of evidence when attempting to dissociate the FEF and IFJ based on their functional connectivity patterns.

Top‐down selection and working memory maintenance typically involve a complex chain of neural activity patterns, and this dynamic nature cannot be underestimated. Tracking the temporal profile of this activity at an appropriate timescale is therefore crucial to understand selective visual behaviour (Fiebelkorn & Kastner, 2019). Due to the limited temporal resolution, fMRI is not suited to resolve the complete role of the FEF and IFJ at different stages of information processing, the transient versus sustained nature of their activity and the interplay of spectral activity within the attention networks. Another outstanding issue pertains to the role of rhythmic activity in specific frequency bands, which is a distinctive signature of many perceptual, attentional and working memory processes (Buschman & Miller, 2007, 2009; Fiebelkorn & Kastner, 2019; Fries, 2005; VanRullen, 2016). Electrophysiological methods (M/EEG, electrocorticography) should be able to close this gap by providing accurate measures of FEF and IFJ activity as it unfolds over time (Baldauf & Desimone, 2014; Chacko et al., 2018; Martin et al., 2019; Michalareas et al., 2016; Popov et al., 2017; Szczepanski et al., 2014; Tabarelli et al., 2020). Furthermore, the analysis of functional and effective connectivity metrics should allow modelling the reciprocal links between FEF and IFJ activities. As representative examples of this approach, the studies by Sneve et al. (2013), Baldauf and Desimone (2014) and Zhang et al. (2018) contributed in a foundational way to show that functional and effective connectivity metrics can be used to identify the sources of the attentional biases observed in feature‐ and object‐based attention and in working memory tasks. More specifically, their studies together suggest that the IFJ may be responsible for modulating activity elsewhere to support non‐spatial selection and working memory encoding. The role of the FEF is in contrast to that of a spatial priority map (Fecteau & Munoz, 2006; Itti & Koch, 2001; Thompson & Bichot, 2005), which likely binds non‐spatial and spatial information and maps it to an overt response. The careful combination of these analytical techniques will likely lead in the future to uncover how task information is transferred between the FEF and IFJ and posterior regions. Concerning this question, recently, it has been proposed that informational connectivity could be used to investigate directed information transfer between brain regions (Anzellotti & Coutanche, 2018) by modelling how well activity patterns in a brain region can help to predict activity in another region. This multivariate technique preserves the information contained in all the voxels, overcoming the issues associated with univariate approaches (i.e., averaging), hence being optimally adept to uncover mechanisms that are not apparent even with the application of MVPA to single brain regions. Another great advantage of this technique is that the experimental conditions/stimuli classes can be carefully chosen to determine which information content is driving the informational connectivity between specific regions (Anzellotti & Coutanche, 2018), which we suggest is a feature that can be clearly leveraged to investigate our hypothesis of spatial and non‐spatial control from the FEF and IFJ to posterior regions.

Finally, arguably one of the most direct ways to dissociate the contributions of the FEF and IFJ to selective visual behaviour would be to perform a TMS experiment using an endogenous cueing paradigm alternating spatial and feature‐based attention blocks (see question six in Table 3). During the delay period, the experimenter would administer repetitive pulses of TMS to the FEF and the IFJ in each block and assess their effects on behavior. Our main prediction would be to observe a dissociation in the effects of the stimulation that is specific to each region and which interacts with the type of cue presented within a block (e.g., spatial vs. colour cue). Because most of the studies reviewed above point towards the right lateralization of the attention networks, the proposed study could initially focus on stimulating the FEF and IFJ in the right hemisphere.

Visual attention and working memory play a fundamental role in our increasingly cluttered and distracting environments. Understanding the neural mechanisms and the brain networks underlying these functions is crucial for cognitive neuroscience, but it is ultimately also a relevant goal for clinical and industrial applications that could greatly benefit from this basic knowledge. By contrasting the structure, function and connectivity fingerprints of the FEF and IFJ, we have provided some insights into how comparative research and multimodal neuroimaging data can be fruitfully combined to study the organization of the plPFC and to dissociate the function and connectivity fingerprints of the FEF and IFJ as we continue to investigate the neural mechanisms of selective goal‐driven behaviour.

## CONFLICT OF INTEREST

The authors report no conflict of interest.

## AUTHOR CONTRIBUTIONS

MB conceptualized the organization of the review, collected the references, wrote the initial draft and prepared the figures and tables. DB supervised the writing of the manuscript and the preparation of the figures and tables, revised them and suggested additional references. Both authors contributed to the general discussion by proposing avenues for future research. Finally, they both revised and agreed on the final version of the manuscript.

### PEER REVIEW

The peer review history for this article is available at https://publons.com/publon/10.1111/ejn.15393.

## Data Availability

Data sharing is not applicable to this article as no new data were created or analysed in this study.

## References

[ejn15393-bib-0001] Abdollahi, R. O. , Kolster, H. , Glasser, M. F. , Robinson, E. C. , Coalson, T. S. , Dierker, D. , Jenkinson, M. , Van Essen, D. C. , & Orban, G. A. (2014). Correspondences between retinotopic areas and myelin maps in human visual cortex. NeuroImage, 99, 509–524. 10.1016/j.neuroimage.2014.06.042 24971513 PMC4121090

[ejn15393-bib-0002] Amiez, C. , Kostopoulos, P. , Champod, A. S. , & Petrides, M. (2006). Local morphology predicts functional organization of the dorsal premotor region in the human brain. Journal of Neuroscience, 26(10), 2724–2731. 10.1523/JNEUROSCI.4739-05.2006 16525051 PMC6675158

[ejn15393-bib-0003] Amiez, C. , & Petrides, M. (2009). Anatomical organization of the eye fields in the human and non‐human primate frontal cortex. Progress in Neurobiology, 89(2), 220–230. 10.1016/j.pneurobio.2009.07.010 19665515

[ejn15393-bib-0004] Amunts, K. , Lenzen, M. , Friederici, A. D. , Schleicher, A. , Morosan, P. , Palomero‐Gallagher, N. , & Zilles, K. (2010). Broca's region: Novel organizational principles and Multiple Receptor Mapping. PLoS Biology, 8(9), e1000489. 10.1371/journal.pbio.1000489 20877713 PMC2943440

[ejn15393-bib-0005] Amunts, K. , Mohlberg, H. , Bludau, S. , & Zilles, K. (2020). Julich‐Brain: A 3D probabilistic atlas of the human brain's cytoarchitecture. Science, 369(6506), 988–992. 10.1126/science.abb4588 32732281

[ejn15393-bib-0006] Amunts, K. , Schleicher, A. , Bürgel, U. , Mohlberg, H. , Uylings, H. B. , & Zilles, K. (1999). Broca's region revisited: Cytoarchitecture and intersubject variability. Journal of Comparative Neurology, 412(2), 319–341. 10.1002/(SICI)1096-9861(19990920)412:2<319::AID-CNE10>3.0.CO;2-7 10441759

[ejn15393-bib-0007] Amunts, K. , & von Cramon, D. Y. (2006). The anatomical segregation of the frontal cortex: What does it mean for function? Cortex, 42(4), 525–528. 10.1016/S0010-9452(08)70392-7 16881264

[ejn15393-bib-0008] Amunts, K. , & Zilles, K. (2015). Architectonic mapping of the human brain beyond Brodmann. Neuron, 88(6), 1086–1107. 10.1016/j.neuron.2015.12.001 26687219

[ejn15393-bib-0009] Anderson, B. A. (2019). Neurobiology of value‐driven attention. Current Opinion in Psychology, 29, 27–33. 10.1016/j.copsyc.2018.11.004 30472540

[ejn15393-bib-0010] Anderson, E. J. , Jones, D. K. , O'Gorman, R. L. , Leemans, A. , Catani, M. , & Husain, M. (2012). Cortical network for gaze control in humans revealed using multimodal MRI. Cerebral Cortex, 22(4), 765–775. 10.1093/cercor/bhr110 21693784 PMC3306571

[ejn15393-bib-0011] Anzellotti, S. , & Coutanche, M. N. (2018). Beyond functional connectivity: Investigating networks of multivariate representations. Trends in Cognitive Sciences, 22(3), 258–269. 10.1016/j.tics.2017.12.002 29305206

[ejn15393-bib-0012] Asplund, C. L. , Todd, J. J. , Snyder, A. P. , & Marois, R. (2010). A central role for the lateral prefrontal cortex in goal‐directed and stimulus‐driven attention. Nature Neuroscience, 13(4), 507–512. 10.1038/nn.2509 20208526 PMC2847024

[ejn15393-bib-0013] Assem, M. , Glasser, M. F. , Van Essen, D. C. , & Duncan, J. (2020). A domain‐general cognitive core defined in multimodally parcellated human cortex. Cerebral Cortex, 30(8), 4361–4380. 10.1093/cercor/bhaa023 32244253 PMC7325801

[ejn15393-bib-0014] Autio, J. A. , Glasser, M. F. , Ose, T. , Donahue, C. J. , Bastiani, M. , Ohno, M. , … Yamaguchi, M. (2020). Towards HCP‐Style macaque connectomes: 24‐Channel 3T multi‐array coil, MRI sequences and preprocessing. NeuroImage, 215, 116800. 10.1016/j.neuroimage.2020.116800 32276072 PMC7116593

[ejn15393-bib-0015] Awh, E. , Belopolsky, A. V. , & Theeuwes, J. (2012). Top‐down versus bottom‐up attentional control: A failed theoretical dichotomy. Trends in Cognitive Sciences, 16(8), 437–443. 10.1016/j.tics.2012.06.010 22795563 PMC3426354

[ejn15393-bib-0016] Awh, E. , & Jonides, J. (2001). Overlapping mechanisms of attention and spatial working memory. Trends in Cognitive Sciences, 5(3), 119–126. 10.1016/S1364-6613(00)01593-X 11239812

[ejn15393-bib-0017] Awh, E. , Jonides, J. , & Reuter‐Lorenz, P. A. (1998). Rehearsal in spatial working memory. Journal of Experimental Psychology: Human Perception and Performance, 24(3), 780–790. 10.1037/0096-1523.24.3.780 9627416

[ejn15393-bib-0018] Baddeley, A. D. (1993). Working memory or working attention? In A. Baddeley & L. Weiskrantz (Eds.), Attention: Selection, awareness, and control (pp. 152–170). Clarendon.

[ejn15393-bib-0019] Badre, D. , & Nee, D. E. (2018). Frontal cortex and the hierarchical control of behavior. Trends in Cognitive Sciences, 22(2), 170–188. 10.1016/j.tics.2017.11.005 29229206 PMC5841250

[ejn15393-bib-0020] Baldauf, D. , & Desimone, R. (2014). Neural mechanisms of object‐based attention. Science, 344(6182), 424–427. 10.1126/science.1247003 24763592

[ejn15393-bib-0021] Barbas, H. , & Pandya, D. N. (1989). Architecture and intrinsic connections of the prefrontal cortex in the rhesus monkey. Journal of Comparative Neurology, 286(3), 353–375. 10.1002/cne.902860306 2768563

[ejn15393-bib-0022] Barceló, F. , Suwazono, S. , & Knight, R. T. (2000). Prefrontal modulation of visual processing in humans. Nature Neuroscience, 3(4), 399–403. 10.1038/73975 10725931

[ejn15393-bib-0023] Bardi, L. , Kanai, R. , Mapelli, D. , & Walsh, V. (2012). TMS of the FEF interferes with spatial conflict. Journal of Cognitive Neuroscience, 24(6), 1305–1313. 10.1162/jocn_a_00223 22401287

[ejn15393-bib-0024] Behrens, T. E. , Woolrich, M. W. , Jenkinson, M. , Johansen‐Berg, H. , Nunes, R. G. , Clare, S. , … Smith, S. M. (2003). Characterization and propagation of uncertainty in diffusion‐weighted MR imaging. Magnetic Resonance in Medicine: An Official Journal of the International Society for Magnetic Resonance in Medicine, 50(5), 1077–1088. 10.1002/mrm.10609 14587019

[ejn15393-bib-0025] Benson, N. C. , Jamison, K. W. , Arcaro, M. J. , Vu, A. T. , Glasser, M. F. , Coalson, T. S. , Van Essen, D. C. , Yacoub, E. , Ugurbil, K. , Winawer, J. , & Kay, K. (2018). The Human Connectome Project 7 Tesla retinotopy dataset: Description and population receptive field analysis. Journal of Vision, 18(13), 1–22. 10.1167/18.13.23 PMC6314247 30593068

[ejn15393-bib-0026] Bichot, N. P. , Heard, M. T. , DeGennaro, E. M. , & Desimone, R. (2015). A source for feature‐based attention in the prefrontal cortex. Neuron, 88(4), 832–844. 10.1016/j.neuron.2015.10.001 26526392 PMC4655197

[ejn15393-bib-0027] Bichot, N. P. , Xu, R. , Ghadooshahy, A. , Williams, M. L. , & Desimone, R. (2019). The role of prefrontal cortex in the control of feature attention in area V4. Nature Communications, 10(1), 1–12. 10.1038/s41467-019-13761-7 PMC6915702 31844117

[ejn15393-bib-0028] Brass, M. , Derrfuss, J. , Forstmann, B. , & von Cramon, D. Y. (2005). The role of the inferior frontal junction area in cognitive control. Trends in Cognitive Sciences, 9(7), 314–316. 10.1016/j.tics.2005.05.001 15927520

[ejn15393-bib-0029] Brass, M. , & von Cramon, D. Y. (2002). The role of the frontal cortex in task preparation. Cerebral Cortex, 12(9), 908–914. 10.1093/cercor/12.9.908 12183390

[ejn15393-bib-0030] Brass, M. , & von Cramon, D. Y. (2004). Decomposing components of task preparation with functional magnetic resonance imaging. Journal of Cognitive Neuroscience, 16(4), 609–620. 10.1162/089892904323057335 15165351

[ejn15393-bib-0031] Bristow, D. , Haynes, J. D. , Sylvester, R. , Frith, C. D. , & Rees, G. (2005). Blinking suppresses the neural response to unchanging retinal stimulation. Current Biology, 15(14), 1296–1300. 10.1016/j.cub.2005.06.025 16051173

[ejn15393-bib-0032] Brodmann, K. (1909). Vergleichende Lokalisationslehre der Grosshirnrinde in ihren Prinzipien dargestellt auf Grund des Zellenbaues. Barth.

[ejn15393-bib-0033] Bruce, C. J. , Goldberg, M. E. , Bushnell, M. C. , & Stanton, G. B. (1985). Primate frontal eye fields. II. Physiological and anatomical correlates of electrically evoked eye movements. Journal of Neurophysiology, 54(3), 714–734. 10.1152/jn.1985.54.3.714 4045546

[ejn15393-bib-0034] Bullier, J. (2001). Integrated model of visual processing. Brain Research Reviews, 36(2–3), 96–107. 10.1016/S0165-0173(01)00085-6 11690606

[ejn15393-bib-0035] Bunge, S. A. , Kahn, I. , Wallis, J. D. , Miller, E. K. , & Wagner, A. D. (2003). Neural circuits subserving the retrieval and maintenance of abstract rules. Journal of Neurophysiology, 90(5), 3419–3428. 10.1152/jn.00910.2002 12867532

[ejn15393-bib-0036] Buschman, T. J. , & Miller, E. K. (2007). Top‐down versus bottom‐up control of attention in the prefrontal and posterior parietal cortices. Science, 315(5820), 1860–1862. 10.1126/science.1138071 17395832

[ejn15393-bib-0037] Buschman, T. J. , & Miller, E. K. (2009). Serial, covert shifts of attention during visual search are reflected by the frontal eye fields and correlated with population oscillations. Neuron, 63(3), 386–396. 10.1016/j.neuron.2009.06.020 19679077 PMC2758537

[ejn15393-bib-0038] Caminiti, R. , Innocenti, G. M. , & Battaglia‐Mayer, A. (2015). Organization and evolution of parieto‐frontal processing streams in macaque monkeys and humans. Neuroscience & Biobehavioral Reviews, 56, 73–96. 10.1016/j.neubiorev.2015.06.014 26112130

[ejn15393-bib-0039] Carrasco, M. (2011). Visual attention: The past 25 years. Vision Research, 51(13), 1484–1525. 10.1016/j.visres.2011.04.012 21549742 PMC3390154

[ejn15393-bib-0040] Chacko, R. V. , Kim, B. , Jung, S. W. , Daitch, A. L. , Roland, J. L. , Metcalf, N. V. , … Leuthardt, E. C. (2018). Distinct phase‐amplitude couplings distinguish cognitive processes in human attention. NeuroImage, 175, 111–121. 10.1016/j.neuroimage.2018.03.003 29518565 PMC6369928

[ejn15393-bib-0041] Chan, A. W. Y. (2013). Functional organization and visual representations of human ventral lateral prefrontal cortex. Frontiers in Psychology, 4, 371. 10.3389/fpsyg.2013.00371 23847558 PMC3705197

[ejn15393-bib-0042] Chun, M. M. , Golomb, J. D. , & Turk‐Browne, N. B. (2011). A taxonomy of external and internal attention. Annual Review of Psychology, 62, 73–101. 10.1146/annurev.psych.093008.100427 19575619

[ejn15393-bib-0043] Cieslik, E. , Seidler, I. , Laird, A. , Fox, P. , & Eickhoff, S. (2016). Different involvement of subregions within dorsal premotor and medial frontal cortex for pro‐and antisaccades. Neuroscience and Biobehavioral Reviews, 68, 256–269. 10.1016/j.neubiorev.2016.05.012 27211526 PMC5003685

[ejn15393-bib-0044] Cole, M. W. , Reynolds, J. R. , Power, J. D. , Repovs, G. , Anticevic, A. , & Braver, T. S. (2013). Multi‐task connectivity reveals flexible hubs for adaptive task control. Nature Neuroscience, 16(9), 1348–1355. 10.1038/nn.3470 23892552 PMC3758404

[ejn15393-bib-0045] Cole, M. W. , & Schneider, W. (2007). The cognitive control network: Integrated cortical regions with dissociable functions. NeuroImage, 37(1), 343–360. 10.1016/j.neuroimage.2007.03.071 17553704

[ejn15393-bib-0046] Constantinidis, C. , & Qi, X. L. (2018). Representation of spatial and feature information in the monkey dorsal and ventral prefrontal cortex. Frontiers in Integrative Neuroscience, 12, 31. 10.3389/fnint.2018.00031 30131679 PMC6090048

[ejn15393-bib-0047] Corbetta, M. (1998). Frontoparietal cortical networks for directing attention and the eye to visual locations: Identical, independent, or overlapping neural systems? Proceedings of the National Academy of Sciences, 95(3), 831–838. 10.1073/pnas.95.3.831 PMC33805 9448248

[ejn15393-bib-0048] Corbetta, M. , Akbudak, E. , Conturo, T. E. , Snyder, A. Z. , Ollinger, J. M. , Drury, H. A. , … Shulman, G. L. (1998). A common network of functional areas for attention and eye movements. Neuron, 21(4), 761–773. 10.1016/S0896-6273(00)80593-0 9808463

[ejn15393-bib-0049] Corbetta, M. , & Shulman, G. L. (2002). Control of goal‐directed and stimulus‐driven attention in the brain. Nature Reviews Neuroscience, 3(3), 201–215. 10.1038/nrn755 11994752

[ejn15393-bib-0050] Courtney, S. M. , Petit, L. , Maisog, J. M. , Ungerleider, L. G. , & Haxby, J. V. (1998). An area specialized for spatial working memory in human frontal cortex. Science, 279(5355), 1347–1351. 10.1126/science.279.5355.1347 9478894

[ejn15393-bib-0051] Croxson, P. L. , Johansen‐Berg, H. , Behrens, T. E. , Robson, M. D. , Pinsk, M. A. , Gross, C. G. , … Rushworth, M. F. (2005). Quantitative investigation of connections of the prefrontal cortex in the human and macaque using probabilistic diffusion tractography. Journal of Neuroscience, 25(39), 8854–8866. 10.1523/JNEUROSCI.1311-05.2005 16192375 PMC6725599

[ejn15393-bib-0052] De Pasquale, F. , Della Penna, S. , Snyder, A. Z. , Lewis, C. , Mantini, D. , Marzetti, L. , … Corbetta, M. (2010). Temporal dynamics of spontaneous MEG activity in brain networks. Proceedings of the National Academy of Sciences, 107(13), 6040–6045. 10.1073/pnas.0913863107 PMC2851876 20304792

[ejn15393-bib-0053] de Schotten, M. T. , Dell'Acqua, F. , Forkel, S. J. , Simmons, A. , Vergani, F. , Murphy, D. G. , & Catani, M. (2011). A lateralized brain network for visuospatial attention. Nature Neuroscience, 14(10), 1245–1247. 10.1038/npre.2011.5549.1 21926985

[ejn15393-bib-0054] Dell'Acqua, F. , Scifo, P. , Rizzo, G. , Catani, M. , Simmons, A. , Scotti, G. , & Fazio, F. (2010). A modified damped Richardson–Lucy algorithm to reduce isotropic background effects in spherical deconvolution. NeuroImage, 49(2), 1446–1458. 10.1016/j.neuroimage.2009.09.033 19781650

[ejn15393-bib-0055] Derrfuss, J. , Brass, M. , Neumann, J. , & von Cramon, D. Y. (2005). Involvement of the inferior frontal junction in cognitive control: Meta‐analyses of switching and Stroop studies. Human Brain Mapping, 25(1), 22–34. 10.1002/hbm.20127 15846824 PMC6871679

[ejn15393-bib-0056] Derrfuss, J. , Brass, M. , & Von Cramon, D. Y. (2004). Cognitive control in the posterior frontolateral cortex: Evidence from common activations in task coordination, interference control, and working memory. NeuroImage, 23(2), 604–612. 10.1016/j.neuroimage.2004.06.007 15488410

[ejn15393-bib-0057] Derrfuss, J. , Brass, M. , von Cramon, D. Y. , Lohmann, G. , & Amunts, K. (2009). Neural activations at the junction of the inferior frontal sulcus and the inferior precentral sulcus: Interindividual variability, reliability, and association with sulcal morphology. Human Brain Mapping, 30(1), 299–311. 10.1002/hbm.20501 18072280 PMC6870901

[ejn15393-bib-0058] Derrfuss, J. , Vogt, V. L. , Fiebach, C. J. , Von Cramon, D. Y. , & Tittgemeyer, M. (2012). Functional organization of the left inferior precentral sulcus: Dissociating the inferior frontal eye field and the inferior frontal junction. NeuroImage, 59(4), 3829–3837. 10.1016/j.neuroimage.2011.11.051 22155041

[ejn15393-bib-0059] Desimone, R. , & Duncan, J. (1995). Neural mechanisms of selective visual attention. Annual Review of Neuroscience, 18(1), 193–222. 10.1146/annurev.ne.18.030195.001205 7605061

[ejn15393-bib-0060] Deubel, H. , & Schneider, W. X. (1996). Saccade target selection and object recognition: Evidence for a common attentional mechanism. Vision Research, 36(12), 1827–1838. 10.1016/0042-6989(95)00294-4 8759451

[ejn15393-bib-0061] Donahue, C. J. , Glasser, M. F. , Preuss, T. M. , Rilling, J. K. , & Van Essen, D. C. (2018). Quantitative assessment of prefrontal cortex in humans relative to nonhuman primates. Proceedings of the National Academy of Sciences of the United States of America, 115(22), E5183–E5192. 10.1073/pnas.1721653115 29739891 PMC5984508

[ejn15393-bib-0062] Donahue, C. J. , Sotiropoulos, S. N. , Jbabdi, S. , Hernandez‐Fernandez, M. , Behrens, T. E. , Dyrby, T. B. , … Glasser, M. F. (2016). Using diffusion tractography to predict cortical connection strength and distance: A quantitative comparison with tracers in the monkey. Journal of Neuroscience, 36(25), 6758–6770. 10.1523/JNEUROSCI.0493-16.2016 27335406 PMC4916250

[ejn15393-bib-0063] Dumoulin, S. O. , & Wandell, B. A. (2008). Population receptive field estimates in human visual cortex. NeuroImage, 39(2), 647–660. 10.1016/j.neuroimage.2007.09.034 17977024 PMC3073038

[ejn15393-bib-0064] Duncan, J. (2001). An adaptive coding model of neural function in prefrontal cortex. Nature Reviews Neuroscience, 2(11), 820–829. 10.1038/35097575 11715058

[ejn15393-bib-0065] Duncan, J. (2010). The multiple‐demand (MD) system of the primate brain: Mental programs for intelligent behaviour. Trends in Cognitive Sciences, 14(4), 172–179. 10.1016/j.tics.2010.01.004 20171926

[ejn15393-bib-0066] Dux, P. E. , Ivanoff, J. , Asplund, C. L. , & Marois, R. (2006). Isolation of a central bottleneck of information processing with time‐resolved fMRI. Neuron, 52(6), 1109–1120. 10.1016/j.neuron.2006.11.009 17178412 PMC2527865

[ejn15393-bib-0067] Eichert, N. , Robinson, E. C. , Bryant, K. L. , Jbabdi, S. , Jenkinson, M. , Li, L. , … Mars, R. B. (2020). Cross‐species cortical alignment identifies different types of anatomical reorganization in the primate temporal lobe. eLife, 9, e53232. 10.7554/eLife.53232.sa2 32202497 PMC7180052

[ejn15393-bib-0068] Eickhoff, S. B. , Bzdok, D. , Laird, A. R. , Kurth, F. , & Fox, P. T. (2012). Activation likelihood estimation meta‐analysis revisited. NeuroImage, 59(3), 2349–2361. 10.1016/j.neuroimage.2011.09.017 21963913 PMC3254820

[ejn15393-bib-0069] Eickhoff, S. B. , Constable, R. T. , & Yeo, B. T. (2018). Topographic organization of the cerebral cortex and brain cartography. NeuroImage, 170, 332–347. 10.1016/j.neuroimage.2017.02.018 28219775 PMC5563483

[ejn15393-bib-0070] Eickhoff, S. B. , Yeo, B. T. T. , & Genon, S. (2018). Imaging‐based parcellations of the human brain. Nature Reviews Neuroscience, 19(11), 672–686. 10.1038/s41583-018-0071-7 30305712

[ejn15393-bib-0071] Fecteau, J. H. , & Munoz, D. P. (2006). Salience, relevance, and firing: A priority map for target selection. Trends in Cognitive Sciences, 10(8), 382–390. 10.1016/j.tics.2006.06.011 16843702

[ejn15393-bib-0072] Fedorenko, E. , Duncan, J. , & Kanwisher, N. (2012). Language‐selective and domain‐general regions lie side by side within Broca's area. Current Biology, 22(21), 2059–2062. 10.1016/j.cub.2012.09.011 23063434 PMC3494832

[ejn15393-bib-0073] Fedorenko, E. , Duncan, J. , & Kanwisher, N. (2013). Broad domain generality in focal regions of frontal and parietal cortex. Proceedings of the National Academy of Sciences, 110(41), 16616–16621. 10.1073/pnas.1315235110 PMC3799302 24062451

[ejn15393-bib-0074] Felleman, D. J. , & Van Essen, D. C. (1991). Distributed hierarchical processing in the primate cerebral cortex. Cerebral Cortex, 1(1), 1–47. 10.1093/cercor/1.1.1 1822724

[ejn15393-bib-0075] Ferrier, D. (1875). Experiments on the brain of monkeys. No. I. Proceedings of the Royal Society of London, 23(156‐163), 409–430. 10.1098/rspl.1874.0058

[ejn15393-bib-0076] Fiebelkorn, I. C. , & Kastner, S. (2019). A rhythmic theory of attention. Trends in Cognitive Sciences, 23(2), 87–101. 10.1016/j.tics.2018.11.009 30591373 PMC6343831

[ejn15393-bib-0077] Fiebelkorn, I. C. , & Kastner, S. (2020). Functional specialization in the attention network. Annual Review of Psychology, 71, 221–249. 10.1146/annurev-psych-010418-103429 PMC7026883 31514578

[ejn15393-bib-0078] Foerster, O. (1931). The cerebral cortex in man. Lancet, 2, 309–312. 10.1016/S0140-6736(00)47063-7

[ejn15393-bib-0079] Fox, M. D. , Corbetta, M. , Snyder, A. Z. , Vincent, J. L. , & Raichle, M. E. (2006). Spontaneous neuronal activity distinguishes human dorsal and ventral attention systems. Proceedings of the National Academy of Sciences, 103(26), 10046–10051. 10.1073/pnas.0604187103 PMC1480402 16788060

[ejn15393-bib-0080] Fries, P. (2005). A mechanism for cognitive dynamics: Neuronal communication through neuronal coherence. Trends in Cognitive Sciences, 9(10), 474–480. 10.1016/j.neuron.2015.09.034 16150631

[ejn15393-bib-0081] Funahashi, S. , Bruce, C. J. , & Goldman‐Rakic, P. S. (1989). Mnemonic coding of visual space in the monkey's dorsolateral prefrontal cortex. Journal of Neurophysiology, 61(2), 331–349. 10.1152/jn.1989.61.2.331 2918358

[ejn15393-bib-0082] Fuster, J. M. (2001). The prefrontal cortex—An update: Time is of the essence. Neuron, 30(2), 319–333. 10.1016/s0896-6273(01)00285-9 11394996

[ejn15393-bib-0083] Fuster, J. M. , & Alexander, G. E. (1971). Neuron activity related to short‐term memory. Science, 173(3997), 652–654. 10.1126/science.173.3997.652 4998337

[ejn15393-bib-0084] Gaspelin, N. , & Luck, S. J. (2018). The role of inhibition in avoiding distraction by salient stimuli. Trends in Cognitive Sciences, 22(1), 79–92. 10.1016/j.tics.2017.11.001 29191511 PMC5742040

[ejn15393-bib-0085] Gazzaley, A. , & Nobre, A. C. (2012). Top‐down modulation: Bridging selective attention and working memory. Trends in Cognitive Sciences, 16(2), 129–135. 10.1016/j.tics.2011.11.014 22209601 PMC3510782

[ejn15393-bib-0086] Germann, J. , & Petrides, M. (2020). The ventral part of dorsolateral frontal area 8A regulates visual attentional selection and the dorsal part auditory attentional selection. Neuroscience, 441, 209–216. 10.1016/j.neuroscience.2020.05.057 32512135

[ejn15393-bib-0087] Giesbrecht, B. , Woldorff, M. G. , Song, A. W. , & Mangun, G. R. (2003). Neural mechanisms of top‐down control during spatial and feature attention. NeuroImage, 19(3), 496–512. 10.1016/S1053-8119(03)00162-9 12880783

[ejn15393-bib-0088] Glasser, M. F. , Coalson, T. S. , Robinson, E. C. , Hacker, C. D. , Harwell, J. , Yacoub, E. , Ugurbil, K. , Andersson, J. , Beckmann, C. F. , Jenkinson, M. , Smith, S. M. , & Van Essen, D. C. (2016). A multi‐modal parcellation of human cerebral cortex. Nature, 536(7615), 171–178. 10.1038/nature18933 27437579 PMC4990127

[ejn15393-bib-0089] Goldman‐Rakic, P. S. (1996). The prefrontal landscape: Implications of functional architecture for understanding human mentation and the central executive. Philosophical Transactions of the Royal Society of London. Series B: Biological Sciences, 351(1346), 1445–1453. 10.1098/rstb.1996.0129 8941956

[ejn15393-bib-0090] Gong, M. , & Liu, T. (2020). Biased neural representation of feature‐based attention in the human frontoparietal network. Journal of Neuroscience, 40(43), 8386–8395. 10.1523/JNEUROSCI.0690-20.2020 33004380 PMC7577593

[ejn15393-bib-0091] Goodale, M. A. , & Milner, A. D. (1992). Separate visual pathways for perception and action. Trends in Neurosciences, 15(1), 20–25. 10.1016/0166-2236(92)90344-8 1374953

[ejn15393-bib-0092] Greenberg, A. S. , Esterman, M. , Wilson, D. , Serences, J. T. , & Yantis, S. (2010). Control of spatial and feature‐based attention in frontoparietal cortex. Journal of Neuroscience, 30(43), 14330–14339. 10.1523/JNEUROSCI.4248-09.2010 20980588 PMC3307052

[ejn15393-bib-0093] Gregoriou, G. G. , Gotts, S. J. , Zhou, H. , & Desimone, R. (2009). High‐frequency, long‐range coupling between prefrontal and visual cortex during attention. Science, 324(5931), 1207–1210. 10.1126/science.1171402 19478185 PMC2849291

[ejn15393-bib-0094] Gross, J. (2019). Magnetoencephalography in cognitive neuroscience: A primer. Neuron, 104(2), 189–204. 10.1016/j.neuron.2019.07.001 31647893

[ejn15393-bib-0095] Hagler, D. J. , & Sereno, M. I. (2006). Spatial maps in frontal and prefrontal cortex. NeuroImage, 29(2), 567–577. 10.1016/j.neuroimage.2005.08.058 16289928

[ejn15393-bib-0096] Halassa, M. M. , & Kastner, S. (2017). Thalamic functions in distributed cognitive control. Nature Neuroscience, 20(12), 1669–1679. 10.1038/s41593-017-0020-1 29184210

[ejn15393-bib-0097] Han, S. W. , & Marois, R. (2014). Functional fractionation of the stimulus‐driven attention network. Journal of Neuroscience, 34(20), 6958–6969. 10.1523/JNEUROSCI.4975-13.2014 24828649 PMC4099520

[ejn15393-bib-0098] Haxby, J. V. , Gobbini, M. I. , Furey, M. L. , Ishai, A. , Schouten, J. L. , & Pietrini, P. (2001). Distributed and overlapping representations of faces and objects in ventral temporal cortex. Science, 293(5539), 2425–2430. 10.1126/science.1063736 11577229

[ejn15393-bib-0099] Hayashi, T. , Hou, Y. , Glasser, M. F. , Autio, J. A. , Knoblauch, K. , Inoue‐Murayama, M. , … Van Essen, D. C. (2020). The nonhuman primate neuroimaging and neuroanatomy project. NeuroImage. 10.1016/j.neuroimage.2021.117726 PMC8079967 33484849

[ejn15393-bib-0247] He B. J. , Snyder A. Z. , Vincent J. L. , Epstein A. , Shulman G. L. , Corbetta M. (2007). Breakdown of Functional Connectivity in Frontoparietal Networks Underlies Behavioral Deficits in Spatial Neglect. Neuron, 53, (6), 905–918. 10.1016/j.neuron.2007.02.013 17359924

[ejn15393-bib-0100] Hecht, E. E. , Gutman, D. A. , Bradley, B. A. , Preuss, T. M. , & Stout, D. (2015). Virtual dissection and comparative connectivity of the superior longitudinal fasciculus in chimpanzees and humans. NeuroImage, 108, 124–137. 10.1016/j.neuroimage.2014.12.039 25534109 PMC4324003

[ejn15393-bib-0101] Henderson, M. , & Serences, J. T. (2019). Human frontoparietal cortex represents behaviorally relevant target status based on abstract object features. Journal of Neurophysiology, 121(4), 1410–1427. 10.1152/jn.00015.2019 30759040 PMC6485745

[ejn15393-bib-0102] Hippmann, B. , Kuhlemann, I. , Bäumer, T. , Bahlmann, J. , Münte, T. F. , & Jessen, S. (2019). Boosting the effect of reward on cognitive control using TMS over the left IFJ. Neuropsychologia, 125, 109–115. 10.1016/j.neuropsychologia.2019.01.016 30721740

[ejn15393-bib-0103] Hoffman, J. E. , & Subramaniam, B. (1995). The role of visual attention in saccadic eye movements. Perception & Psychophysics, 57(6), 787–795. 10.3758/BF03206794 7651803

[ejn15393-bib-0104] Hupé, J. M. , Bordier, C. , & Dojat, M. (2012). A BOLD signature of eyeblinks in the visual cortex. NeuroImage, 61(1), 149–161. 10.1016/j.neuroimage.2012.03.001 22426351

[ejn15393-bib-0105] Itti, L. , & Koch, C. (2001). Computational modelling of visual attention. Nature Reviews Neuroscience, 2(3), 194–203. 10.1038/35058500 11256080

[ejn15393-bib-0106] Jerde, T. A. , Merriam, E. P. , Riggall, A. C. , Hedges, J. H. , & Curtis, C. E. (2012). Prioritized maps of space in human frontoparietal cortex. Journal of Neuroscience, 32(48), 17382–17390. 10.1523/JNEUROSCI.3810-12.2012 23197729 PMC3544526

[ejn15393-bib-0107] Jeurissen, B. , Descoteaux, M. , Mori, S. , & Leemans, A. (2019). Diffusion MRI fiber tractography of the brain. NMR in Biomedicine, 32(4), e3785. 10.1002/nbm.3785 28945294

[ejn15393-bib-0108] Ji, J. L. , Spronk, M. , Kulkarni, K. , Repovš, G. , Anticevic, A. , & Cole, M. W. (2019). Mapping the human brain's cortical‐subcortical functional network organization. NeuroImage, 185, 35–57. 10.1016/j.neuroimage.2018.10.006 30291974 PMC6289683

[ejn15393-bib-0109] Kastner, S. , DeSimone, K. , Konen, C. S. , Szczepanski, S. M. , Weiner, K. S. , & Schneider, K. A. (2007). Topographic maps in human frontal cortex revealed in memory‐guided saccade and spatial working‐memory tasks. Journal of Neurophysiology, 97(5), 3494–3507. 10.1152/jn.00010.2007 17360822

[ejn15393-bib-0110] Kastner, S. , & Ungerleider, L. G. (2001). The neural basis of biased competition in human visual cortex. Neuropsychologia, 39(12), 1263–1276. 10.1016/S0028-3932(01)00116-6 11566310

[ejn15393-bib-0111] Kato, M. , & Miyauchi, S. (2003). Human precentral cortical activation patterns during saccade tasks: An fMRI comparison with activation during intentional eyeblink tasks. NeuroImage, 19(4), 1260–1272. 10.1016/S1053-8119(03)00223-4 12948687

[ejn15393-bib-0112] Kim, S. Y. , Kim, M. S. , & Chun, M. M. (2005). Concurrent working memory load can reduce distraction. Proceedings of the National Academy of Sciences, 102(45), 16524–16529. 10.1073/pnas.0505454102 PMC1283430 16258067

[ejn15393-bib-0113] Kiyonaga, A. , & Egner, T. (2013). Working memory as internal attention: Toward an integrative account of internal and external selection processes. Psychonomic Bulletin & Review, 20(2), 228–242. 10.3758/s13423-012-0359-y 23233157 PMC3594067

[ejn15393-bib-0114] Koechlin, E. , Ody, C. , & Kouneiher, F. (2003). The architecture of cognitive control in the human prefrontal cortex. Science, 302(5648), 1181–1185. 10.1126/science.1088545 14615530

[ejn15393-bib-0115] Kötter, R. (2004). Online retrieval, processing, and visualization of primate connectivity data from the CoCoMac database. Neuroinformatics, 2(2), 127–144. 10.1385/NI:2:2:127 15319511

[ejn15393-bib-0116] Kowler, E. , Anderson, E. , Dosher, B. , & Blaser, E. (1995). The role of attention in the programming of saccades. Vision Research, 35(13), 1897–1916. 10.1016/0042-6989(94)00279-U 7660596

[ejn15393-bib-0117] Koyama, M. , Hasegawa, I. , Osada, T. , Adachi, Y. , Nakahara, K. , & Miyashita, Y. (2004). Functional magnetic resonance imaging of macaque monkeys performing visually guided saccade tasks: Comparison of cortical eye fields with humans. Neuron, 41(5), 795–807. 10.1016/S0896-6273(04)00047-9 15003178

[ejn15393-bib-0118] Kravitz, D. J. , Saleem, K. S. , Baker, C. I. , Ungerleider, L. G. , & Mishkin, M. (2013). The ventral visual pathway: An expanded neural framework for the processing of object quality. Trends in Cognitive Sciences, 17(1), 26–49. 10.1016/j.tics.2012.10.011 23265839 PMC3532569

[ejn15393-bib-0119] Lancaster, J. L. , Tordesillas‐Gutiérrez, D. , Martinez, M. , Salinas, F. , Evans, A. , Zilles, K. , … Fox, P. T. (2007). Bias between MNI and Talairach coordinates analyzed using the ICBM‐152 brain template. Human Brain Mapping, 28(11), 1194–1205. 10.1002/hbm.20345 17266101 PMC6871323

[ejn15393-bib-0120] LaRocque, J. J. , Lewis‐Peacock, J. A. , Drysdale, A. T. , Oberauer, K. , & Postle, B. R. (2013). Decoding attended information in short‐term memory: An EEG study. Journal of Cognitive Neuroscience, 25(1), 127–142. 10.1162/jocn_a_00305 23198894 PMC3775605

[ejn15393-bib-0121] Lawrence, B. M. , Myerson, J. , & Abrams, R. A. (2004). Interference with spatial working memory: An eye movement is more than a shift of attention. Psychonomic Bulletin & Review, 11, 488–494. 10.3758/BF03196600 15376800

[ejn15393-bib-0122] Lepsien, J. , Thornton, I. , & Nobre, A. C. (2011). Modulation of working‐memory maintenance by directed attention. Neuropsychologia, 49, 1569–1577. 10.1016/j.neuropsychologia.2011.03.011 21420421

[ejn15393-bib-0123] Liu, T. (2016). Neural representation of object‐specific attentional priority. NeuroImage, 129, 15–24. 10.1016/j.neuroimage.2016.01.034 26825437 PMC4803527

[ejn15393-bib-0124] Liu, T. , Hospadaruk, L. , Zhu, D. C. , & Gardner, J. L. (2011). Feature‐specific attentional priority signals in human cortex. Journal of Neuroscience, 31(12), 4484–4495. 10.1523/JNEUROSCI.5745-10.2011 21430149 PMC6622917

[ejn15393-bib-0125] Lowet, E. , Gomes, B. , Srinivasan, K. , Zhou, H. , Schafer, R. J. , & Desimone, R. (2018). Enhanced neural processing by covert attention only during microsaccades directed toward the attended stimulus. Neuron, 99(1), 207–214. 10.1016/j.neuron.2018.05.041 29937279 PMC8415255

[ejn15393-bib-0126] Luria, A. R. (1966). Higher cortical functions in man. Basic Books.

[ejn15393-bib-0127] Macaluso, E. , & Doricchi, F. (2013). Attention and predictions: Control of spatial attention beyond the endogenous‐exogenous dichotomy. Frontiers in Human Neuroscience, 7, 685. 10.3389/fnhum.2013.00685 24155707 PMC3800774

[ejn15393-bib-0128] Mackey, W. E. , Winawer, J. , & Curtis, C. E. (2017). Visual field map clusters in human frontoparietal cortex. eLife, 6, 1–23. 10.7554/eLife.22974 PMC5491263 28628004

[ejn15393-bib-0129] Maier‐Hein, K. H. , Neher, P. F. , Houde, J. C. , Côté, M. A. , Garyfallidis, E. , Zhong, J. , … Reddick, W. E. (2017). The challenge of mapping the human connectome based on diffusion tractography. Nature Communications, 8(1), 1–13. 10.1038/s41467-017-01285-x PMC5677006 29116093

[ejn15393-bib-0130] Markov, N. T. , Ercsey‐Ravasz, M. M. , Ribeiro Gomes, A. R. , Lamy, C. , Magrou, L. , Vezoli, J. , … Sallet, J. (2014). A weighted and directed interareal connectivity matrix for macaque cerebral cortex. Cerebral Cortex, 24(1), 17–36. 10.1093/cercor/bhs270 23010748 PMC3862262

[ejn15393-bib-0131] Mars, R. B. , Passingham, R. E. , & Jbabdi, S. (2018). Connectivity fingerprints: From areal descriptions to abstract spaces. Trends in Cognitive Sciences, 22(11), 1026–1037. 10.1016/j.tics.2018.08.009 30241910 PMC6198109

[ejn15393-bib-0132] Mars, R. B. , Sotiropoulos, S. N. , Passingham, R. E. , Sallet, J. , Verhagen, L. , Khrapitchev, A. A. , … Jbabdi, S. (2018). Whole brain comparative anatomy using connectivity blueprints. eLife, 7, e35237. 10.7554/eLife.35237.013 29749930 PMC5984034

[ejn15393-bib-0133] Marshall, T. R. , O'Shea, J. , Jensen, O. , & Bergmann, T. O. (2015). Frontal eye fields control attentional modulation of alpha and gamma oscillations in contralateral occipitoparietal cortex. Journal of Neuroscience, 35(4), 1638–1647. 10.1523/JNEUROSCI.3116-14.2015 25632139 PMC4308606

[ejn15393-bib-0134] Martin, A. B. , Yang, X. , Saalmann, Y. B. , Wang, L. , Shestyuk, A. , Lin, J. J. , … Kastner, S. (2019). Temporal dynamics and response modulation across the human visual system in a spatial attention task: An ECoG study. Journal of Neuroscience, 39(2), 333–352. 10.1523/JNEUROSCI.1889-18.2018 30459219 PMC6325255

[ejn15393-bib-0135] Mendoza‐Halliday, D. , & Martinez‐Trujillo, J. C. (2017). Neuronal population coding of perceived and memorized visual features in the lateral prefrontal cortex. Nature Communications, 8(1), 1–13. 10.1038/ncomms15471 PMC5461493 28569756

[ejn15393-bib-0136] Meyer, T. , Qi, X. L. , & Constantinidis, C. (2007). Persistent discharges in the prefrontal cortex of monkeys naive to working memory tasks. Cerebral Cortex, 17(suppl_1), i70–i76. 10.1093/cercor/bhm063 17726005

[ejn15393-bib-0137] Meyer, T. , Qi, X. L. , Stanford, T. R. , & Constantinidis, C. (2011). Stimulus selectivity in dorsal and ventral prefrontal cortex after training in working memory tasks. Journal of Neuroscience, 31(17), 6266–6276. 10.1523/JNEUROSCI.6798-10.2011 21525266 PMC3103869

[ejn15393-bib-0138] Meyyappan, S. , Rajan, A. , Mangun, G. R. , & Ding, M. (2020). Role of inferior frontal junction (IFJ) in the control of feature vs spatial attention. bioRxiv. 10.1101/2020.11.04.368993 PMC8460144 34380762

[ejn15393-bib-0139] Michalareas, G. , Vezoli, J. , Van Pelt, S. , Schoffelen, J. M. , Kennedy, H. , & Fries, P. (2016). Alpha‐beta and gamma rhythms subserve feedback and feedforward influences among human visual cortical areas. Neuron, 89(2), 384–397. 10.1016/j.neuron.2015.12.018 26777277 PMC4871751

[ejn15393-bib-0140] Michalka, S. W. , Kong, L. , Rosen, M. L. , Shinn‐Cunningham, B. G. , & Somers, D. C. (2015). Short‐term memory for space and time flexibly recruit complementary sensory‐biased frontal lobe attention networks. Neuron, 87(4), 882–892. 10.1016/j.neuron.2015.07.028 26291168 PMC4545499

[ejn15393-bib-0141] Milham, M. P. , Ai, L. , Koo, B. , Xu, T. , Amiez, C. , Balezeau, F. , … Croxson, P. L. (2018). An open resource for non‐human primate imaging. Neuron, 100(1), 61–74. 10.1016/j.neuron.2018.08.039 30269990 PMC6231397

[ejn15393-bib-0142] Miller, E. K. , & Cohen, J. D. (2001). An integrative theory of prefrontal cortex function. Annual Review of Neuroscience, 24(1), 167–202. 10.1146/annurev.neuro.24.1.167 11283309

[ejn15393-bib-0143] Mishkin, M. , Ungerleider, L. G. , & Macko, K. A. (1983). Object vision and spatial vision: Two cortical pathways. Trends in Neurosciences, 6, 414–417. 10.1016/0166-2236(83)90190-X

[ejn15393-bib-0144] Moore, T. , & Armstrong, K. M. (2003). Selective gating of visual signals by microstimulation of frontal cortex. Nature, 421(6921), 370–373. 10.1038/nature01341 12540901

[ejn15393-bib-0145] Moore, T. , & Fallah, M. (2001). Control of eye movements and spatial attention. Proceedings of the National Academy of Sciences, 98(3), 1273–1276. 10.1073/pnas.98.3.1273 PMC14744 11158629

[ejn15393-bib-0146] Moore, T. , & Zirnsak, M. (2015). The what and where of visual attention. Neuron, 88(4), 626–628. 10.1016/j.neuron.2015.11.005 26590339

[ejn15393-bib-0147] Muhle‐Karbe, P. S. , Andres, M. , & Brass, M. (2014). Transcranial magnetic stimulation dissociates prefrontal and parietal contributions to task preparation. Journal of Neuroscience, 34(37), 12481–12489. 10.1523/JNEUROSCI.4931-13.2014 25209286 PMC6615496

[ejn15393-bib-0148] Muhle‐Karbe, P. S. , Derrfuss, J. , Lynn, M. T. , Neubert, F. X. , Fox, P. T. , Brass, M. , & Eickhoff, S. B. (2016). Co‐activation‐based parcellation of the lateral prefrontal cortex delineates the inferior frontal junction area. Cerebral Cortex, 26(5), 2225–2241. 10.1093/cercor/bhv073 25899707 PMC4830296

[ejn15393-bib-0149] Muhle‐Karbe, P. S. , Duncan, J. , De Baene, W. , Mitchell, D. J. , & Brass, M. (2017). Neural coding for instruction‐based task sets in human frontoparietal and visual cortex. Cerebral Cortex, 27(3), 1891–1905. 10.1093/cercor/bhw032 26908634 PMC6606446

[ejn15393-bib-0150] Muhle‐Karbe, P. S. , Jiang, J. , & Egner, T. (2018). Causal evidence for learning‐dependent frontal lobe contributions to cognitive control. Journal of Neuroscience, 38(4), 962–973. 10.1523/JNEUROSCI.1467-17.2017 29229706 PMC5783969

[ejn15393-bib-0151] Nee, D. E. , & D'Esposito, M. (2016). The hierarchical organization of the lateral prefrontal cortex. eLife, 5, e12112. 10.7554/eLife.12112.002 26999822 PMC4811776

[ejn15393-bib-0152] Neggers, S. F. , Huijbers, W. , Vrijlandt, C. M. , Vlaskamp, B. N. , Schutter, D. J. , & Kenemans, J. L. (2007). TMS pulses on the frontal eye fields break coupling between visuospatial attention and eye movements. Journal of Neurophysiology, 98(5), 2765–2778. 10.1152/jn.00357.2007 17699696

[ejn15393-bib-0153] Neubert, F. X. , Mars, R. B. , Thomas, A. G. , Sallet, J. , & Rushworth, M. F. S. (2014). Comparison of human ventral frontal cortex areas for cognitive control and language with areas in monkey frontal cortex. Neuron, 81(3), 700–713. 10.1016/j.neuron.2013.11.012 24485097

[ejn15393-bib-0154] Ngo, G. H. , Eickhoff, S. B. , Nguyen, M. , Sevinc, G. , Fox, P. T. , Spreng, R. N. , & Yeo, B. T. (2019). Beyond consensus: Embracing heterogeneity in curated neuroimaging meta‐analysis. NeuroImage, 200, 142–158. 10.1016/j.neuroimage.2019.06.037 31229658 PMC6703957

[ejn15393-bib-0155] Norman, D. A. , & Shallice, T. (1986). Attention to action. In Consciousness and self‐regulation (pp. 1–18). Springer. 10.1007/978-1-4757-0629-1_1

[ejn15393-bib-0156] Noyce, A. L. , Cestero, N. , Michalka, S. W. , Shinn‐Cunningham, B. G. , & Somers, D. C. (2017). Sensory‐biased and multiple‐demand processing in human lateral frontal cortex. Journal of Neuroscience, 37(36), 8755–8766. 10.1523/JNEUROSCI.0660-17.2017 28821668 PMC5588466

[ejn15393-bib-0157] Oberauer, K. (2019). Working memory and attention—A conceptual analysis and review. Journal of Cognition, 2(1). 36 10.5334/joc.58 31517246 PMC6688548

[ejn15393-bib-0158] Olivers, C. N. (2008). Interactions between visual working memory and visual attention. Frontiers in Bioscience, 13(3), 1182–1191. 10.2741/2754 17981622

[ejn15393-bib-0159] O'Reilly, R. C. (2010). The what and how of prefrontal cortical organization. Trends in Neurosciences, 33(8), 355–361. 10.1016/j.tins.2010.05.002 20573407 PMC2916029

[ejn15393-bib-0160] Osher, D. E. , Saxe, R. R. , Koldewyn, K. , Gabrieli, J. D. , Kanwisher, N. , & Saygin, Z. M. (2016). Structural connectivity fingerprints predict cortical selectivity for multiple visual categories across cortex. Cerebral Cortex, 26(4), 1668–1683. 10.1093/cercor/bhu303 25628345 PMC4785945

[ejn15393-bib-0161] Owen, A. M. , Stern, C. E. , Look, R. B. , Tracey, I. , Rosen, B. R. , & Petrides, M. (1998). Functional organization of spatial and nonspatial working memory processing within the human lateral frontal cortex. Proceedings of the National Academy of Sciences, 95(13), 7721–7726. 10.1073/pnas.95.13.7721 PMC22736 9636217

[ejn15393-bib-0162] Parlatini, V. , Radua, J. , Dell'Acqua, F. , Leslie, A. , Simmons, A. , Murphy, D. G. , … de Schotten, M. T. (2017). Functional segregation and integration within fronto‐parietal networks. NeuroImage, 146, 367–375. 10.1016/j.neuroimage.2016.08.031 27639357 PMC5312783

[ejn15393-bib-0163] Pashler, H. (1994). Dual‐task interference in simple tasks: Data and theory. Psychological Bulletin, 116(2), 220–244. 10.1037/0033-2909.116.2.220 7972591

[ejn15393-bib-0164] Passingham, R. E. , Stephan, K. E. , & Kötter, R. (2002). The anatomical basis of functional localization in the cortex. Nature Reviews Neuroscience, 3(8), 606–616. 10.1038/nrn893 12154362

[ejn15393-bib-0165] Paus, T. (1996). Location and function of the human frontal eye‐field: A selective review. Neuropsychologia, 34(6), 475–483. 10.1016/0028-3932(95)00134-4 8736560

[ejn15393-bib-0166] Penfield, W. , & Rasmussen, T. (1950). The cerebral cortex of man; a clinical study of localization of function. Macmillan.

[ejn15393-bib-0167] Petit, L. , & Pouget, P. (2019). The comparative anatomy of frontal eye fields in primates. Cortex, 118, 51–64. 10.1016/j.cortex.2019.02.023 30979504

[ejn15393-bib-0168] Petrides, M. , & Pandya, D. N. (1999). Dorsolateral prefrontal cortex: Comparative cytoarchitectonic analysis in the human and the macaque brain and corticocortical connection patterns. European Journal of Neuroscience, 11(3), 1011–1036. 10.1046/j.1460-9568.1999.00518.x 10103094

[ejn15393-bib-0169] Petrides, M. , & Pandya, D. N. (2002). Comparative cytoarchitectonic analysis of the human and the macaque ventrolateral prefrontal cortex and corticocortical connection patterns in the monkey. European Journal of Neuroscience, 16(2), 291–310. 10.1046/j.1460-9568.2001.02090.x 12169111

[ejn15393-bib-0170] Petrides, M. , Tomaiuolo, F. , Yeterian, E. H. , & Pandya, D. N. (2012). The prefrontal cortex: Comparative architectonic organization in the human and the macaque monkey brains. Cortex, 48(1), 46–57. 10.1016/j.cortex.2011.07.002 21872854

[ejn15393-bib-0171] Popov, T. , Kastner, S. , & Jensen, O. (2017). FEF‐controlled alpha delay activity precedes stimulus‐induced gamma‐band activity in visual cortex. Journal of Neuroscience, 37(15), 4117–4127. 10.1523/JNEUROSCI.3015-16.2017 28314817 PMC5391684

[ejn15393-bib-0172] Posner, M. I. (1980). Orienting of attention. Quarterly Journal of Experimental Psychology, 32(1), 3–25. 10.1080/00335558008248231 7367577

[ejn15393-bib-0173] Rainer, G. , Asaad, W. F. , & Miller, E. K. (1998). Selective representation of relevant information by neurons in the primate prefrontal cortex. Nature, 393(6685), 577–579. 10.1038/31235 9634233

[ejn15393-bib-0174] Rao, S. C. , Rainer, G. , & Miller, E. K. (1997). Integration of what and where in the primate prefrontal cortex. Science, 276(5313), 821–824. 10.1126/science.276.5313.821 9115211

[ejn15393-bib-0175] Riley, M. R. , Qi, X. L. , & Constantinidis, C. (2017). Functional specialization of areas along the anterior–posterior axis of the primate prefrontal cortex. Cerebral Cortex, 27(7), 3683–3697. 10.1093/cercor/bhw190 27371761 PMC6059101

[ejn15393-bib-0176] Rizzolatti, G. , Riggio, L. , Dascola, I. , & Umiltá, C. (1987). Reorienting attention across the horizontal and vertical meridians: Evidence in favor of a premotor theory of attention. Neuropsychologia, 25(1), 31–40. 10.1016/0028-3932(87)90041-8 3574648

[ejn15393-bib-0177] Robinson, E. C. , Jbabdi, S. , Glasser, M. F. , Andersson, J. , Burgess, G. C. , Harms, M. P. , … Jenkinson, M. (2014). MSM: A new flexible framework for Multimodal Surface Matching. NeuroImage, 100, 414–426. 10.1016/j.neuroimage.2014.05.069 24939340 PMC4190319

[ejn15393-bib-0178] Robinson, J. L. , Laird, A. R. , Glahn, D. C. , Lovallo, W. R. , & Fox, P. T. (2010). Metaanalytic connectivity modeling: Delineating the functional connectivity of the human amygdala. Human Brain Mapping, 31(2), 173–184. 10.1002/hbm.20854 19603407 PMC2872058

[ejn15393-bib-0179] Romanski, L. M. (2004). Domain specificity in the primate prefrontal cortex. Cognitive, Affective, & Behavioral Neuroscience, 4(4), 421–429. 10.3758/CABN.4.4.421 15849888

[ejn15393-bib-0180] Ronconi, L. , Basso, D. , Gori, S. , & Facoetti, A. (2014). TMS on right frontal eye fields induces an inflexible focus of attention. Cerebral Cortex, 24(2), 396–402. 10.1093/cercor/bhs319 23048022

[ejn15393-bib-0181] Rosano, C. , Sweeney, J. A. , Melchitzky, D. S. , & Lewis, D. A. (2003). The human precentral sulcus: Chemoarchitecture of a region corresponding to the frontal eye fields. Brain Research, 972(1–2), 16–30. 10.1016/S0006-8993(03)02431-4 12711074

[ejn15393-bib-0182] Rottschy, C. , Langner, R. , Dogan, I. , Reetz, K. , Laird, A. R. , Schulz, J. B. , … Eickhoff, S. B. (2012). Modelling neural correlates of working memory: A coordinate‐based meta‐analysis. NeuroImage, 60(1), 830–846. 10.1016/j.neuroimage.2011.11.050 22178808 PMC3288533

[ejn15393-bib-0183] Ruff, C. C. , Blankenburg, F. , Bjoertomt, O. , Bestmann, S. , Freeman, E. , Haynes, J. D. , … Driver, J. (2006). Concurrent TMS‐fMRI and psychophysics reveal frontal influences on human retinotopic visual cortex. Current Biology, 16(15), 1479–1488. 10.1016/j.cub.2006.06.057 16890523

[ejn15393-bib-0184] Sallet, J. , Mars, R. B. , Noonan, M. P. , Neubert, F. X. , Jbabdi, S. , O'Reilly, J. X. , … Rushworth, M. F. (2013). The organization of dorsal frontal cortex in humans and macaques. Journal of Neuroscience, 33(30), 12255–12274. 10.1523/JNEUROSCI.5108-12.2013 23884933 PMC3744647

[ejn15393-bib-0185] Saygin, A. P. , & Sereno, M. I. (2008). Retinotopy and attention in human occipital, temporal, parietal, and frontal cortex. Cerebral Cortex, 18(9), 2158–2168. 10.1093/cercor/bhm242 18234687

[ejn15393-bib-0186] Saygin, Z. M. , Osher, D. E. , Koldewyn, K. , Reynolds, G. , Gabrieli, J. D. , & Saxe, R. R. (2012). Anatomical connectivity patterns predict face selectivity in the fusiform gyrus. Nature Neuroscience, 15(2), 321–327. 10.1038/nn.3001 PMC3267901 22197830

[ejn15393-bib-0187] Scalaidhe, S. P. Ó. , Wilson, F. A. , & Goldman‐Rakic, P. S. (1997). Areal segregation of face‐processing neurons in prefrontal cortex. Science, 278(5340), 1135–1138. 10.1126/science.278.5340.1135 9353197

[ejn15393-bib-0188] Scalaidhe, S. P. Ó. , Wilson, F. A. W. , & Goldman‐Rakic, P. S. (1999). Face‐selective neurons during passive viewing and working memory performance of rhesus monkeys: Evidence for intrinsic specialization of neuronal coding. Cerebral Cortex, 9(5), 459–475. 10.1093/cercor/9.5.459 10450891

[ejn15393-bib-0189] Schall, J. D. (2015). Visuomotor functions in the frontal lobe. Annual Review of Vision Science, 1, 469–498. 10.1146/annurev-vision-082114-035317 28532381

[ejn15393-bib-0190] Schall, J. D. , Morel, A. , King, D. J. , & Bullier, J. (1995). Topography of visual cortex connections with frontal eye field in macaque: Convergence and segregation of processing streams. Journal of Neuroscience, 15(6), 4464–4487. 10.1523/JNEUROSCI.15-06-04464.1995 7540675 PMC6577698

[ejn15393-bib-0191] Schmitt, O. , Modersitzki, J. , Heldmann, S. , Wirtz, S. , Hömke, L. , Heide, W. , … Wree, A. (2005). Three‐dimensional cytoarchitectonic analysis of the posterior bank of the human precentral sulcus. Anatomy and Embryology, 210(5), 387–400. 10.1007/s00429-005-0030-8 16177908

[ejn15393-bib-0192] Schwedhelm, P. , Baldauf, D. , & Treue, S. (2017). Electrical stimulation of macaque lateral prefrontal cortex modulates oculomotor behavior indicative of a disruption of top‐down attention. Scientific Reports, 7(1), 1–10. 10.1038/s41598-017-18153-9 29255155 PMC5735183

[ejn15393-bib-0193] Schwedhelm, P. , Baldauf, D. , & Treue, S. (2020). The lateral prefrontal cortex of primates encodes stimulus colors and their behavioral relevance during a match‐to‐sample task. Scientific Reports, 10(1), 1–12. 10.1038/s41598-020-61171-3 32144331 PMC7060344

[ejn15393-bib-0194] Serences, J. T. (2016). Neural mechanisms of information storage in visual short‐term memory. Vision Research, 128, 53–67. 10.1016/j.visres.2016.09.010 27668990 PMC5079778

[ejn15393-bib-0195] Serences, J. T. , & Boynton, G. M. (2007). Feature‐based attentional modulations in the absence of direct visual stimulation. Neuron, 55(2), 301–312. 10.1016/j.neuron.2007.06.015 17640530

[ejn15393-bib-0196] Serences, J. T. , Schwarzbach, J. , Courtney, S. M. , Golay, X. , & Yantis, S. (2004). Control of object‐based attention in human cortex. Cerebral Cortex, 14(12), 1346–1357. 10.1093/cercor/bhh095 15166105

[ejn15393-bib-0197] Sereno, M. I. , Dale, A. M. , Reppas, J. B. , Kwong, K. K. , Belliveau, J. W. , Brady, T. J. , … Tootell, R. B. (1995). Borders of multiple visual areas in humans revealed by functional magnetic resonance imaging. Science, 268(5212), 889–893. 10.1126/science.7754376 7754376

[ejn15393-bib-0198] Seth, A. K. , Barrett, A. B. , & Barnett, L. (2015). Granger causality analysis in neuroscience and neuroimaging. Journal of Neuroscience, 35(8), 3293–3297. 10.1523/JNEUROSCI.4399-14.2015 25716830 PMC4339347

[ejn15393-bib-0199] Shulman, G. L. , Astafiev, S. V. , Franke, D. , Pope, D. L. , Snyder, A. Z. , McAvoy, M. P. , & Corbetta, M. (2009). Interaction of stimulus‐driven reorienting and expectation in ventral and dorsal frontoparietal and basal ganglia‐cortical networks. Journal of Neuroscience, 29(14), 4392–4407. 10.1523/JNEUROSCI.5609-08.2009 19357267 PMC2743562

[ejn15393-bib-0246] Shulman G. L. , Corbetta, M. , Buckner, R. L. , Raichle, M. E. , Fiez, J. A. , Miezin, F. M. , & Petersen, F. E. (1997). Top‐down modulation of early sensory cortex. Cerebral Cortex, 7(3), 193–206. 10.1093/cercor/7.3.193 9143441

[ejn15393-bib-0200] Slagter, H. A. , Giesbrecht, B. , Kok, A. , Weissman, D. H. , Kenemans, J. L. , Woldorff, M. G. , & Mangun, G. R. (2007). fMRI evidence for both generalized and specialized components of attentional control. Brain Research, 1177, 90–102. 10.1016/j.brainres.2007.07.097 17916338 PMC2710450

[ejn15393-bib-0201] Sneve, M. H. , Magnussen, S. , Alnæs, D. , Endestad, T. , & D'Esposito, M. (2013). Top‐down modulation from inferior frontal junction to FEFs and intraparietal sulcus during short‐term memory for visual features. Journal of Cognitive Neuroscience, 25(11), 1944–1956. 10.1162/jocn_a_00426 23691986

[ejn15393-bib-0202] Sreenivasan, K. K. , Curtis, C. E. , & D'Esposito, M. (2014). Revisiting the role of persistent neural activity during working memory. Trends in Cognitive Sciences, 18(2), 82–89. 10.1016/j.tics.2013.12.001 24439529 PMC3964018

[ejn15393-bib-0203] Srimal, R. , & Curtis, C. E. (2008). Persistent neural activity during the maintenance of spatial position in working memory. NeuroImage, 39(1), 455–468. 10.1016/j.neuroimage.2007.08.040 17920934 PMC2219966

[ejn15393-bib-0204] Stanton, G. B. , Bruce, C. J. , & Goldberg, M. E. (1995). Topography of projections to posterior cortical areas from the macaque frontal eye fields. Journal of Comparative Neurology, 353(2), 291–305. 10.1002/cne.903530210 7745137

[ejn15393-bib-0205] Störmer, V. S. , & Alvarez, G. A. (2014). Feature‐based attention elicits surround suppression in feature space. Current Biology, 24(17), 1985–1988. 10.1016/j.cub.2014.07.030 25155510

[ejn15393-bib-0206] Sundermann, B. , & Pfleiderer, B. (2012). Functional connectivity profile of the human inferior frontal junction: Involvement in a cognitive control network. BMC Neuroscience, 13(1), 1–13. 10.1186/1471-2202-13-119 23033990 PMC3582543

[ejn15393-bib-0207] Sylvester, C. Y. C. , Wager, T. D. , Lacey, S. C. , Hernandez, L. , Nichols, T. E. , Smith, E. E. , & Jonides, J. (2003). Switching attention and resolving interference: fMRI measures of executive functions. Neuropsychologia, 41(3), 357–370. 10.1016/S0028-3932(02)00167-7 12457760

[ejn15393-bib-0208] Szczepanski, S. M. , Crone, N. E. , Kuperman, R. A. , Auguste, K. I. , Parviz, J. , & Knight, R. T. (2014). Dynamic changes in phase‐amplitude coupling facilitate spatial attention control in fronto‐parietal cortex. PLoS Biology, 12(8), e1001936. 10.1371/journal.pbio.1001936 25157678 PMC4144794

[ejn15393-bib-0209] Szczepanski, S. M. , Pinsk, M. A. , Douglas, M. M. , Kastner, S. , & Saalmann, Y. B. (2013). Functional and structural architecture of the human dorsal frontoparietal attention network. Proceedings of the National Academy of Sciences, 110(39), 15806–15811. 10.1073/pnas.1313903110 PMC3785784 24019489

[ejn15393-bib-0210] Tabarelli, D. , Keitel, C. , Gross, J. , & Baldauf, D. (2020). Spatial attention enhances cortical tracking of quasi‐rhythmic visual stimuli. NeuroImage, 208, 116444. 10.1016/j.neuroimage.2019.116444 31816422

[ejn15393-bib-0211] Tamber‐Rosenau, B. J. , Asplund, C. L. , & Marois, R. (2018). Functional dissociation of the inferior frontal junction from the dorsal attention network in top‐down attentional control. Journal of Neurophysiology, 120(5), 2498–2512. 10.1152/jn.00506.2018 30156458 PMC6295539

[ejn15393-bib-0212] Tamber‐Rosenau, B. J. , Dux, P. E. , Tombu, M. N. , Asplund, C. L. , & Marois, R. (2013). Amodal processing in human prefrontal cortex. Journal of Neuroscience, 33(28), 11573–11587. 10.1523/JNEUROSCI.4601-12.2013 23843526 PMC3724542

[ejn15393-bib-0213] Tavor, I. , Jones, O. P. , Mars, R. B. , Smith, S. M. , Behrens, T. E. , & Jbabdi, S. (2016). Task‐free MRI predicts individual differences in brain activity during task performance. Science, 352(6282), 216–220. 10.1126/science.aad8127 27124457 PMC6309730

[ejn15393-bib-0214] Taylor, P. C. , Nobre, A. C. , & Rushworth, M. F. (2007). FEF TMS affects visual cortical activity. Cerebral Cortex, 17(2), 391–399. 10.1093/cercor/bhj156 16525126

[ejn15393-bib-0215] Tehovnik, E. J. , Sommer, M. A. , Chou, I. H. , Slocum, W. M. , & Schiller, P. H. (2000). Eye fields in the frontal lobes of primates. Brain Research Reviews, 32(2–3), 413–448. 10.1016/S0165-0173(99)00092-2 10760550

[ejn15393-bib-0216] Thompson, K. G. , & Bichot, N. P. (2005). A visual salience map in the primate frontal eye field. Progress in Brain Research, 147, 249–262. 10.1016/S0079-6123(04)47019-8 15581711

[ejn15393-bib-0217] Thompson, K. G. , Bichot, N. P. , & Schall, J. D. (1997). Dissociation of visual discrimination from saccade programming in macaque frontal eye field. Journal of Neurophysiology, 77(2), 1046–1050. 10.1152/jn.1997.77.2.1046 9065870

[ejn15393-bib-0218] Tobyne, S. M. , Osher, D. E. , Michalka, S. W. , & Somers, D. C. (2017). Sensory‐biased attention networks in human lateral frontal cortex revealed by intrinsic functional connectivity. NeuroImage, 162, 362–372. 10.1016/j.neuroimage.2017.08.020 28830764 PMC5705425

[ejn15393-bib-0219] Umarova, R. M. , Saur, D. , Schnell, S. , Kaller, C. P. , Vry, M. S. , Glauche, V. , … Weiller, C. (2010). Structural connectivity for visuospatial attention: Significance of ventral pathways. Cerebral Cortex, 20(1), 121–129. 10.1093/cercor/bhp086 19406904

[ejn15393-bib-0220] van den Heuvel, M. P. , de Reus, M. A. , Feldman Barrett, L. , Scholtens, L. H. , Coopmans, F. M. , Schmidt, R. , … Li, L. (2015). Comparison of diffusion tractography and tract‐tracing measures of connectivity strength in rhesus macaque connectome. Human Brain Mapping, 36(8), 3064–3075. 10.1002/hbm.22828 26058702 PMC6869766

[ejn15393-bib-0221] Van Essen, D. C. , Donahue, C. J. , Coalson, T. S. , Kennedy, H. , Hayashi, T. , & Glasser, M. F. (2019). Cerebral cortical folding, parcellation, and connectivity in humans, nonhuman primates, and mice. Proceedings of the National Academy of Sciences of the United States of America, 116(52), 26173–26180. 10.1073/pnas.1902299116 PMC6936571 31871175

[ejn15393-bib-0222] Van Essen, D. C. , Smith, J. , Glasser, M. F. , Elam, J. , Donahue, C. J. , Dierker, D. L. , … Harwell, J. (2017). The brain analysis library of spatial maps and atlases (BALSA) database. NeuroImage, 144, 270–274. 10.1016/j.neuroimage.2016.04.002 27074495 PMC5149446

[ejn15393-bib-0223] Van Essen, D. C. , Smith, S. M. , Barch, D. M. , Behrens, T. E. , Yacoub, E. , Ugurbil, K. , & Wu‐Minn HCP Consortium . (2013). The WU‐Minn Human Connectome Project: An overview. NeuroImage, 80, 62–79. 10.1016/j.neuroimage.2013.05.041 23684880 PMC3724347

[ejn15393-bib-0224] VanRullen, R. (2016). Perceptual cycles. Trends in Cognitive Sciences, 20(10), 723–735. 10.1016/j.tics.2016.07.006 27567317

[ejn15393-bib-0225] Veniero, D. , Gross, J. , Morand, S. , Duecker, F. , Sack, A. T. , & Thut, G. (2021). Top‐down control of visual cortex by the frontal eye fields through oscillatory realignment. Nature Communications, 12(1), 1–13. 10.1038/s41467-021-21979-7 PMC7979788 33741947

[ejn15393-bib-0226] Verbruggen, F. , Aron, A. R. , Stevens, M. A. , & Chambers, C. D. (2010). Theta burst stimulation dissociates attention and action updating in human inferior frontal cortex. Proceedings of the National Academy of Sciences, 107(31), 13966–13971. 10.1073/pnas.1001957107 PMC2922216 20631303

[ejn15393-bib-0227] Vernet, M. , Quentin, R. , Chanes, L. , Mitsumasu, A. , & Valero‐Cabré, A. (2014). Frontal eye field, where art thou? Anatomy, function, and non‐invasive manipulation of frontal regions involved in eye movements and associated cognitive operations. Frontiers in Integrative Neuroscience, 8, 66. 10.3389/fnint.2014.00066 25202241 PMC4141567

[ejn15393-bib-0228] Vincent, J. L. , Kahn, I. , Snyder, A. Z. , Raichle, M. E. , & Buckner, R. L. (2008). Evidence for a frontoparietal control system revealed by intrinsic functional connectivity. Journal of Neurophysiology, 100(6), 3328–3342. 10.1152/jn.90355.2008 18799601 PMC2604839

[ejn15393-bib-0229] Vossel, S. , Weidner, R. , Driver, J. , Friston, K. J. , & Fink, G. R. (2012). Deconstructing the architecture of dorsal and ventral attention systems with dynamic causal modeling. Journal of Neuroscience, 32(31), 10637–10648. 10.1523/jneurosci.0414-12.2012 22855813 PMC3432566

[ejn15393-bib-0230] Wager, T. D. , & Smith, E. E. (2003). Neuroimaging studies of working memory. Cognitive, Affective, & Behavioral Neuroscience, 3(4), 255–274. 10.3758/CABN.3.4.255 15040547

[ejn15393-bib-0231] Walker, A. E. (1940). A cytoarchitectural study of the prefrontal area of the macaque monkey. Journal of Comparative Neurology, 73(1), 59–86. 10.1002/cne.900730106

[ejn15393-bib-0232] Wang, L. , Mruczek, R. E. B. , Arcaro, M. J. , & Kastner, S. (2015). Probabilistic maps of visual topography in human cortex. Cerebral Cortex, 25(10), 3911–3931. 10.1093/cercor/bhu277 25452571 PMC4585523

[ejn15393-bib-0233] Webster, M. J. , Bachevalier, J. , & Ungerleider, L. G. (1994). Connections of inferior temporal areas TEO and TE with parietal and frontal cortex in macaque monkeys. Cerebral Cortex, 4(5), 470–483. 10.1093/cercor/4.5.470 7530521

[ejn15393-bib-0234] Wen, X. , Yao, L. , Liu, Y. , & Ding, M. (2012). Causal interactions in attention networks predict behavioral performance. Journal of Neuroscience, 32(4), 1284–1292. 10.1523/JNEUROSCI.2817-11.2012 22279213 PMC6796284

[ejn15393-bib-0235] White, B. J. , Berg, D. J. , Kan, J. Y. , Marino, R. A. , Itti, L. , & Munoz, D. P. (2017). Superior colliculus neurons encode a visual saliency map during free viewing of natural dynamic video. Nature Communications, 8(1), 1–9. 10.1038/ncomms14263 PMC5286207 28117340

[ejn15393-bib-0236] Wilson, F. A. , Scalaidhe, S. P. , & Goldman‐Rakic, P. S. (1993). Dissociation of object and spatial processing domains in primate prefrontal cortex. Science, 260(5116), 1955–1958. 10.1126/science.8316836 8316836

[ejn15393-bib-0237] Xu, T. , Nenning, K. H. , Schwartz, E. , Hong, S. J. , Vogelstein, J. T. , Goulas, A. , … Milham, M. P. (2020). Cross‐species functional alignment reveals evolutionary hierarchy within the connectome. NeuroImage, 223, 117346. 10.1016/j.neuroimage.2020.117346 32916286 PMC7871099

[ejn15393-bib-0238] Yeo, B. T. , Krienen, F. M. , Sepulcre, J. , Sabuncu, M. R. , Lashkari, D. , Hollinshead, M. , … Fischl, B. (2011). The organization of the human cerebral cortex estimated by intrinsic functional connectivity. Journal of Neurophysiology, 106(3), 1125–1165. 10.1152/jn.00338.2011 21653723 PMC3174820

[ejn15393-bib-0239] Yeterian, E. H. , Pandya, D. N. , Tomaiuolo, F. , & Petrides, M. (2012). The cortical connectivity of the prefrontal cortex in the monkey brain. Cortex, 48(1), 58–81. 10.1016/j.cortex.2011.03.004 21481342 PMC3161133

[ejn15393-bib-0240] Zanto, T. P. , Rubens, M. T. , Bollinger, J. , & Gazzaley, A. (2010). Top‐down modulation of visual feature processing: The role of the inferior frontal junction. NeuroImage, 53(2), 736–745. 10.1016/j.neuroimage.2010.06.012 20600999 PMC2930130

[ejn15393-bib-0241] Zanto, T. P. , Rubens, M. T. , Thangavel, A. , & Gazzaley, A. (2011). Causal role of the prefrontal cortex in top‐down modulation of visual processing and working memory. Nature Neuroscience, 14(5), 656–661. 10.1038/nn.2773 21441920 PMC3083493

[ejn15393-bib-0242] Zelinsky, G. J. , & Bisley, J. W. (2015). The what, where, and why of priority maps and their interactions with visual working memory. Annals of the New York Academy of Sciences, 1339(1), 154–164. 10.1111/nyas.12606 25581477 PMC4376606

[ejn15393-bib-0243] Zhang, X. , Mlynaryk, N. , Ahmed, S. , Japee, S. , & Ungerleider, L. G. (2018). The role of inferior frontal junction in controlling the spatially global effect of feature‐based attention in human visual areas. PLoS Biology, 16(6), 1–28. 10.1371/journal.pbio.2005399 PMC6034892 29939981

[ejn15393-bib-0244] Zilles, K. , & Amunts, K. (2018). Cytoarchitectonic and receptorarchitectonic organization in Broca's region and surrounding cortex. Current Opinion in Behavioral Sciences, 21, 93–105. 10.1016/j.cobeha.2018.02.011

